# School-Based Cardiovascular Health Promotion in Adolescents: A Cluster Randomized Clinical Trial

**DOI:** 10.1001/jamacardio.2023.2231

**Published:** 2023-08-02

**Authors:** Gloria Santos-Beneit, Juan M. Fernández-Alvira, Anna Tresserra-Rimbau, Patricia Bodega, Amaya de Cos-Gandoy, Mercedes de Miguel, Sonia L. Ramírez-Garza, Emily P. Laveriano-Santos, Camila Arancibia-Riveros, Vanesa Carral, Xavier Orrit, Carla Rodríguez, Isabel Carvajal, Domenec Haro, Carles Peyra, Jesús Martínez-Gómez, Antonio Álvarez-Benavides, Ramón Estruch, Rosa M. Lamuela-Raventós, Rodrigo Fernández-Jiménez, Valentín Fuster

**Affiliations:** 1 Foundation for Science, Health and Education, 08011 Barcelona, Spain; 2 The Zena and Michael A. Wiener Cardiovascular Institute, Icahn School of Medicine at Mount Sinai, New York, New York; 3 Centro Nacional de Investigaciones Cardiovasculares, Madrid, Spain; 4 Departament de Nutrició, Ciències de l’Alimentació i Gastronomia, Xarxa d’Innovació Alimentària, Facultat de Farmàcia i Ciències de l’Alimentació, Institut de Nutrició i Seguretat Alimentària, Universitat de Barcelona, 08921 Santa Coloma de Gramenet, Spain; 5 Consorcio CIBER, M.P. Fisiopatología de la Obesidad y Nutrición, Instituto de Salud Carlos III, Madrid, Spain; 6 Departamento de Sociología III: Tendencias Sociales, Facultad de Ciencias Políticas y Sociología, Universidad Nacional de Educación a Distancia, Madrid, Spain; 7 Department of Internal Medicine, Hospital Clínic, Institut d’Investigacions Biomèdiques August Pi I Sunyer, University of Barcelona, Barcelona, Spain; 8 Centro de Investigación Biomédica En Red en enfermedades CardioVasculares, Madrid, Spain; 9 Hospital Universitario Clínico San Carlos, Madrid, Spain

## Abstract

**Importance:**

School-based interventions offer an opportunity for health promotion in adolescence.

**Objective:**

To assess the effect of 2 multicomponent educational health promotion strategies of differing duration and intensity on adolescents’ cardiovascular health (CVH).

**Design, Setting, and Participants:**

The SI! Program for Secondary Schools is a 4-year cluster randomized clinical intervention trial conducted in 24 secondary schools from Barcelona and Madrid, Spain, from September 7, 2017, to July 31, 2021. Eligible participants were adolescents enrolled in the first grade of secondary school.

**Interventions:**

Schools and their participants were randomized to receive a health promotion intervention (SI! Program) over 4 school years (long-term intervention [LTI], 8 schools, 412 adolescents) or 2 school years (short-term intervention [STI], 8 schools, 504 adolescents) or to receive the standard curriculum (control, 8 schools, 441 adolescents).

**Main outcome and Measures:**

The primary end point was the between-group difference at 2 and 4 years in the change from baseline of the overall CVH score, as defined by the American Heart Association (range, 0-14 points, with a higher score indicating a healthier CVH profile). Intervention effects were tested with multilevel mixed-effects models. A complete-case intention-to-treat analysis was performed as the primary analysis.

**Results:**

Of the randomized students, the study enrolled 1326 adolescents (684 [51.6%] boys, mean [SD] age, 12.5 [0.4] years at recruitment) with a study completion rate of 86.0%. Baseline overall CVH scores were 10.3 points in the LTI group, 10.6 points in the STI group, and 10.5 points in the control group. After 2 years, at halfway through the LTI and at the end of the STI, the difference in the CVH score change was 0.44 points (95% CI, 0.01-0.87; *P* = .04) between the LTI group and the control group and 0.18 points (95% CI, −0.25 to 0.61; *P* = .39) between the STI group and the control group. At 4 years, differences for the LTI and STI groups vs control were 0.12 points (LTI: 95% CI, −0.19 to 0.43; *P* = .42) and 0.13 points (STI: 95% CI, −0.17 to 0.44; *P* = .38). No adverse events were reported.

**Conclusions and Relevance:**

Overall, the tested school-based health promotion strategies in this randomized clinical trial had a neutral effect on the CVH of the adolescents. Although there was evidence of a marginal beneficial effect at a point halfway through implementation in the LTI group, such a benefit was not noted at 4 years. Further research is warranted into the efficacy of school-based health promotion programs.

**Trial Registration:**

ClinicalTrials.gov Identifier: NCT03504059

## Key Points

**Question:**

What is the effect of 2 multicomponent educational health promotion strategies of differing duration and intensity on adolescents’ cardiovascular health?

**Findings:**

In this cluster randomized clinical trial including 24 secondary schools in Spain, a neutral effect on adolescents’ cardiovascular health was found regardless of the received intervention. Although there was evidence of a marginal beneficial effect at a time point halfway through implementation in the group who received the longer intervention, this was not sustained at 4 years.

**Meaning:**

Further research is warranted into the efficacy of school-based health promotion programs with different intensities and reintervention strategies.

## Teaser

This randomized clinical trial compares the use of school-based health promotion interventions in adolescents in 2- vs 4-year programs, including a control group with the standard curriculum.

## Introduction

Cardiovascular (CV) disorders, principally ischemic heart disease and stroke, remain the leading cause of premature death and morbidity worldwide, mainly due to the high prevalence of unhealthy lifestyles and overweight and obesity.^1^ Modifiable CV risk factors include high body mass index, elevated blood pressure, smoking, and an adverse lipid profile. A recent longitudinal study found that the presence of these CV risk factors from early childhood is associated with incident CV events and death from CV causes in midlife.^2^ The same study also found that changes to these risk factors between early life stages and adulthood are important predictors of the risk of CV events later in life. This finding is consistent with prior evidence suggesting that overweight during puberty increases the risk of type 2 diabetes in middle and late adulthood.^3^

Adolescence is a crucial stage during which lifestyle choices become settled.^4,5^ There is therefore a need for early preventive action on modifiable factors (eg, diet, physical activity [PA], tobacco use, and other substance use) to stem the adverse trends in CV health (CVH).^6,7^ Schools are a favorable environment for this type of intervention.^5,8,9^ However, to our knowledge, there have been few school-based health promotion trials conducted with adolescents, and most have focused on weight loss rather than overall CVH promotion, showing only modest improvements.^10,11^ The Salud Integral Program (SI! Program) is a multidimensional educational intervention aimed at promoting lifelong CVH by instilling healthy lifestyle behaviors from early childhood through adolescence, while also involving families, teachers, and the school environment.^12,13^ Based on previous SI! Program studies in preschoolers, the ideal timing to achieve sustained positive effects may depend on multiple factors, such as the intervention duration and intensity and especially the age of the targeted population.^14^ This article reports the main results of the SI! Program for Secondary Schools trial in adolescence in Spain. The main aim of this randomized clinical trial was to assess the effect of 2 multicomponent educational health promotion strategies of differing duration and intensity on adolescents’ CVH.

## Methods

### Study Design and Population

**Figure 1.**
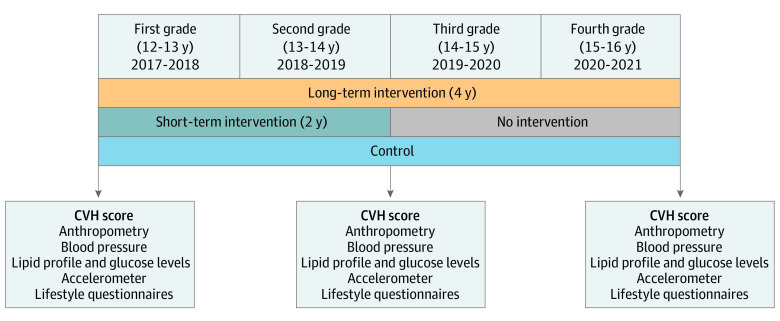
Study Design and Primary End Point of the SI! Program for Secondary Schools CVH indicates cardiovascular health.

**Figure 2.**
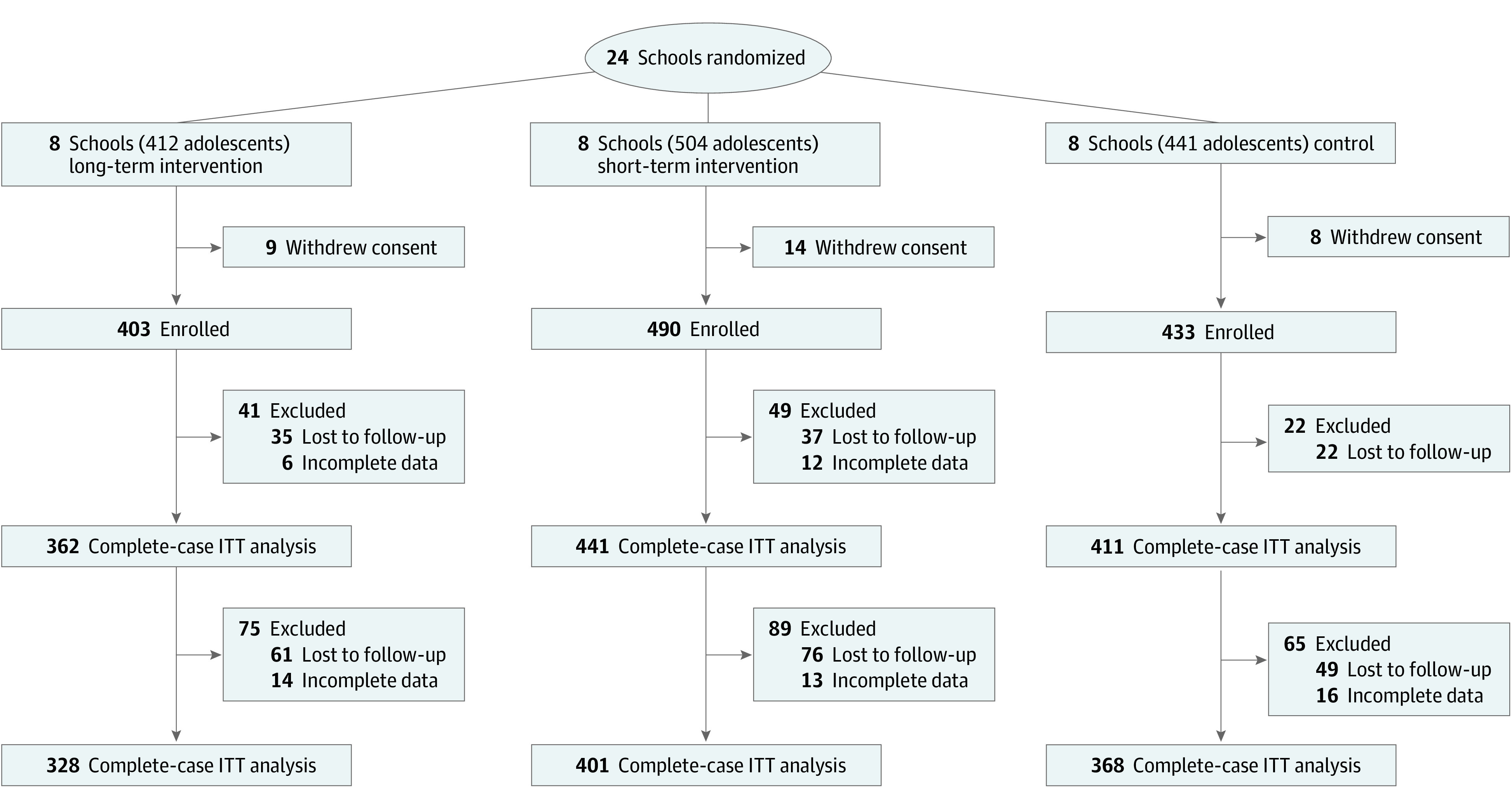
Study Flowchart First and second follow-up analyses are independent. No school discontinued the study. ITT indicates intention to treat.

The design and rationale of the SI! Program for Secondary Schools trial has been published elsewhere.^12^ Briefly, this study was designed as a cluster randomized controlled intervention to test the effect of a comprehensive lifestyle program on the CVH of adolescents aged 12 to 16 years in Spain. The trial was launched September 7, 2017, and finalized July 31, 2021. Cluster units were schools that met the following inclusion criteria: public schools located in the metropolitan areas of Barcelona or Madrid providing education from the first through the fourth secondary school grades, with 3 to 5 classes in the first grade. The education agencies of the Madrid and Catalonia regional governments invited all eligible schools to a presentation of the study. Schools that agreed to participate were randomly allocated 1:1:1 to receive a comprehensive educational program through a long-term (4-year) intervention (LTI), a short-term (2-year) intervention (STI), or the standard curriculum (control). A simple randomization scheme was used, ensuring an equal number of schools in each group (Figure 1). The allocation sequence was generated by an independent researcher who had no previous interaction with participating schools or adolescents. The study was approved by the corresponding committees for ethical research, and all participants gave their written informed consent to enroll in the study; participants did not receive financial compensation. The eligible adolescents were all students enrolled in the first grade of the secondary school at the participating schools. The study enrolled 24 secondary schools (17 in Barcelona and 7 in Madrid), corresponding to a total of 1326 adolescents (Figure 2).^12^ The reporting of the results of this trial adheres to the Consolidated Standards of Reporting Trials Extension (CONSORT Extension) reporting guideline. The trial protocol can be found in Supplement 1.

### Intervention

The SI! Program multidimensional educational intervention is based on the principles of the transtheoretical model of change^15^ as applied to the promotion of healthy behaviors among adolescents and persons in their immediate environment (families, teachers, and school environment).^16^ The SI! Program adopts a multicomponent approach based not only on the health effects of diet and PA, but also introducing emotion management focused on assertiveness, self-esteem, and other protective behavioral strategies against the use of tobacco and other harmful substances.^13^ The intervention content was organized according to 2 strategies: LTI from first grade to fourth grade (ages 12-16 years) and the STI from first grade to second grade (ages 12-14 years). The curriculum incorporated 3 teaching units per school year: (1) healthy eating, (2) PA, and (3) protective factors against smoking and substance abuse. The curriculum was designed with an age-specific motivational theme developed through individual and group activities and interactive computer mini-games. Key messages were reinforced through newsletters distributed to families and school environment recommendations distributed to school leadership teams. All teaching activities for students were delivered in the classroom by their regular teachers after specific training provided by the Foundation for Science, Health, and Education. However, the implementation of the intervention in the third and fourth grades (which affected the LTI group) was modified due to the COVID-19 pandemic, including adaptation to remote learning and the cancellation of some activities involving PA. The description of the intervention follows the Template for Intervention Description and Replication guidelines^17^ (eFigure 1, eTable 1, eTable 2, and the eMethods in Supplement 2 for additional information).

### Data Collection

Following American Heart Association recommendations, 7 health metrics were measured to determine the overall CVH of the adolescents (smoking status, body mass index, PA, diet, blood pressure, total cholesterol level, and blood glucose level).^18^ Cardiovascular health was defined at 3 time points (baseline, 2-year follow-up at the end of the STI and halfway through the LTI, and 4-year follow-up at the end of the LTI and 2 years after the end of the STI) using medical devices and/or self-report questionnaires. Adolescent participants were guided through questionnaires by a trained team of nutritionists and nurses or pharmacists, who also performed the clinical measurements during school hours according to a standardized protocol. Families (parents/caregivers) completed a survey with questions related to sociodemographic information (educational level, household income, and migrant status). No specific data on race and ethnicity were obtained.

Parental educational level was categorized according to the International Standard Classification of Education.^19^ If more than 1 individual parental/caregiver educational level was reported, the highest one was used for analysis. Information on household income was collected and classified according to the most recently published Spanish average annual household income at the time of data collection.^20^ Migrant background was assigned if 1 or more parents/caregivers were born outside Spain. Missing values (if any) for socioeconomic variables used to create subgroups were not imputed (eMethods Supplement 2 for more details on data collection).

### Definition of Health Scores

The overall CVH score for each adolescent was calculated from 7 CVH metrics based on the American Heart Association criteria of ideal CVH in children and adolescents as reference values.^18^ Each CVH metric was classified as ideal (score, 2), intermediate (score, 1), or poor (score, 0) (eTable 3 in Supplement 2). Overall scores were thus between 0 and 14 points, with a higher score indicating a better (healthier) CVH profile. The overall CVH score was also categorized as poor (overall CVH score, 0-7), intermediate (overall CVH score, 8-11), or ideal (overall CVH score, 12-14).^21^ The analysis included all adolescents with valid data for at least 5 of the 7 individual CVH metrics. For participants with 1 or 2 missing individual health metrics, the overall CVH was calculated as the mean of the remaining metrics. The numbers of participants with missing values were as follows: 1 value was missing in 8 participants (0.60%) at baseline, 9 (0.75%) at 2-year follow-up, and 5 (0.44%) at 4-year follow-up and 2 values were missing in 45 participants (3.39%) at baseline, 10 (0.84%) at 2-year follow-up, and 13 (1.14%) at 4-year follow-up.

### Outcomes and End Points

The principal outcome measure was the overall CVH score (range, 0-14). The primary end points were the between-group differences in the change from baseline at 2-year follow-up and 4-year follow-up. Secondary end points included within-group changes in overall CVH over time and between-group differences in individual CVH metrics.

### Qualitative Analysis

At the end of the trial, students and teachers belonging to the LTI and STI schools from Madrid and Barcelona were invited to participate in online focus group discussions to share their personal experiences within the SI! Program. Four focus groups were conducted with fourth-grade students (11 girls and 13 boys), and 2 were conducted with 14 teachers (11 women and 3 men). Each session lasted 60 to 90 minutes, and the number of participants per session ranged from 4 to 9. Focus groups were led by a sociologist who conducted the interviews and the subsequent discursive analysis.^22^

### Statistical Analysis

The required trial sample size was estimated as previously described.^12^ Continuous variables are presented as mean (SD), and categorical variables are presented as frequencies and percentages. Multilevel linear mixed-effects models that account for the hierarchical cluster randomized design were used to assess within- and between-group difference in overall CVH score as a continuous variable (range, 0-14 points) and for each of the 7 individual health metrics (range, 0-2 points). Similar models were built to assess the difference in the continuous variables that form the metrics of the CVH score. Stratified models were built according to socioeconomic variables of interest. Fixed effects were the corresponding baseline score (as a continuous variable) and randomization group, whereas region (Madrid or Barcelona) and schools within each region were handled as random effects. Additional models were also adjusted for gender, age, household income, and migrant status. The Kenward-Roger method for small sample correction was used in all models.

Every attempt was made to follow up all enrolled participants, irrespective of allocation or treatment withdrawal. All participants were analyzed in the groups to which they were randomized. A complete-case intention-to-treat analysis was performed as the main analysis. As a sensitivity analysis, missing data were considered at random, and an analysis was performed including all enrolled participants after multiple imputation, using multivariate normal distribution. Further details of multiple imputation procedures performed can be found in the eMethods in Supplement 2. Statistical significance was set at 2-sided *P* < .05. All analyses were performed using Stata, version 15 (Stata Corp LLC).

## Results

### Participant Flow Diagram and Baseline Characteristics

The trial randomized and enrolled 1326 adolescents (684 [51.6%] boys, 642 [48.4%] girls) at 24 schools, with a study completion rate of 86.0%. Mean (SD) participant age at recruitment was 12.5 (0.4) years. No school withdrew from the trial during the study period, and no adverse events were reported.

A total of 1324 (99.8%) adolescents completed the baseline, 1214 (91.6%) completed the 2-year follow-up (median, 16.0; IQR, 15.2-16.9 months), and 1097 (82.7%) completed the 4-year follow-up (median, 40.4; IQR, 38.9-40.9 months) primary outcome assessments. These populations constituted the case-complete intention-to-treat analysis population (Figure 2; eFigure 2 in Supplement 2).

Adolescents were mainly classified as having intermediate overall CVH (65.5%), with a mean (SD) baseline CVH score of 10.5 (1.7) points and no significant differences between randomized groups (Table 1). Baseline information for participants included in the main analysis vs those with incomplete data and lost to follow-up is presented in eTable 4 and eTable 5 in Supplement 2.

**Table 1.** Baseline Characteristics of Participants Enrolled in the SI! Program for Secondary Schools Trial^a^

Characteristic	Long-term intervention	Short-term intervention	Control
Schools			
No. of schools	8	8	8
No. of adolescents/school, mean (SD)	50.4 (10.5)	61.2 (21.8)	54.1 (14.6)
Families			
Region, No. (%)			
Barcelona	294 (73.0)	273 (55.7)	335 (77.4)
Madrid	109 (27.0)	217 (44.3)	98 (22.6)
Household income, No. (%)			
Low	156 (39.5)	150 (31.3)	130 (30.2)
Average	141 (35.7)	143 (29.9)	126 (29.2)
High	98 (24.8)	186 (38.8)	175 (40.6)
Parental educational level, No. (%)			
Low	88 (22.2)	78 (16.3)	80 (18.5)
Medium	171 (43.1)	183 (38.2)	183 (42.3)
High	138 (34.8)	218 (45.5)	170 (39.3)
Migrant background, No. (%)			
No	223 (56.3)	333 (69.5)	322 (74.5)
Yes	173 (43.7)	146 (30.5)	110 (25.5)
Adolescents			
No. of adolescents, No. (%)	403 (30.4)	490 (37.0)	433 (32.3)
Age, mean (SD), y	12.6 (0.5)	12.5 (0.4)	12.5 (0.4)
Gender, No. (%)			
Boys	210 (52.1)	250 (51.0)	224 (51.7)
Girls	193 (47.9)	240 (49.0)	209 (48.3)
Overall CVH score, mean (SD)	10.3 (1.7)	10.6 (1.5)	10.5 (1.7)
Overall CVH score categorized, No. (%)			
Poor	29 (7.2)	19 (3.9)	26 (6.0)
Intermediate	265 (65.8)	327 (66.9)	275 (63.7)
Ideal	109 (27.0)	143 (29.2)	131 (30.3)
Individual CVH metrics, mean (SD)^b^			
Smoking status	1.9 (0.5)	1.8 (0.6)	1.9 (0.5)
Body mass index	1.6 (0.7)	1.7 (0.6)	1.6 (0.7)
Physical activity	1.7 (0.5)	1.7 (0.4)	1.7 (0.4)
Diet	0.6 (0.5)	0.6 (0.5)	0.6 (0.5)
Blood pressure	1.8 (0.6)	1.7 (06)	1.7 (0.6)
Total cholesterol level	1.6 (0.7)	1.7 (0.5)	1.5 (0.7)
Blood glucose level^c^	1.2 (0.5)	1.4 (0.5)	1.4 (0.5)

Abbreviation: CVH, cardiovascular health.

^a^ The number of participants varied due to data availability.

^b^ Individual CVH metrics range from 0 to 2 points. Overall CVH score (range, 0-14 points) was categorized as poor (overall CVH, 0-7), intermediate (overall CVH, 8-11), or ideal (overall CVH, 12-14).

^c^ Although participants were instructed to fast overnight before the assessments, some of them may have had a nonfasting status at the time of measurements.

### Primary End Points: Between-Group Changes in Overall CVH Score at 2- and 4-Year Follow-Up

Mean (SD) baseline overall CVH score was 10.3 (1.7) in the LTI group, 10.6 (1.5) in the STI group, and 10.5 (1.7) points in the control group. At 2-year follow-up, the mean difference between the control and LTI groups in the change in overall CVH score was 0.44 points (95% CI, 0.01-0.87; *P* = .04); for the comparison of the control and STI groups, the difference was 0.18 points (95% CI, −0.25 to 0.61; *P* = .39) (Table 2). At 4-year follow-up, the mean difference between the control and LTI groups in the change of overall CVH was 0.12 points (95% CI, −0.19 to 0.43; *P* = .42), and the difference between the control and STI groups was 0.13 points (95% CI, −0.17 to 0.44; *P* = .38) (Table 3). Overall results were similar in an analysis of all randomized enrolled participants after multiple imputation (eTable 6 in Supplement 2). In subgroup analysis, no consistent significant interaction effects were detected (eFigure 3 and eFigure 4 in Supplement 2).

**Table 2.** Changes in the Overall CVH Score and Individual CVH Metrics at 2-Year Follow-up, Within and Between Randomization Groups

Variable	Mean difference (95% CI)						
	Within-group^a^			Between-group^b^			
	Long-term intervention	Short-term intervention	Control	Control vs long-term intervention	*P* value	Control vs short-term intervention	*P* value
2-y Follow-up							
Overall CVH score	0.13 (−0.40 to 0.66)	−0.13 (−0.65 to 0.39)	−0.31 (−0.84 to 0.22)	0.44 (0.01 to 0.87)	.04	0.18 (−0.25 to 0.61)	.39
Individual metric							
Smoking status	−0.36 (−0.61 to −0.11)	−0.42 (−0.67 to −0.17)	−0.47 (−0.73 to −0.22)	0.11 (−0.07 to 0.30)	.21	0.05 (−0.13 to 0.24)	.56
Body mass index	0.01 (−0.06 to 0.08)	0.07 (0.01 to 0.14)	0.01 (−0.05 to 0.08)	−0.00 (−0.06 to 0.05)	.86	0.06 (0.00 to 0.11)	.05
Physical activity	−0.08 (−0.14 to −0.02)	−0.09 (−0.15 to −0.04)	−0.04 (−0.10 to 0.02)	−0.04 (−0.13 to 0.05)	.38	−0.06 (−0.15 to 0.03)	.21
Diet	0.07 (0.00 to 0.13)	0.01 (−0.05 to 0.07)	−0.01 (−0.07 to 0.05)	0.08 (−0.01 to 0.17)	.09	0.02 (−0.07 to 0.12)	.59
Blood pressure	0.09 (0.03 to 0.14)	0.04 (−0.01 to 0.10)	0.01 (−0.04 to 0.07)	0.07 (−0.01 to 0.16)	.09	0.03 (−0.06 to 0.12)	.48
Total cholesterol	0.06 (−0.13 to 0.25)	0.01 (−0.18 to 0.19)	0.09 (−0.10 to 0.28)	−0.03 (−0.21 to 0.15)	.71	−0.08 (−0.26 to 0.10)	.34
Blood glucose^c^	0.32 (−0.02 to 0.66)	0.27 (−0.06 to 0.61)	0.12 (−0.22 to 0.46)	0.20 (0.03 to 0.37)	.03	0.15 (−0.02 to 0.33)	.08

Abbreviation: CVH, cardiovascular health.

^a^ Mean marginal within-group differences (change from baseline to follow-up in each group) and 95% CI were derived from linear mixed-effects models. Fixed effects were baseline CVH score and randomization group, whereas region (Madrid or Barcelona) and schools within each region were handled as random effects. The Kenward-Roger method for small sample correction was used.

^b^ Mean between-group differences (difference between groups in the change from baseline to follow-up) and 95% CI derived from linear mixed-effects models. Fixed effects were baseline CVH score and randomization group, while region (Madrid or Barcelona) and schools within each region were handled as random effects. The Kenward-Roger method for small sample correction was used.

^c^ Although participants were instructed to fast overnight before the assessments, some of them may have had a nonfasting status at the time of measurements.

**Table 3.** Changes in the Overall CVH Score and Individual CVH Metrics at 4-Year Follow-up, Within and Between Randomization Groups

Variable	Mean difference (95% CI)						
	Within-group^a^			Between-group^b^			
	Long-term intervention	Short-term intervention	Control	Control vs long-term intervention	*P* value	Control vs short-term intervention	*P* value
4-y Follow-up							
Overall CVH score	−0.35 (−1.11 to 0.40)	−0.34 (−1.09 to 0.41)	−0.47 (−1.22 to 0.28)	0.12 (−0.19 to 0.43)	.42	0.13 (−0.17 to 0.44)	.38
Individual metrics							
Smoking status	−0.66 (−0.79 to −0.52)	−0.74 (−0.87 to −0.61)	−0.79 (−0.93 to −0.66)	0.14 (−0.05 to 0.32)	.14	0.05 (−0.14 to 0.24)	.58
Body mass index	0.03 (−0.04 to 0.10)	0.09 (0.02 to 0.16)	0.06 (−0.01 to 0.13)	−0.03 (−0.10 to 0.03)	.31	0.03 (−0.04 to 0.10)	.38
Physical activity	−0.31 (−0.40 to −0.21)	−0.29 (−0.38 to −0.19)	−0.27 (−0.36 to −0.17)	−0.04 (−0.14 to 0.06)	.43	−0.02 (−0.12 to 0.08)	.69
Diet	0.04 (−0.03 to 0.11)	0.04 (−0.02 to 0.11)	0.07 (0.01 to 0.14)	−0.03 (−0.13 to 0.07)	.50	−0.03 (−0.13 to 0.07)	.55
Blood pressure	0.02 (−0.22 to 0.27)	0.02 (−0.22 to 0.26)	−0.02 (−0.26 to 0.22)	0.04 (−0.06 to 0.15)	.40	0.04 (−0.07 to 0.14)	.48
Total cholesterol	0.07 (0.01 to 0.12)	0.11 (0.06 to 0.16)	0.16 (0.11 to 0.22)	−0.10 (−0.18 to −0.02)	.02	−0.05 (−0.13 to 0.03)	.20
Blood glucose^c^	0.43 (0.23 to 0.64)	0.44 (0.24 to 0.64)	0.37 (0.17 to 0.57)	0.07 (−0.03 to 0.17)	.19	0.07 (−0.03 to 0.18)	.14

Abbreviation: CVH, cardiovascular health.

^a^ Mean marginal within-group differences (change from baseline to follow-up in each group) and 95% CI were derived from linear mixed-effects models. Fixed effects were baseline CVH score and randomization group, whereas region (Madrid or Barcelona) and schools within each region were handled as random effects. The Kenward-Roger method for small sample correction was used.

^b^ Mean between-group differences (difference between groups in the change from baseline to follow-up) and 95% CI derived from linear mixed-effects models. Fixed effects were baseline CVH score and randomization group, while region (Madrid or Barcelona) and schools within each region were handled as random effects. The Kenward-Roger method for small sample correction was used.

^c^ Although participants were instructed to fast overnight before the assessments, some of them may have had a nonfasting status at the time of measurements.

### Secondary End Points: Within-Group Changes in Overall CVH and Between-Group Changes in Individual CVH Metrics

Many within-group changes over time in overall CVH score were larger in the intervention groups; however, no statistically significant within-group differences were observed in any group at any follow-up, and most between-group differences in the change in individual CVH metrics were nonsignificant (Tables 2 and 3). Similar changes were noted using continuous data for the metrics included in the CVH score (eTable 7 and eTable 8 in Supplement 2) and after adjusting for gender, age, household income, and migrant status (eTables 9 and eTable 10 in Supplement 2).

### Qualitative Analysis of Focus Groups

The participants were asked about their personal experience within the SI! Program. In all cases, the feedback was positive despite the complex situation due to the COVID-19 pandemic that affected mostly the last 2 years of implementation in the LTI group. The main qualitative results can be found in eTable 11 in Supplement 2.

## Discussion

The SI! Program for Secondary Schools cluster randomized clinical trial enrolled a large sample of adolescents and randomized them to receive 1 of 2 interventions differing in duration and intensity (LTI vs STI) or the control. The primary results of the trial showed an overall neutral effect of the 2 tested multicomponent educational health promotion strategies on adolescents’ CVH. Although there was evidence of a marginal beneficial effect at a time point halfway through implementation in the LTI group, no such effect was noted at 4 years. To our knowledge, this is one of the largest trials to date evaluating a holistic school-based intervention for overall CVH promotion in adolescents.

### The Effect of Intervention Duration and Intensity on Health Promotion

One of the main objectives of the trial was to assess the effect of different timings and intensities of educational health promotion in adolescents. Although the curriculum of the 2 interventions was similar, the STI condensed all the content into 2 years, whereas LTI distributed that content over 4 years, thus requiring the dedication of fewer hours per school year (eTable 3 in Supplement 2). Focus groups conducted during the course of the trial and feedback received after completion revealed that teachers found the content very difficult to implement in just 2 years (eTable 11 in Supplement 2). This finding is unsurprising since educational innovation programs are usually consolidated in the third year, after teachers become familiar with the content and begin to include it effectively during the first 2 years. In addition, teachers have to pay attention to other academic and administrative tasks, so a more intense intervention increases the risk that these responsibilities might conflict with implementation.^13^

Moreover, while the marginal effects observed at 2-year follow-up were not affected by the pandemic, the results at the 4-year follow-up can only be interpreted as a surrogate of the planned intervention. The implementation of the intervention in third and fourth grades was affected due to the associated work overload, periods of self-quarantine, and burnout of teachers and students. Despite combined efforts from schools and the study team to adapt the intervention contents to the pandemic situation, adolescents only attended schools every other week during the last year of the study, and some intervention activities (those related to PA) were canceled during lockdown and in the following school year. Unquestionably, the switch to digital instruction was a major hurdle for teachers and students, and also for their families.

### Effect of the Intervention on CVH Components

The deterioration of the overall CVH score identified through the within-group changes over time (Tables 2 and 3) is unsurprising, particularly in relation to the evolution of the PA score and smoking status, since adolescence is a critical behavioral phase when PA tends to decrease and smoking often starts.^5,23,24,25^ The between-group differences in the change of overall CVH score were likely the result of the accumulation of small or nonsignificant differences in individual health metrics. For example, although between-group differences in the change of smoking status were not statistically significant, they were consistently larger in the intervention groups, and thus the intervention may have reduced the use of tobacco to some extent. The SI! Program curriculum included assertiveness, self-esteem, and socioemotional skills necessary to make healthy decisions and avoid substance use.

A significant difference in the glucose metric, with a higher score noted in the LTI group vs the other groups, was observed at 2-year follow-up; however, this difference may reflect assessment of some participants in nonfasting conditions, thus introducing a variable that may have randomly affected different groups to differing extents. In addition, although the difference was nonsignificant, a higher dietary score in the LTI group was found at 2-year follow-up, suggesting a modest improvement in dietary habits in at least some of the intervention participants. The results of focus groups with teachers and adolescents showed that participation in the trial assessments may have played a fundamental role in raising health awareness in all randomized groups (including controls), mostly regarding eating habits. In addition, the expected wide acceptance of the Mediterranean lifestyle in participating families may explain the lack of significant differences between groups.

### Health Promotion Interventions in Adolescents

There are no discernible patterns in the literature suggesting effective mechanisms for school-based health promotion. Moreover, there is a lack of multidimensional interventions, with most previous health promotion approaches in adolescents focusing on specific modifiable lifestyle factors, such as diet or PA, and considering specific related outcomes. A systematic review of meta-analyses on adolescent obesity prevention noted that most behavioral/educational interventions focused on a single component showed no statistically significant differences in weight-related outcomes, so that combined interventions seemed to represent greater benefits.^10^ However, another recent systematic review found some evidence of support for PA-only interventions and limited evidence for diet-only and combined PA and diet interventions.^26^

In contrast, the SI! Program used a multicomponent intervention to achieve a holistic approach to health promotion in school settings. The curriculum aims to increase health literacy and individual empowerment by providing students with tools to make general healthy lifestyle decisions and take action on behalf of themselves and others. Although beneficial changes in health factors such as blood pressure and total cholesterol levels are difficult to achieve over a short period, the overall CVH score was chosen ambitiously as the main trial outcome because of its clinical relevance. Nevertheless, we also observed no beneficial effects for some key modifiable lifestyle factors. Because of the categorization of the CVH components, the score might not capture smaller improvements. In any case, to achieve significant changes in the overall CVH score, the greatest changes need to occur first in the behavioral components. Results from diverse health promotion strategies in adults report that positive outcomes possibly related to health promotion interventions tend to disappear over time,^27,28^ suggesting the value of reintervention strategies. Consequently, this kind of educational program may require a more suitable primary end point and a reintervention to achieve sustained behavioral effects that may therefore result in a meaningful effect on biological parameters.

### Limitations

This trial has limitations. A major unpredicted limitation to implementation was the general lockdown due to the COVID-19 pandemic and the subsequent changes in school routines. The long duration of the trial increased the difficulty of retaining participants throughout the study. However, potential loss to follow-up and dropouts were factored into the sample size calculation, and the trial enrolled more participants than expected. Furthermore, the primary analysis was supplemented by a series of sensitivity analyses, and overall results were similar.

Regarding CVH measurements, some participants were likely assessed in nonfasting conditions, therefore affecting recorded blood glucose levels. However, an additional sensitivity analysis excluding the blood glucose metric from the overall CVH score calculations did not alter the overall direction of the results (eTable 12 in Supplement 2). In addition, adolescents were often asked by trainers and teachers to remove accelerometers for security reasons during training and competition, and therefore PA might have been underestimated in some cases.

## Conclusions

The SI! Program for Secondary Schools cluster randomized clinical trial showed an overall neutral effect on adolescents’ CVH regardless of the received school-based health promotion intervention. Although the LTI had a marginal beneficial effect at a time point halfway through implementation (2-year follow-up), the COVID-19 pandemic affected its implementation afterward, and the 4-year follow-up results might not reflect the full potential of the LTI. Cardiovascular health usually worsens with age, with adolescence being a particularly vulnerable behavioral period. Therefore, educational programs may need to include an age-tailored reintervention phase to achieve sustained behavioral effects, paying special attention to the curriculum intensity.

## Notes

Supplement 1. Trial Protocol

Supplement 2. **eMethods.** Detailed Methods **eTable 1.** Template for Intervention Description and Replication (TIDieR) Checklist **eTable 2.** Objectives and Timing: Long-term and Short-term Intervention **eTable 3.** Ideal Cardiovascular Health Metrics for Children and Adolescents as Defined by the American Heart Association and as Used in this Study **eTable 4.** Baseline Characteristics of Adolescents Included and Excluded From the Primary Analysis (Complete-Case Intention-to-Treat Analysis) at 2-Year Follow-up in the SI! Program for Secondary School Trial **eTable 5.** Baseline Characteristics of Adolescents Included and Excluded From the Primary Analysis (Complete-Case Intention-to-Treat Analysis) at 4-Year Follow-up in the SI! Program for Secondary School Trial **eTable 6.** Overall CVH Change Within and Between Intervention Groups for All Randomized Enrolled Participants **eTable 7.** Changes in the Continuous Metrics Comprising the CVH Score at 2-Year Follow-up, Within and Between Randomization Groups **eTable 8.** Changes in the Continuous Metrics Comprising the CVH Score at 4-Year Follow-up, Within and Between Randomization Groups **eTable 9.** Adjusted Changes in the Overall CVH Score and Individual CVH Metrics at 2-Year Follow-up, Within and Between Randomization Groups **eTable 10.** Adjusted Changes in the Overall CVH Score and Individual CVH Metrics at 4-Year Follow-up, Within and Between Randomization Groups **eTable 11.** Main Qualitative Results From Focus Groups on Teachers and Students **eTable 12.** Overall CVH Change Within and Between Intervention Groups Excluding the Blood Glucose Metric **eFigure 1.** Examples of Intervention Material **eFigure 2.** Extended Study Flowchart **eFigure 3.** Change in the Overall CVH Score at 2-Year Follow-up According to Sociodemographic Characteristics **eFigure 4.** Change in the Overall CVH Score at 4-Year Follow-up According to Sociodemographic Characteristics
eReferences

Supplement 3.
Data Sharing Statement
