# Supplementary material for: School-Based Cardiovascular Health Promotion in Adolescents: A Cluster Randomized Clinical Trial
Source: JAMA Cardiol. 2023 Aug 2;8(9):816–24. doi: 10.1001/jamacardio.2023.2231 (PMC10398546; doi:10.1001/jamacardio.2023.2231)
Supplement: Supplement 1. — Trial Protocol [file jamacardiol-e232231-s001.pdf]

MEMÒRIA

PROJECT TITLE: School-based educational intervention to face obesity and promote Cardiovascular health among Spanish adolescents: a cluster-randomized controlled trial.

PRESENTATION

Obesity is strongly associated with increase mortality and multiple comorbidities, mainly related to cardiovascular diseases. This problem is anticipated to be exponentially magnified in the coming years due to the alarming increase in overweight and unhealthy lifestyles in adolescents. The only way to tackle this epidemic is to implement effective preventive strategies able to positively impact on youth health-related behaviours. Current evidence suggests that conventional approaches are inadequate for promoting healthier lifestyles; therefore implementation of effective methods is desperately needed. The school is the most appropriate choice for such an intervention. However, no previous studies has analysed the long-term effects of a comprehensive educational intervention on health parameters including adiposity and metabolic profiles in a large sample of adolescents.

HYPOTHESIS

The global hypothesis is that a comprehensive educational intervention at secondary school on healthy habits will induce favourable lifestyle changes in adolescents. These changes will have a measurable positive effect on obesity/adiposity, metabolic profiles and other cardiovascular health parameters which will be carried to adulthood.

OBJECTIVES

Our main objective is to evaluate whether a comprehensive educational intervention, focused on the acquisition and maintenance of healthy behaviours, is able to improve obesity/adiposity parameters and cardiovascular health in adolescence.

The primary outcome will be the change in obesity and other health parameters from baseline to year 2 and 4, as assessed by the Ideal Cardiovascular Health score, as recommended by the American Heart Association. Secondary outcomes and objectives will include the following: the change in body mass index, waist circumference and adiposity measured by bioelectrical

impedance and dual energy X-ray absorptiometry; to evaluate the association between changes in adiposity parameters and food and nutrient intake, mainly polyphenol intake, measured by food frequency questionnaire and biochemical determination in salivary and urine; to study the relationship between carotenoids intake and anthropometric measures; to evaluate the effect on metabolism in the intervention group vs. control group through a metabolomics approach; to study the association between changes in adiposity parameters and physical activity measured by questionnaires and accelerometer; to evaluate the relationship between changes in adiposity and attitudes; to evaluate the maintenance of individual components and biological parameters of normal weight and cardiovascular health in adulthood; to compare the effectiveness of a shorter vs. longer educational intervention program; and to validate a non-invasive score of cardiovascular health status.

To achieve these objectives, a cluster randomized controlled trial involving 24 secondary schools in Catalonia and Madrid will be conducted.

METHODOLOGY

To achieve the previously detailed objectives, a cluster-randomized controlled trial involving 24 secondary schools (18 in Catalonia and 6 in Madrid) and 50-60 students per school (1200 participants) will be conducted. The full study will consist of a 1-year pilot phase followed by a 4-year randomized controlled trial. A pre-specified follow-up after the end of the trial until participants are 20 years of age is also planned.

Pilot phase

The pilot phase will take place during the academic year 2016-17. The aim is to test the feasibility of the intervention, materials, questionnaires and measurements. The results will allow us to make final modifications as needed for their proper implementation in the randomized study.

Cluster-randomized controlled trial

The cluster-randomized controlled trial will be conducted during the academic years 2017-18 to 2020-21. Participant schools will be randomized to receive either the intervention during a 4-year period time coinciding with the 4 years of secondary school (long-term intervention) or during a 2-year period time coinciding with the 1st and 2nd years of secondary school (short-

term intervention), or to receive the usual curriculum (controls). Control schools will be unaware of the specific details of the intervention.

Design of the intervention

- **Classroom intervention**

The most relevant teaching methodologies will be used in order to increase adolescents critical leading to a better self-control of their own behaviour. Each course will include 12 hours of education in the classroom covering all healthy behaviours (healthy eating, physical activity and avoiding substance abuse), based on different motivational axis and common methodological features but different content. The educational resources for the intervention will be introduced through an interactive game (to be downloaded on a pc, mobile phone or Tablet). The game consists of facing a challenge each course in small groups. The schools allocated to the short 2-year intervention will receive the educational program during the first two years of secondary school, coinciding with the age of 12-14 years, the critical period for the initiation of unhealthy behaviours. If we can demonstrate similar beneficial effects with the longer and the shorter intervention vs. the control group, the implementation could be easier regarding eventual future expansion and dissemination.

- **Intervention in the family setting**

The strategies that will be implemented are the following: allowing access to downloadable information and providing official links about the project; promoting healthy leisure time activities together with their children (e.g. cooking recipes, family excursions); modelling through audiovisual content about skills in life; promoting family involvement by creating groups of parents boosting health activities.

- **Intervention at school**

The intervention at school will be carried out by providing a document with recommendations to be implemented in certain domains such as increasing physical activity during recess, improving dietary choices in the cantines, and conflict solving at the school. Each school will have a health team consisting of adolescent’s representatives, management team, teachers, families and health coordinator, and will discuss about yearly goals. A health week will take place during each academic year.

- **Intervention on teachers**

One teacher per school will be trained to assume the responsibility of promoting cardiovascular health in the school setting. This health coordinator will be the role model for the rest of the teachers participating in the program. The health team and the health coordinator will work together on the program activities and workshops related to the project. All contents will be conceived taking into account the participation of teachers, adolescents and families.

MEASUREMENTS

Once the randomized study begins, measurements will take place at the beginning of the 1st year (baseline), at the end of the 2nd year (peak effect of the short-term intervention and intermediate evaluation of the long-term one of primary and secondary endpoints), and at the end of the 4rd year of secondary school (final evaluation of primary and secondary endpoints, including the peak effect of the long-term intervention). A pre-specified follow-up when the participants are 20 years of age will be performed to evaluate the maintenance of obesity/adiposity parameters and cardiovascular health in adulthood. All the measurements and questionnaires to be performed at baseline, and end of 2nd and 4rd years are briefly detailed below.

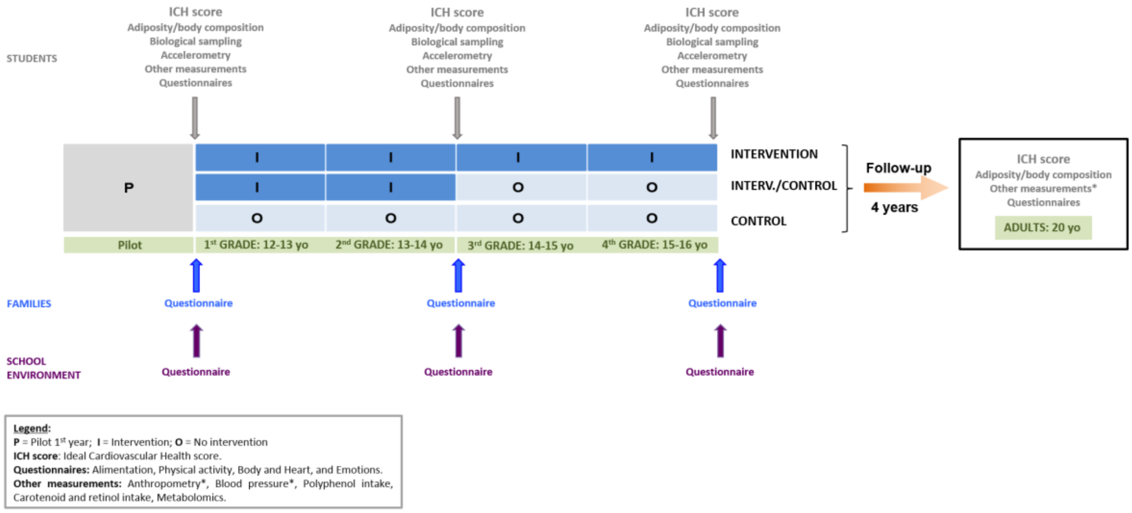

- ### Anthropometry and body composition

Weight (SECA scale Model 803), height (SECA scale Model 213), waist circumference (HOLTAIN measuring tape), and fat percentage (OMRON bioelectrical impedance) will be assessed in all participants. From these measurements, ratios such as body mass index, fat mass index or waist-height index will be calculated.

In addition, total body fat, regional fat (android, gynoid and visceral) and lean mass will be measured by dual energy X-ray absorptiometry (DEXA) using a Whole-Body Scanner GE Lunar iDXA (GE Healthcare) in a subsample of 50% participants. DEXA method to measure percentage fat mass is more precise than bioelectrical impedance analysis (BIA). The regions of interest for regional body composition will be defined using the software provided by the manufacturer.

- **Physical activity**

Actigraph wGT3X-BT accelerometers that allows the registration of the amount and intensity of physical activity will be used. To complete this information a diary with questions about the type of physical activity that have performed during the days they worn the accelerometer, namely 7 days per evaluation time-point. A validated questionnaire will be also used to assess attitudes about physical activity. In addition, we will measure sedentary habits from leisure time spent with the computer/console/TV /mobile.

- **Diet**

To assess changes in diet and adherence to the Mediterranean diet in adolescents we will use a validated 137-item Food Frequency Questionnaire (FFQ). Additional validated shorter questionnaires will be used to assess attitudes about food.

- **Blood pressure**

Peripheral arterial blood pressure will be measured by using an OMRON M6 device following standard protocols used in previous studies.

- **Biochemistry**

Blood glucose, total cholesterol, HDL- and LDL-cholesterol, and triglyceride levels will be measured by using the CholestechLDX® device, via capillary blood sampling with a lancet. An e-mail will be sent to families with the analytical results obtained.

- **Dietary polyphenol intake**

We will estimate dietary polyphenol intake from a validated 137-item FFQ and data from the polyphenol content in foods, which will be obtained from the Phenol-explorer database (www.phenol-explorer.eu). With this methodology, we obtain not only an estimation of total polyphenol intake by each individual, but also the estimation of different polyphenol classes.

- **Dietary polyphenol in urine**

For the determination of phenolics and their metabolites in human urine, we will use solid-phase extraction and ultra-high performance liquid chromatography coupled to mass spectrometry.

- **Dietary carotenoids and retinol intake**

Carotenoids and retinol consumption will be also estimate from the FFQ. Total energy and micronutrient intake will be obtained using the Food Processor Nutrition and Fitness Software.

- **Metabolomics**

Urine and salivary samples will be collected to perform a metabolomics analysis in order to evaluate metabolic changes due to educational intervention on dietary and physical activity habits. Liquid chromatography coupled to quadrupole time-of-flight mass spectrometry (HPLC-q-TOF) will be used for such analysis which is able to measure thousands of metabolites simultaneously from only minimal amounts of sample. Data mining analysis will be performed using XCMS-R package. The preprocessed data obtained by XCMS in negative and positive ionization will be separately exported to SIMCA 13.0.3 software (Umetrics) to perform unsupervised and supervised multivariate analysis to detect metabolic changes among groups.

- **Substances abuse**

The attitudes towards abuse substances will be assessed by a validated questionnaire (Lima-Serrano et al., 2013). An additional questionnaire will be used to assess knowledge about smoking, smoking status itself, and in its immediate environment .

- **Emotions**

A questionnaire to assess the perception of the self-image to obtain a pattern of individual acceptance or not with his/her figure and an analysis of the canons of beauty in this age group. Self-esteem, emotional eating, self-efficacy and mood will be assessed using validated questionnaires.

- **BEWAT score**

It consists of the assessment of the following: Blood pressure, Exercise, Weight, Alimentation (fruit and vegetables) and Tobacco consumption. This simple and non-invasive score (score from 0 to 15 points) will be validated against the ICH score, and will serve for future studies in adolescents.

- **Families and school environment**

A questionnaire which includes questions related to sociodemographic and lifestyle aspects (smoking, diet, physical activity, leisure time, body mass index and blood pressure) at the family level will be filled in by parents/caregivers. For the evaluation of school environment, a survey will be fill in by the Principal of each school with questions related to recommendations made from the Project coordinators to the school in terms of types of products for sailing in cafeteria or vending machines, promotion of physical activity at recesses, and resolution of conflicts.

ENDPOINTS

The main hypothesis is that individuals belonging to the intervention groups (either to the shorter or longer one) experience and improvement in obesity and other health parameters as assessed by the ICH score when compared to the control group. The primary outcome will be the 2- and 4-year change from baseline of the ICH score.

Secondary outcomes will include the change or evaluation of associations from baseline to year 2 and 4 of the following parameters: body mass index, waist circumference and body fat percentage and composition; physical activity; food and nutrient intake; polyphenol intake; carotenoids intake; metabolites; and emotions. Other secondary outcomes include the evaluation of the maintenance of individual components and biological parameters of normal weight and cardiovascular health in adulthood (at 20 years old); the comparison between the shorter vs. longer educational program; and the validation of the BEWAT score.

CONDUCT OF THE TRIAL

To join the study it will be an essential requirement that parents or guardians of students who will be part of the study, and teachers, from both interventional or control schools, sign an informed consent.

Once the trial has started, the coordinator will follow up the subsequent communication with Ethics Committees and Competent Authorities throughout the duration of the trial. The study will be registered in clinicaltrials.gov according to standard procedures.
At all times the confidentiality of all data provided by participants will be ensured. The information will be collected, storage and managed by using an on-line application; and treated according to the requirements of Ley Orgánica 15/1999 (LOPD) and Real Decreto 1720/2007.

HOJA INFORMATIVA Y CONSENTIMIENTO INFORMADO

A) Estudio Piloto Madrid

B) Estudio Piloto Cataluña

C) Estudio Aleatorizado: Grupo Intervención Madrid

D) Estudio Aleatorizado: Grupo Intervención Cataluña

E) Estudio Aleatorizado: Grupo Control Madrid

F) Estudio Aleatorizado: Grupo Control Cataluña

Madrid, 10 de enero del 2017

Estimado/a alumno/a,

Tu instituto ha sido seleccionado para participar en el estudio piloto del Programa SI! de promoción de la salud cardiovascular en adolescentes, llevado a cabo por la Fundación SHE, la Fundación Centro Nacional de Investigaciones Cardiovasculares Carlos III y la Universidad de Barcelona.

Este programa se va a realizar en 4 centros de educación secundaria de la Comunidad de Madrid y en Cataluña. Como tu instituto ha sido elegido, tienes la oportunidad de participar como voluntario en este programa.

¿Qué vamos a hacer?

Tu estilo de vida (alimentación, actividad física, sedentarismo, etc) durante la adolescencia influirá en tu salud cardiovascular cuando seas adulto. Por ello, queremos **conocer la salud cardiovascular y los estilos de vida** de los adolescentes para lo cual se va a realizar una evaluación a través de instrumentos de medida validados que permita conocer la salud cardiovascular de los adolescentes mediante una serie de pruebas:

- Cuestionarios: cuestionario de alimentación, cuestionario de conocimientos, actitudes y hábitos acerca de los estilos de vida, y un cuestionario para tus padres.

- Medición de: peso, altura, circunferencia de cintura, porcentaje de grasa, presión arterial, actividad física mediante acelerómetros (pequeño dispositivo que se lleva en la cintura), parámetros bioquímicos (colesterol, glucosa, triglicéridos) mediante una gota de sangre obtenida de un dedo y medición de compuestos de la dieta a través de una muestra de orina y saliva.

Además, durante el presente curso escolar, recibiréis dentro del horario escolar una serie de contenidos dirigidos a mejorar vuestros conocimientos y actitudes hacia los estilos de vida saludables.

Una vez acabadas las pruebas, recibirás un informe con los resultados de los análisis de sangre y de las mediciones corporales, para conocer tu estado general de salud. Las muestras que obtengamos no se usarán para ningún otro fin, y serán destruidas una vez realizados los análisis. Tampoco usaremos tus muestras para realizar análisis genéticos de ningún tipo.

Tu participación en el estudio es voluntaria y gratuita. Si deseas participar, necesitamos tu firma y la de tus padres. Si en algún momento deseas retirarte del estudio, puedes hacerlo en cualquier momento y sin dar explicaciones de ningún tipo.

Toda la información recogida en este estudio será estrictamente confidencial. Eso significa que nadie podrá relacionar tu nombre con los resultados obtenidos. En todo el proceso se seguirá la Ley de Protección de Datos (Ley orgánica 15/1999 de 13 de diciembre) y otras leyes vigentes aplicables.

Con tu ayuda podremos mejorar la salud cardiovascular de la población española.

¡MUCHAS GRACIAS POR TU COLABORACIÓN!

Este proyecto ha sido aprobado por el Comité de Ética del Instituto de Salud Carlos III, la Fundació Unió Catalana d'Hospitals y la Comissió de Bioètica de la Universitat de Barcelona

Programa de Salud Integral

Madrid, 10 de enero del 2017

Estimados padres y madres,

El instituto de su hijo/a ha sido seleccionado para participar en el estudio piloto del Programa SI! de promoción de la salud cardiovascular en adolescentes, llevado a cabo por la Fundación SHE, la Fundación Centro Nacional de Investigaciones Cardiovasculares Carlos III y la Universidad de Barcelona.

Este programa se llevará a cabo **durante el presente curso escolar (2016/2017)** en 4 centros de educación secundaria situados en la Comunidad de Madrid y en Cataluña. Ustedes tienen por tanto la oportunidad de participar como voluntarios en este programa.

Objetivos del programa

La adolescencia es un período crítico durante el cual se establecen y refuerzan estilos de vida (alimentación, actividad física, sedentarismo, etc) que condicionarán la salud cardiovascular en la etapa adulta. Por esta razón el objetivo de esta iniciativa es **valorar y mejorar la salud cardiovascular y los estilos de vida** de los adolescentes para lo cual se va a realizar una evaluación a través de instrumentos de medida validados que permita conocer la salud cardiovascular de los adolescentes. Las pruebas a realizar incluyen:

- Cuestionario de alimentación (cuestionario de frecuencia de consumo de alimentos).
- Cuestionario general acerca de conocimientos, actitudes y hábitos acerca de los estilos de vida.
- Mediciones antropométricas a los adolescentes: peso, altura, circunferencia de cintura y porcentaje de grasa.
- Uso de acelerómetros para el registro objetivo de la actividad física (pequeño dispositivo que se lleva en la cintura).
- Medición de la presión arterial.
- Medición de parámetros bioquímicos (colesterol total, HDL, LDL, triglicéridos y glucosa) mediante la extracción de una gota de sangre capilar.
- Determinación de compuestos dietéticos (polifenoles y metabolitos) en una muestra de orina y saliva.
- Cuestionario general dirigido a padres y madres acerca de aspectos sociodemográficos y estilos de vida (tabaquismo, dieta, actividad física, etc).

Además, los alumnos se beneficiarán de una intervención educativa llevada a cabo dentro del programa curricular del instituto, con la ayuda de los profesores del centro, y que ha sido diseñada por el equipo pedagógico de la Fundación SHE. Dicha intervención educativa beneficiará a su hijo/a ayudando a aumentar su conocimiento acerca de la salud cardiovascular, los estilos de vida saludables y la prevención de hábitos nocivos, con un impacto positivo esperado en su perfil de salud cardiovascular.

Las familias recibirán un correo electrónico con un enlace para cumplimentar el cuestionario dirigido a padres y madres. Una vez finalizada la recogida de datos, se hará llegar a las familias un informe con los resultados principales derivados de los análisis bioquímicos, antropométricos y de presión arterial relativos al estado de salud de sus hijos. Todas las muestras serán analizadas bajo los controles de calidad pertinentes. Las muestras servirán para valorar el estado de salud general de los participantes. Las muestras sobrantes no se usarán en ninguna otra investigación y serán destruidas. No se realizarán análisis genéticos a partir de las muestras obtenidas.

La participación en el estudio es voluntaria y gratuita:

Nos gustaría aclarar que tanto la participación de su hijo/a como la suya son voluntarias y gratuitas. **Usted, su hijo/a y/o su familia es libre de retirarse del estudio en cualquier momento y sin dar explicaciones**.

Riesgos de participar en el proyecto:

La máxima molestia que puede percibir es un ligero pinchazo en el pulpejo del dedo al realizar una punción para extracción de una gota de sangre capilar.

Confidencialidad

Este proyecto ha sido aprobado por el Comité de Ética del Instituto de Salud Carlos III, la Fundació Unió Catalana d'Hospitals y la Comissió de Bioètica de la Universitat de Barcelona y cuenta con la colaboración de la Fundación SHE, la Fundación Centro Nacional de Investigaciones Cardiovasculares Carlos III y la Universidad de Barcelona.

Toda la información recogida en este estudio será estrictamente confidencial. Toda la información será tratada de forma anónima mediante su codificación. Los nombres y direcciones o vías de contacto estarán completamente separados de los datos obtenidos en el estudio, bajo llave. En todo el proceso se seguirá la Ley de Protección de Datos (Ley orgánica 15/1999 de 13 de diciembre) y otras leyes vigentes aplicables.

Los resultados anónimos derivados del estudio serán de gran ayuda para los responsables de elaborar las políticas de salud pública en el futuro, con el objetivo de mejorar la salud cardiovascular de la población española.

MUCHAS GRACIAS POR SU ATENCION Y COLABORACIÓN

Detalles de contacto

Para cualquier consulta puede dirigirse a nuestro equipo a través del **correo electrónico** estudio@fundacionshe.org o en el **teléfono** 93 218 54 44.

HOJA DE CONSENTIMIENTO INFORMADO-PROGRAMA SI! SECUNDARIA

TÍTULO DEL PROYECTO: Estudio Piloto del proyecto "Intervención educativa en escolares para hacer frente a la obesidad y promover la salud cardiovascular en adolescentes españoles".

Centros participantes: Campus de la Alimentación de Torribera. Departamento de Nutrición, Bromatología y Gastronomía de la Facultad de Farmacia (Universidad de Barcelona); Centro Nacional de Investigaciones Cardiovasculares Carlos III (CNIC); Foundation for Science Health and Education (Fundación SHE)

Investigadora principal: Dra. Rosa Mª Lamuela

Yo (nombre y apellidos) ……

autorizo a mi hija/o (nombre y apellidos) ……………………………………………………………..........................del

instituto…………………………………………………………………………………………………….……a participar en el estudio.

- He leído la hoja de información que acompaña a este consentimiento.
- He podido hacer preguntas sobre el estudio.
- He recibido suficiente información sobre el estudio.
- Se me ha informado de que los datos personales recogidos en el presente estudio se tratarán confidencialmente de acuerdo con la Ley Orgánica 15/1999 de Protección de Datos Personales, y su normativa de desarrollo.
- Se me ha informado de que los datos obtenidos sólo se utilizarán para los fines específicos del estudio.

Comprendo que la participación de mi hijo/a y la de su familia es voluntaria. Comprendo que tanto mi hijo/a como su familia pueden retirarse del estudio cuando quieran y sin tener que dar explicaciones. Además, se me ha facilitado un contacto con los investigadores del estudio para solucionar cualquier duda que pudiera surgir al respecto.

Damos libremente nuestra conformidad para participar en el estudio:

Correo electrónico: ………………………………………………………. Teléfono: ………………………../……………………………

___________________	________________________	_______________________
(Fecha)	(Firma del adolescente)	(Firma de la madre, padre o tutor legal)*

_____________________	_____________________________
(Fecha)	(Firma del investigador)

** Si la tutela es compartida, deberán firmar ambos progenitores, (en el caso de que solo firme uno, debe haber una delegación por escrito del que no está presente al que sí lo está).*

Copia para el padre y/o madre/tutor.

Este proyecto ha sido aprobado por el Comité de Ética del Instituto de Salud Carlos III, la Fundació Unió Catalana d'Hospitals y la Comissió de Bioètica de la Universitat de Barcelona

Foundation for Science, Health and Education — cnic Fundación Centro Nacional de Investigaciones Cardiovasculares Carlos III — Universitat de Barcelona

Sí! Programa de Salud Integral

HOJA DE CONSENTIMIENTO INFORMADO-PROGRAMA SI! SECUNDARIA

TÍTULO DEL PROYECTO: Estudio Piloto del proyecto "Intervención educativa en escolares para hacer frente a la obesidad y promover la salud cardiovascular en adolescentes españoles".

Centros participantes: Campus de la Alimentación de Torribera. Departamento de Nutrición, Bromatología y Gastronomía de la Facultad de Farmacia (Universidad de Barcelona); Centro Nacional de Investigaciones Cardiovasculares Carlos III (CNIC); Foundation for Science Health and Education (Fundación SHE)

Investigadora principal: Dra. Rosa Mª Lamuela

Yo (nombre y apellidos) ……

autorizo a mi hija/o (nombre y apellidos) ………………………………………………….………........................del

instituto……a participar en el estudio.

- He leído la hoja de información que acompaña a este consentimiento.
- He podido hacer preguntas sobre el estudio.
- He recibido suficiente información sobre el estudio.
- Se me ha informado de que los datos personales recogidos en el presente estudio se tratarán confidencialmente de acuerdo con la Ley Orgánica 15/1999 de Protección de Datos Personales, y su normativa de desarrollo.
- Se me ha informado de que los datos obtenidos sólo se utilizarán para los fines específicos del estudio.

Comprendo que la participación de mi hijo/a y la de su familia es voluntaria. Comprendo que tanto mi hijo/a como su familia pueden retirarse del estudio cuando quieran y sin tener que dar explicaciones. Además, se me ha facilitado un contacto con los investigadores del estudio para solucionar cualquier duda que pudiera surgir al respecto.

Damos libremente nuestra conformidad para participar en el estudio:

Correo electrónico: ……………………………………………………… Teléfono: ………………………../……………………………

___________________	________________________	_______________________
(Fecha)	(Firma del adolescente)	(Firma de la madre, padre o tutor legal)*

_____________________	_____________________________
(Fecha)	(Firma del investigador)

** Si la tutela es compartida, deberán firmar ambos progenitores, (en el caso de que solo firme uno, debe haber una delegación por escrito del que no está presente al que sí lo está).*

Copia para el investigador principal.

.

Este proyecto ha sido aprobado por el Comité de Ética del Instituto de Salud Carlos III, la Fundació Unió Catalana d'Hospitals y la Comissió de Bioètica de la Universitat de Barcelona

SHE Foundation for Science, Health and Education · cnic Fundación Centro Nacional de Investigaciones Cardiovasculares Carlos III · Universitat de Barcelona

Barcelona, 10 de gener de 2017

Benvolgut/uda alumne/a,

El teu institut ha estat seleccionat per participar en l'estudi pilot del Programa SI! de promoció de la salut cardiovascular en adolescents, que duen a terme la Fundació SHE, la Fundació Centre Nacional d'Investigacions Cardiovasculars Carlos III i la Universitat de Barcelona.

Aquest programa s'implementarà a 4 centres d'educació secundària de la Comunitat de Madrid i a Catalunya. Com que el teu institut ha estat seleccionat, tens l'oportunitat de participar com a voluntari d'aquest programa.

Què farem?

El teu estil de vida (alimentació, activitat física, sedentarisme...) durant l'adolescència influirà en la teva salut cardiovascular quan siguis adult. Per això, volem **conèixer la salut cardiovascular i els estils de vida** dels adolescents. Amb aquest fi es realitzarà una avaluació amb instruments de mesura validats que permeti conèixer la salut cardiovascular dels adolescents mitjançant una sèrie de proves:

- Qüestionaris: qüestionari d'alimentació, de coneixements, d'actituds i d'hàbits sobre els estils de vida, i un darrer qüestionari per als teus pares.

- Mesurament de: pes, estatura, circumferència de cintura, percentatge de greix, pressió arterial, activitat física mitjançant acceleròmetres (petit dispositiu que es duu a la cintura), paràmetres bioquímics (colesterol, glucosa, triglicèrids) mitjançant una gota de sang extreta d'un dit i mesurament de components de la dieta mitjançant una mostra d'orina i saliva.

- Anàlisi de la composició corporal a partir d'una densitometria òssia (DEXA). L'acceptació de participar en aquest estudi no obliga a realitzar aquesta prova, fer-la és opcional.

A més, durant el present curs, rebreu dins de l'horari escolar una sèrie de continguts dirigits a millorar els vostres coneixements i actituds envers els estils de vida saludables.

Un cop enllestides les proves, rebràs un informe amb els resultats de les anàlisis de sang, dels mesuraments corporals i de l'anàlisi de la composició corporal a partir del DEXA (si s'ha fet), per tal de conèixer el teu estat general de salut. Les mostres que obtinguem no es faran servir amb cap altra finalitat i seran destruïdes un cop realitzades les anàlisis. Tampoc no farem servir les teves mostres per dur a terme anàlisis genètiques de cap mena.

La teva participació en l'estudi és voluntària i gratuïta. Si desitges participar-hi, necessitarem la teva signatura i la dels teus pares. Si en algun moment desitges abandonar l'estudi, pots fer-ho quan vulguis i sense donar cap tipus d'explicacions.

Tota la informació recollida en aquest estudi serà tractada de forma estrictament confidencial. Això vol dir que ningú no podrà relacionar el teu nom amb els resultats obtinguts. Durant tot el procés, es respectarà la Llei de protecció de dades (Llei Orgànica 15/1999 de 13 de desembre) i altres lleis vigents aplicables.

Amb la teva ajuda podrem millorar la salut cardiovascular de la població espanyola.

MOLTES MERCÈS PER LA TEVA COL.LABORACIÓ!

Barcelona, 10 de gener de 2017

Benvolguts pares i mares,

L'institut del seu fill/a ha estat seleccionat per participar en l'estudi pilot del Programa SI! de promoció de la salut cardiovascular en adolescents, que duen a terme la Fundació SHE, la Fundació Centre Nacional d'Investigacions Cardiovasculars Carlos III i la Universitat de Barcelona.

Aquest programa s'implantarà **durant el present curs escolar (2016/2017)** a 4 centres d'educació secundària situats a la Comunitat de Madrid i a Catalunya. Vostès tenen l'oportunitat de participar com a voluntaris d'aquest programa.

Objectius del programa

L'adolescència és un període crític durant el qual s'estableixen i reforcen estils de vida (alimentació, activitat física, sedentarisme, etc.) que condicionaran la salut cardiovascular durant l'etapa adulta. Per aquest motiu, l'objectiu d'aquesta iniciativa és **valorar i millorar la salut cardiovascular i els estils de vida** dels adolescents. Amb aquest fi es durà a terme una avaluació, mitjançant instruments de mesurament validats, que permeti conèixer la salut cardiovascular dels adolescents. Les proves que farem inclouen:

- Qüestionari d'alimentació (qüestionari de freqüència de consum d'aliments).
- Qüestionari general sobre coneixements, actituds i hàbits sobre els estils de vida.
- Mesuraments antropomètrics als adolescents: pes, estatura, circumferència de cintura i percentatge de greix.
- Ús d'acceleròmetres pel registre objectiu de l'activitat física (petit dispositiu que es porta a la cintura).
- Mesurament de la pressió arterial.
- Mesurament de paràmetres bioquímics (colesterol total, HDL, LDL, triglicèrids i glucosa) mitjançant l'extracció d'una gota de sang capil·lar.
- Determinació de compostos dietètics (polifenols i metabòlits) en una mostra d'orina i saliva.
- Anàlisi de la composició corporal mitjançant una densitometria òssia (DEXA). Aquesta prova serà de caràcter opcional.
- Qüestionari general dirigit a pares i mares sobre aspectes sociodemogràfics i estils de vida (tabaquisme, dieta, activitat física, etc.).

A més a més, els alumnes es beneficiaran d'una intervenció educativa realitzada dins del programa curricular de l'institut, amb l'ajut dels professors del centre, i que ha estat dissenyada per l'equip pedagògic la Fundació SHE. Aquesta intervenció educativa beneficiarà el/la seu/va fill/a, ampliant el seu coneixement sobre la salut cardiovascular, els estils de vida saludables i la prevenció d'hàbits nocius, amb un impacte positiu esperat en el seu perfil de salut cardiovascular.

Les famílies rebran un correu electrònic amb un enllaç per poder emplenar el qüestionari dirigit a pares i mares. Un cop finalitzada la recollida de dades, es farà arribar a les famílies un informe amb els principals resultats derivats de les anàlisis bioquímiques, antropomètriques, de pressió arterial i de composició corporal obtinguts del DEXA (si s'ha fet) relatius a l'estat de salut del seus fills. Totes les mostres seran analitzades sota els controls de qualitat pertinents. Les mostres serviran per valorar l'estat de salut general dels participants. Les mostres sobrants no es faran servir a cap altre treball de recerca i seran destruïdes. No es farà cap anàlisi genètica a partir de les mostres obtingudes.

La participació en l'estudi és voluntària i gratuïta:

Ens agradaria deixar clar que tant la participació del/de la seu/va fill/a com la seva són voluntàries i gratuïtes. **Vostè, el/la seu/va fill/a i/o la seva família són lliures d'abandonar l'estudi en qualsevol moment i sense donar explicacions**.

Riscos de participar en el projecte:

La realització de la prova de DEXA suposa una dosi de radiació mínima (gairebé negligible), similar a la rebuda en un viatge d'avió d'una hora de durada o un dia de radiació ambiental natural. La màxima molèstia que pot percebre és una petita punxada al palpís del dit en punxar-li per extraure'n una gota de sang capil·lar.

Confidencialitat

Aquest projecte ha estat aprovat pel Comitè d'Ètica de l'Institut de Salut Carlos III, la Fundació Unió Catalana d'Hospitals i la Comissió de Bioètica de la Universitat de Barcelona i compta amb la col·laboració de la Fundació SHE, la Fundació Centre Nacional d'Investigacions Cardiovasculars Carlos III i la Universitat de Barcelona.

Tota la informació recollida en aquest estudi serà estrictament confidencial. Tota la informació serà tractada de forma anònima mitjançant la seva codificació. Els noms i adreces o altres dades de contacte estaran completament separades de les dades obtingudes a l'estudi, sota clau. Durant tot el procés se seguirà la Llei de protecció de dades (Llei orgànica 15/1999 de 13 de desembre) i altres lleis vigents aplicables.

Els resultats anònims derivats de l'estudi seran de gran ajuda per als responsables d'elaborar les polítiques de salut pública en el futur, amb l'objectiu de millorar la salut cardiovascular de la població espanyola.

MOLTES MERCÈS PER LA SEVA ATENCIÓ I COL.LABORACIÓ.

Detalls de contacte

Per a qualsevol consulta pot comunicar-se amb el nostre equip mitjançant el **correu electrònic** estudio@fundacionshe.org o bé trucant al **telèfon** 93 218 54 44.

FULL DE CONSENTIMENT INFORMAT-PROGRAMA SI! SECUNDÀRIA

TÍTOL DEL PROJECTE: Estudi pilot del projecte "Intervenció educativa en escolars per fer front a l'obesitat i promoure la salut cardiovascular en adolescents espanyols".

Centres participants: Campus de l'Alimentació de Torribera. Departament de Nutrició, Bromatologia i Gastronomia de la Facultat de Farmàcia (Universitat de Barcelona); Centre Nacional d'Investigacions Cardiovasculars Carlos III (CNIC); Fundació SHE (Foundation for Science Health and Education).

Investigadora principal: Dra. Rosa M. Lamuela

Jo (nom i cognoms) ………

autoritzo el/la meu/va fill/a (nom i cognoms) ……………………………………………………………………………………de

l'institut……………………………………………………………………………………………………… a participar en l'estudi.

- He llegit el full informatiu que acompanya aquest consentiment.
- He pogut fer preguntes sobre l'estudi.
- He rebut suficient informació sobre l'estudi.
- He estat informat/ada que les dades personals recollides en aquest estudi es tractaran de forma estrictament confidencial, segons la Llei orgànica 15/1999 de Protecció de dades personals i la seva normativa de desenvolupament.
- He estat informat/ada que les dades obtingudes només es faran servir per a la finalitat específica d'aquest estudi.

Comprenc que la participació del/de la meu/va fill/a i de la seva família és voluntària. Comprenc que tant el/la meu/va fill/a com la seva família poden abandonar l'estudi quan vulguin sense haver de donar explicacions. A més a més, m'han facilitat les dades de contacte dels investigadors de l'estudi per tal de poder solucionar qualsevol dubte que pogués sorgir referent a aquest estudi.

Donem lliurement la nostra conformitat per participar en l'estudi:

☐ En la seva totalitat ☐ Sense densitometria òssia (DEXA)

Correu electrònic: ………………………………………………………… Telèfon: ………………………./……………………………

___________________	________________________	_______________________
(Data)	(Signatura de l'adolescent)	(Signatura de la mare, pare o tutor legal)*

_____________________	_____________________________
(Data)	(Signatura de l'investigador)

** Si la tutela és compartida, hauran de signar ambdós progenitors (si només signés un, cal adjuntar una delegació escrita del progenitor absent al present).*

Còpia per al pare, mare o tutor.

Aquest projecte ha estat aprovat pel Comitè d'Ètica de l'Instituto de Salud Carlos III, la Fundació Unió Catalana d'Hospitals i la Comissió de Bioètica de la Universitat de Barcelona.

Programa de Salut Integral

FULL DE CONSENTIMENT INFORMAT-PROGRAMA SI! SECUNDÀRIA

TÍTOL DEL PROJECTE: Estudi pilot del projecte "Intervenció educativa en escolars per fer front a l'obesitat i promoure la salut cardiovascular en adolescents espanyols".

Centres participants: Campus de l'Alimentació de Torribera. Departament de Nutrició, Bromatologia i Gastronomia de la Facultat de Farmàcia (Universitat de Barcelona); Centre Nacional d'Investigacions Cardiovasculars Carlos III (CNIC); Fundació SHE (Foundation for Science Health and Education).

Investigadora principal: Dra. Rosa M. Lamuela

Jo (nom i cognoms) ……..

autoritzo el/la meu/va fill/a (nom i cognoms) ……………………………………………..………………..........................de

l'institut……………………………...……….....…. a participar en l'estudi.

- He llegit el full informatiu que acompanya aquest consentiment.
- He pogut fer preguntes sobre l'estudi.
- He rebut suficient informació sobre l'estudi.
- He estat informat/ada que les dades personals recollides en aquest estudi es tractaran de forma estrictament confidencial, segons la Llei orgànica 15/1999 de Protecció de dades personals i la seva normativa de desenvolupament.
- He estat informat/ada que les dades obtingudes només es faran servir per a la finalitat específica d'aquest estudi.

Comprenc que la participació del/de la meu/va fill/a i de la seva família és voluntària. Comprenc que tant el/la meu/va fill/a com la seva família poden abandonar l'estudi quan vulguin sense haver de donar explicacions. A més a més, m'han facilitat les dades de contacte dels investigadors de l'estudi per tal de poder solucionar qualsevol dubte que pogués sorgir referent a aquest estudi.

Donem lliurement la nostra conformitat per participar en l'estudi:

☐ En la seva totalitat ☐ Sense densitometria òssia (DEXA)

Correu electrònic: ………………………………………………………… Telèfon: ………………………../……………………………

___________________	________________________	_______________________
(Data)	(Signatura de l'adolescent)	(Signatura de la mare, pare o tutor legal)*

_____________________	_____________________________
(Data)	(Signatura de l'investigador)

** Si la tutela és compartida, hauran de signar ambdós progenitors (si només signés un, cal adjuntar una delegació escrita del progenitor absent al present).*

Còpia per a l'investigador principal

Aquest projecte ha estat aprovat pel Comitè d'Ètica de l'Instituto de Salud Carlos III, la Fundació Unió Catalana d'Hospitals i la Comissió de Bioètica de la Universitat de Barcelona.

SHE Foundation for Science, Health and Education · cnic Fundación Centro Nacional de Investigaciones Cardiovasculares Carlos III · UNIVERSITAT DE BARCELONA

Madrid, 10 de enero del 2017

Estimado/a alumno/a,

Tu instituto ha sido seleccionado para participar en el Programa SI! de promoción de la salud cardiovascular en adolescentes, llevado a cabo por la Fundación SHE, la Fundación Centro Nacional de Investigaciones Cardiovasculares Carlos III y la Universidad de Barcelona.

Este programa se va a realizar en 24 centros de educación secundaria de la Comunidad de Madrid y en Cataluña. Como tu instituto ha sido elegido, tienes la oportunidad de participar como voluntario en este programa.

¿Qué vamos a hacer?

Tu estilo de vida (alimentación, actividad física, sedentarismo, etc) durante la adolescencia influirá en tu salud cardiovascular cuando seas adulto. Por ello, queremos **conocer la salud cardiovascular y los estilos de vida** de los adolescentes al inicio de la educación secundaria (inicio del primer curso), a la mitad (fin del segundo curso) y al final de la misma (fin del cuarto curso) mediante una serie de pruebas:

- Cuestionarios: cuestionario de alimentación, cuestionario de conocimientos, actitudes y hábitos acerca de los estilos de vida, y un cuestionario para tus padres.

- Medición de: peso, altura, circunferencia de cintura, porcentaje de grasa, presión arterial, actividad física mediante acelerómetros (pequeño dispositivo que se lleva en la cintura), parámetros bioquímicos (colesterol, glucosa, triglicéridos) mediante una gota de sangre obtenida de un dedo y medición de compuestos de la dieta a través de una muestra de orina y saliva.

Además, a lo largo de la educación secundaria, recibiréis dentro del horario escolar una serie de contenidos dirigidos a mejorar vuestros conocimientos y actitudes hacia los estilos de vida saludables.

Una vez acabadas las pruebas, recibirás un informe con los resultados de los análisis de sangre y de las mediciones corporales, para conocer tu estado general de salud. Las muestras que obtengamos no se usarán para ningún otro fin, y serán destruidas una vez realizados los análisis. Tampoco usaremos tus muestras para realizar análisis genéticos de ningún tipo.

Tu participación en el estudio es voluntaria y gratuita. Si deseas participar, necesitamos tu firma y la de tus padres. Si en algún momento deseas retirarte del estudio, puedes hacerlo en cualquier momento y sin dar explicaciones de ningún tipo.

Toda la información recogida en este estudio será estrictamente confidencial. Eso significa que nadie podrá relacionar tu nombre con los resultados obtenidos. En todo el proceso se seguirá la Ley de Protección de Datos (Ley orgánica 15/1999 de 13 de diciembre) y otras leyes vigentes aplicables.

Con tu ayuda podremos mejorar la salud cardiovascular de la población española.

¡MUCHAS GRACIAS POR TU COLABORACIÓN!

Este proyecto ha sido aprobado por el Comité de Ética del Instituto de Salud Carlos III, la Fundació Unió Catalana d'Hospitals y la Comissió de Bioètica de la Universitat de Barcelona

Madrid, 10 de enero del 2017

Estimados padres y madres,

El instituto de su hijo/a ha sido seleccionado para participar en el Programa SI! de promoción de la salud cardiovascular en adolescentes, llevado a cabo por la Fundación SHE, la Fundación Centro Nacional de Investigaciones Cardiovasculares Carlos III y la Universidad de Barcelona.

Este programa se llevará a cabo **desde septiembre de 2017 hasta junio 2021** en 24 centros de educación secundaria situados en la Comunidad de Madrid y en Cataluña. Ustedes tienen por tanto la oportunidad de participar como voluntarios en este programa.

Objetivos del programa

La adolescencia es un período crítico durante el cual se establecen y refuerzan estilos de vida (alimentación, actividad física, sedentarismo, etc) que condicionarán la salud cardiovascular en la etapa adulta. Por esta razón el objetivo de esta iniciativa es **valorar y mejorar la salud cardiovascular y los estilos de vida** de los adolescentes al inicio de la educación secundaria (inicio del primer curso), a la mitad (fin del segundo curso) y al final de la misma (fin del cuarto curso). Las pruebas a realizar incluyen:

- Cuestionario de alimentación (cuestionario de frecuencia de consumo de alimentos).
- Cuestionario general acerca de conocimientos, actitudes y hábitos acerca de los estilos de vida.
- Mediciones antropométricas a los adolescentes: peso, altura, circunferencia de cintura y porcentaje de grasa.
- Uso de acelerómetros para el registro objetivo de la actividad física (pequeño dispositivo que se lleva en la cintura).
- Medición de la presión arterial.
- Medición de parámetros bioquímicos (colesterol total, HDL, LDL, triglicéridos y glucosa) mediante la extracción de una gota de sangre capilar.
- Determinación de compuestos dietéticos (polifenoles y metabolitos) en una muestra de orina y saliva.
- Cuestionario general dirigido a padres y madres acerca de aspectos sociodemográficos y estilos de vida (tabaquismo, dieta, actividad física, etc).

Además, los alumnos se beneficiarán de una intervención educativa llevada a cabo dentro del programa curricular del instituto, con la ayuda de los profesores del centro, y que ha sido diseñada por el equipo pedagógico de la Fundación SHE. Dicha intervención educativa beneficiará a su hijo/a ayudando a aumentar su conocimiento acerca de la salud cardiovascular, los estilos de vida saludables y la prevención de hábitos nocivos, con un impacto positivo esperado en su perfil de salud cardiovascular.

Adicionalmente, nos gustaría realizar una última valoración de seguimiento cuando los participantes alcancen los veinte años de edad. Para ello, le pedimos que nos autorice a ser contactados en el futuro.

Las familias recibirán un correo electrónico con un enlace para cumplimentar el cuestionario dirigido a padres y madres. Una vez finalizada la recogida de datos, se hará llegar a las familias un informe con los resultados principales derivados de los análisis bioquímicos, antropométricos y de presión arterial relativos al estado de salud de sus hijos. Todas las muestras serán analizadas bajo los controles de calidad pertinentes. Las muestras servirán para valorar el estado de salud general de los participantes. Las muestras sobrantes no se usarán en ninguna otra investigación y serán destruidas. No se realizarán análisis genéticos a partir de las muestras obtenidas.

La participación en el estudio es voluntaria y gratuita:

Nos gustaría aclarar que tanto la participación de su hijo/a como la suya son voluntarias y gratuitas. **Usted, su hijo/a y/o su familia es libre de retirarse del estudio en cualquier momento y sin dar explicaciones**.

Riesgos de participar en el proyecto:

La máxima molestia que puede percibir es un ligero pinchazo en el pulpejo del dedo al realizar una punción para extracción de una gota de sangre capilar.

Confidencialidad

Este proyecto ha sido aprobado por el Comité de Ética del Instituto de Salud Carlos III, la Fundació Unió Catalana d'Hospitals y la Comissió de Bioètica de la Universitat de Barcelona y cuenta con la colaboración de la Fundación SHE, la Fundación Centro Nacional de Investigaciones Cardiovasculares Carlos III y la Universidad de Barcelona.

Toda la información recogida en este estudio será estrictamente confidencial. Toda la información será tratada de forma anónima mediante su codificación. Los nombres y direcciones o vías de contacto estarán completamente separados de los datos obtenidos en el estudio, bajo llave. En todo el proceso se seguirá la Ley de Protección de Datos (Ley orgánica 15/1999 de 13 de diciembre) y otras leyes vigentes aplicables.

Los resultados anónimos derivados del estudio serán de gran ayuda para los responsables de elaborar las políticas de salud pública en el futuro, con el objetivo de mejorar la salud cardiovascular de la población española.

MUCHAS GRACIAS POR SU ATENCION Y COLABORACIÓN

Detalles de contacto

Para cualquier consulta puede dirigirse a nuestro equipo a través del **correo electrónico** estudio@fundacionshe.org o en el **teléfono** 93 218 54 44.

HOJA DE CONSENTIMIENTO INFORMADO-PROGRAMA SI! SECUNDARIA

TÍTULO DEL PROYECTO: Intervención educativa en escolares para hacer frente a la obesidad y promover la salud cardiovascular en adolescentes españoles: un ensayo controlado y aleatorizado por grupos.

Centros participantes: Campus de la Alimentación de Torribera. Departamento de Nutrición, Bromatología y Gastronomía de la Facultad de Farmacia (Universidad de Barcelona); Centro Nacional de Investigaciones Cardiovasculares Carlos III (CNIC); Foundation for Science Health and Education (Fundación SHE)

Investigadora principal: Dra. Rosa Mª Lamuela

Yo (nombre y apellidos) ……

autorizo a mi hija/o (nombre y apellidos) ……………………………………………….…………..........................del

instituto……….a participar en el estudio.

- He leído la hoja de información que acompaña a este consentimiento.
- He podido hacer preguntas sobre el estudio.
- He recibido suficiente información sobre el estudio.
- Se me ha informado de que los datos personales recogidos en el presente estudio se tratarán confidencialmente de acuerdo con la Ley orgánica 15/1999 de Protección de Datos Personales, y su normativa de desarrollo.
- Se me ha informado de que los datos obtenidos sólo se utilizarán para los fines específicos del estudio.

Comprendo que la participación de mi hijo/a y la de su familia es voluntaria. Comprendo que tanto mi hijo/a como su familia pueden retirarse del estudio cuando quieran y sin tener que dar explicaciones. Además, se me ha facilitado un contacto con los investigadores del estudio para solucionar cualquier duda que pudiera surgir al respecto.

Damos libremente nuestra conformidad para participar en el estudio:

Correo electrónico: ………………………………………………………. Teléfono: ………………………../……………………………

___________________ ________________________ _______________________
(Fecha) (Firma del adolescente) (Firma de la madre, padre o tutor legal)*

_____________________ _____________________________
(Fecha) (Firma del investigador)

☐ Autorizo a ser contactado/a cuando mi hijo/a alcance los 20 años a través del correo electrónico y teléfono anteriormente facilitado.

** Si la tutela es compartida, deberán firmar ambos progenitores, (en el caso de que solo firme uno, debe haber una delegación por escrito del que no está presente al que sí lo está).*

Copia para el padre y/o madre/ tutor.

Este proyecto ha sido aprobado por el Comité de Ética del Instituto de Salud Carlos III, la Fundació Unió Catalana d'Hospitals y la Comissió de Bioètica de la Universitat de Barcelona

HOJA DE CONSENTIMIENTO INFORMADO-PROGRAMA SI! SECUNDARIA

TÍTULO DEL PROYECTO: Intervención educativa en escolares para hacer frente a la obesidad y promover la salud cardiovascular en adolescentes españoles: un ensayo controlado y aleatorizado por grupos.

Centros participantes: Campus de la Alimentación de Torribera. Departamento de Nutrición, Bromatología y Gastronomía de la Facultad de Farmacia (Universidad de Barcelona); Centro Nacional de Investigaciones Cardiovasculares Carlos III (CNIC); Foundation for Science Health and Education (Fundación SHE)

Investigadora principal: Dra. Rosa Mª Lamuela

Yo (nombre y apellidos) ……

autorizo a mi hija/o (nombre y apellidos) …………………………………………………..………..........................del

instituto…….a participar en el estudio.

- He leído la hoja de información que acompaña a este consentimiento.
- He podido hacer preguntas sobre el estudio.
- He recibido suficiente información sobre el estudio.
- Se me ha informado de que los datos personales recogidos en el presente estudio se tratarán confidencialmente de acuerdo con la Ley orgánica 15/1999 de Protección de Datos Personales, y su normativa de desarrollo.
- Se me ha informado de que los datos obtenidos sólo se utilizarán para los fines específicos del estudio.

Comprendo que la participación de mi hijo/a y la de su familia es voluntaria. Comprendo que tanto mi hijo/a como su familia pueden retirarse del estudio cuando quieran y sin tener que dar explicaciones. Además, se me ha facilitado un contacto con los investigadores del estudio para solucionar cualquier duda que pudiera surgir al respecto.

Damos libremente nuestra conformidad para participar en el estudio:

Correo electrónico: ……………………………………………………….. Teléfono: ………………………../……………………………

___________________	________________________	_______________________
(Fecha)	(Firma del adolescente)	(Firma de la madre, padre o tutor legal)*

_____________________	_____________________________
(Fecha)	(Firma del investigador)

☐ Autorizo a ser contactado/a cuando mi hijo/a alcance los 20 años a través del correo electrónico y teléfono anteriormente facilitado.

** Si la tutela es compartida, deberán firmar ambos progenitores, (en el caso de que solo firme uno, debe haber una delegación por escrito del que no está presente al que sí lo está).*

Copia para el investigador principal

Este proyecto ha sido aprobado por el Comité de Ética del Instituto de Salud Carlos III, la Fundació Unió Catalana d'Hospitals y la Comissió de Bioètica de la Universitat de Barcelona

Barcelona, 10 de gener de 2017

Benvolgut/uda alumne/a,

El teu institut ha estat seleccionat per participar en el Programa SI! de promoció de la salut cardiovascular en adolescents, que duen a terme la Fundació SHE, la Fundació Centre Nacional d'Investigacions Cardiovasculars Carlos III i la Universitat de Barcelona.

Aquest programa s'implementarà a 24 centres d'educació secundària de la Comunitat de Madrid i a Catalunya. Com que el teu institut ha estat seleccionat, tens l'oportunitat de participar com a voluntari d'aquest programa.

Què farem?

El teu estil de vida (alimentació, activitat física, sedentarisme...) durant l'adolescència influirà en la teva salut cardiovascular quan siguis adult. Per això, volem **conèixer la salut cardiovascular i els estils de vida** dels adolescents en començar l'educació secundària (inici del primer curs), a la meitat d'aquesta (fi del segon curs) i al final (fi del quart curs), mitjançant una sèrie de proves, com ara:

- Qüestionaris: qüestionari d'alimentació, de coneixements, d'actituds i d'hàbits sobre els estils de vida, i un darrer qüestionari per als teus pares.

- Mesurament de: pes, estatura, circumferència de cintura, percentatge de greix, tensió arterial, activitat física mitjançant acceleròmetres (petit dispositiu que es duu a la cintura), paràmetres bioquímics (colesterol, glucosa, triglicèrids) mitjançant una gota de sang extreta d'un dit i mesurament de components de la dieta mitjançant una mostra d'orina i saliva.

- Anàlisi de la composició corporal a partir d'una densitometria òssia (DEXA). L'acceptació de participar en aquest estudi no obliga a realitzar aquesta prova, fer-la és opcional.

A més, al llarg de l'educació secundària, rebreu dins de l'horari escolar una sèrie de continguts dirigits a millorar els vostres coneixements i actituds envers els estils de vida saludables.

Un cop enllestides les proves, rebràs un informe amb els resultats de les anàlisis de sang, dels mesuraments corporals i de l'anàlisi de la composició corporal a partir del DEXA (si s'ha fet), per tal de conèixer el teu estat general de salut. Les mostres que obtinguem no es faran servir amb cap altra finalitat i seran destruïdes un cop realitzades les anàlisis. Tampoc no farem servir les teves mostres per dur a terme anàlisis genètiques de cap mena.

La teva participació en l'estudi és voluntària i gratuïta. Si desitges participar-hi, necessitarem la teva signatura i la dels teus pares. Si en algun moment desitges abandonar l'estudi, pots fer-ho quan vulguis i sense donar cap tipus d'explicacions.

Tota la informació recollida en aquest estudi serà tractada de forma estrictament confidencial. Això vol dir que ningú no podrà relacionar el teu nom amb els resultats obtinguts. Durant tot el procés, es respectarà la Llei de protecció de dades (Llei orgànica 15/1999 de 13 de desembre) i altres lleis vigents aplicables.

Amb la teva ajuda podrem millorar la salut cardiovascular de la població espanyola.

MOLTES MERCÈS PER LA TEVA COL.LABORACIÓ!

Programa de Salut Integral

Barcelona, 10 de gener de 2017

Benvolguts pares i mares,

L'institut del seu fill/a ha estat seleccionat per participar en el Programa SI! de promoció de la salut cardiovascular en adolescents, que duen a terme la Fundació SHE, la Fundació Centre Nacional d'Investigacions Cardiovasculars Carlos III i la Universitat de Barcelona.

Aquest programa s'implantarà **des del setembre de 2017 fins al juny de 2021** a 24 centres d'educació secundària situats a la Comunitat de Madrid i a Catalunya. Vostès tenen l'oportunitat de participar com a voluntaris d'aquest programa.

Objectius del programa

L'adolescència és un període crític durant el qual s'estableixen i reforcen estils de vida (alimentació, activitat física, sedentarisme, etc.) que condicionaran la salut cardiovascular durant l'etapa adulta. Per aquest motiu, l'objectiu d'aquesta iniciativa és **valorar i millorar la salut cardiovascular i els estils de vida** dels adolescents a l'inici de l'educació secundària (inici del primer curs), a la meitat d'aquesta (fi del segons curs) i al final (fi del quart curs). Les proves que farem inclouen:

- Qüestionari d'alimentació (qüestionari de freqüència de consum d'aliments).
- Qüestionari general sobre coneixements, actituds i hàbits sobre els estils de vida.
- Mesuraments antropomètrics als adolescents: pes, estatura, circumferència de cintura i percentatge de greix.
- Ús d'acceleròmetres pel registre objectiu de l'activitat física (petit dispositiu que es porta a la cintura).
- Mesurament de la pressió arterial.
- Mesurament de paràmetres bioquímics (colesterol total, HDL, LDL, triglicèrids i glucosa) mitjançant l'extracció d'una gota de sang capil·lar.
- Determinació de compostos dietètics (polifenols i metabòlits) en una mostra d'orina i saliva.
- Anàlisi de la composició corporal mitjançant una densitometria òssia (DEXA). Aquesta prova serà de caràcter opcional.
- Qüestionari general dirigit a pares i mares sobre aspectes sociodemogràfics i estils de vida (tabaquisme, dieta, activitat física, etc.).

A més a més, els alumnes es beneficiaran d'una intervenció educativa duta a terme dins del programa curricular de l'institut, amb l'ajut dels professors del centre, i que ha estat dissenyada per l'equip pedagògic de la Fundació SHE. Aquesta intervenció educativa beneficiarà el/la seu/va fill/a, gràcies a l'aprofundiment del seu coneixement sobre la salut cardiovascular, els estils de vida saludables i la prevenció d'hàbits nocius, amb un impacte positiu esperat en el seu perfil de salut cardiovascular.

Addicionalment, ens agradaria fer una darrera valoració de seguiment quan els participants compleixin vint anys. Per aquest motiu, li demanem que ens autoritzi a contactar-los de nou en el futur.

Les famílies rebran un correu electrònic amb un enllaç per poder emplenar el qüestionari dirigit a pares i mares. Un cop finalitzada la recollida de dades, es farà arribar a les famílies un informe amb els principals resultats derivats de les anàlisis bioquímiques, antropomètriques, de pressió arterial i de composició corporal obtinguts del DEXA (si s'ha fet) relatius a l'estat de salut del seus fills. Totes les mostres seran analitzades sota els controls de qualitat pertinents. Les mostres serviran per valorar l'estat de salut general dels participants. Les mostres sobrants no es faran servir a cap altre treball de recerca i seran destruïdes. No es farà cap anàlisi genètica a partir de les mostres obtingudes.

La participació en l'estudi és voluntària i gratuïta:

Ens agradaria deixar clar que tant la participació del/de la seu/va fill/a com la seva són voluntàries i gratuïtes. **Vostè, el/la seu/va fill/a i/o la seva família són lliures d'abandonar l'estudi en qualsevol moment i sense donar explicacions**.

Riscos de participar en el projecte:

La realització de la prova de DEXA suposa una dosi de radiació mínima (gairebé negligible), similar a la rebuda en un viatge d'avió d'una hora de durada o un dia de radiació ambiental natural. La màxima molèstia que pot percebre és una petita punxada al palpís del dit en punxar-li per extraure'n una gota de sang capil·lar.

Confidencialitat

Aquest projecte ha estat aprovat pel Comitè d'Ètica de l'Institut de Salut Carlos III, la Fundació Unió Catalana d'Hospitals i la Comissió de Bioètica de la Universitat de Barcelona i compta amb la col·laboració de la Fundació SHE, la Fundació Centre Nacional d'Investigacions Cardiovasculars Carlos III i la Universitat de Barcelona.

Tota la informació recollida en aquest estudi serà estrictament confidencial. Tota la informació serà tractada de forma anònima mitjançant la seva codificació. Els noms i adreces o altres dades de contacte estaran completament separades de les dades obtingudes a l'estudi, sota clau. Durant tot el procés se seguirà la Llei de protecció de dades (Llei orgànica 15/1999 de 13 de desembre) i altres lleis vigents aplicables.

Els resultats anònims derivats de l'estudi seran de gran ajuda per als responsables d'elaborar les polítiques de salut pública en el futur, amb l'objectiu de millorar la salut cardiovascular de la població espanyola.

MOLTES MERCÈS PER LA SEVA ATENCIÓ I COL.LABORACIÓ.

Detalls de contacte

Per a qualsevol consulta pot comunicar-se amb el nostre equip mitjançant el **correu electrònic** estudio@fundacionshe.org o bé trucant al **telèfon** 93 218 54 44.

FULL DE CONSENTIMENT INFORMAT-PROGRAMA SI! SECUNDÀRIA

TÍTOL DEL PROJECTE: Intervenció educativa en escolars per fer front a l'obesitat i promoure la salut cardiovascular en adolescents espanyols: un assaig controlat i aleatoritzat per grups.

Centres participants: Campus de l'Alimentació de Torribera. Departament de Nutrició, Bromatologia i Gastronomia de la Facultat de Farmàcia (Universitat de Barcelona); Centre Nacional d'Investigacions Cardiovasculars Carlos III (CNIC); Fundació SHE (Foundation for Science Health and Education).

Investigadora principal: Dra. Rosa M. Lamuela

Jo (nom i cognoms) ……

autoritzo el/la meu/va fill/a (nom i cognoms)…………………………………………………..........................de

l'institut………………………………………………………………………………………………………........…a participar en l'estudi.

- He llegit el full informatiu que acompanya aquest consentiment.
- He pogut fer preguntes sobre l'estudi.
- He rebut suficient informació sobre l'estudi.
- He estat informat/ada que les dades personals recollides en aquest estudi es tractaran de forma estrictament confidencial, segons la Llei orgànica 15/1999 de Protecció de dades personals i la seva normativa de desenvolupament.
- He estat informat/ada que les dades obtingudes només es faran servir per a la finalitat específica d'aquest estudi.

Comprenc que la participació del/de la meu/va fill/a i de la seva família és voluntària. Comprenc que tant el/la meu/va fill/a com la seva família poden abandonar l'estudi quan vulguin sense haver de donar explicacions. A més a més, m'han facilitat les dades de contacte dels investigadors de l'estudi per tal de poder solucionar qualsevol dubte que pogués sorgir referent a aquest estudi.

Donem lliurement la nostra conformitat per participar en l'estudi:

☐ En la seva totalitat ☐ Sense densitometria òssia (DEXA)

Correu electrònic: ………………………………………………………. Telèfon: ………………………./……………………………

____________________ _________________________ ________________________

(Data) (Signatura de l'adolescent) (Signatura de la mare, pare o tutor legal)*

______________________ ______________________________

(Data) (Signatura de l'investigador)

☐ Autoritzo a ser contactat/ada quan el/la meu/va fill/a tingui 20 anys mitjançant el correu electrònic i el telèfon anteriorment indicats.

** Si la tutela és compartida, hauran de signar ambdós progenitors (si només signés un, cal adjuntar una delegació escrita del progenitor absent al present).*

Còpia per al pare, mare o tutor.

Aquest projecte ha estat aprovat pel Comitè d'ètica del Instituto de Salud Carlos III, la Fundació Unió Catalana d'Hospitals i la Comissió de Bioètica de la Universitat de Barcelona.

Sí! Programa de Salut Integral

FULL DE CONSENTIMENT INFORMAT-PROGRAMA SI! SECUNDÀRIA

TÍTOL DEL PROJECTE: Intervenció educativa en escolars per fer front a l'obesitat i promoure la salut cardiovascular en adolescents espanyols: un assaig controlat i aleatoritzat per grups.

Centres participants: Campus de l'Alimentació de Torribera. Departament de Nutrició, Bromatologia i Gastronomia de la Facultat de Farmàcia (Universitat de Barcelona); Centre Nacional d'Investigacions Cardiovasculars Carlos III (CNIC); Fundació SHE (Foundation for Science Health and Education).

Investigadora principal: Dra. Rosa M. Lamuela

Jo (nom i cognoms) ……

autoritzo el/la meu/va fill/a (nom i cognoms)…………………………………………………...……………......................de

l'institut……...……a participar en l'estudi.

- He llegit el full informatiu que acompanya aquest consentiment.
- He pogut fer preguntes sobre l'estudi.
- He rebut suficient informació sobre l'estudi.
- He estat informat/ada que les dades personals recollides en aquest estudi es tractaran de forma estrictament confidencial, segons la Llei orgànica 15/1999 de Protecció de dades personals i la seva normativa de desenvolupament.
- He estat informat/ada que les dades obtingudes només es faran servir per a la finalitat específica d'aquest estudi.

Comprenc que la participació del/de la meu/va fill/a i de la seva família és voluntària. Comprenc que tant el/la meu/va fill/a com la seva família poden abandonar l'estudi quan vulguin sense haver de donar explicacions. A més a més, m'han facilitat les dades de contacte dels investigadors de l'estudi per tal de poder solucionar qualsevol dubte que pogués sorgir referent a aquest estudi.

Donem lliurement la nostra conformitat per participar en l'estudi:

☐ En la seva totalitat ☐ Sense densitometria òssia (DEXA)

Correu electrònic: ………………………………………………………… Telèfon: ………………………./……………………………

____________________	_________________________	________________________
(Data)	(Signatura de l'adolescent)	(Signatura de la mare, pare o tutor legal)*

______________________	______________________________
(Data)	(Signatura de l'investigador)

☐ Autoritzo a ser contactat/ada quan el/la meu/va fill/a tingui 20 anys mitjançant el correu electrònic i el telèfon anteriorment indicats.

** Si la tutela és compartida, hauran de signar ambdós progenitors (si només signés un, cal adjuntar una delegació escrita del progenitor absent al present).*

Còpia per a l'investigador principal

Aquest projecte ha estat aprovat pel Comitè d'ètica del Instituto de Salud Carlos III, la Fundació Unió Catalana d'Hospitals i la Comissió de Bioètica de la Universitat de Barcelona.

SHE Foundation for Science, Health and Education — cnic Fundación Centro Nacional de Investigaciones Cardiovasculares Carlos III — UNIVERSITAT DE BARCELONA

Hoja informativa para Adolescentes
Versión 10-01-2017

Madrid, 10 de enero del 2017

Estimado/a alumno/a,

Tu instituto ha sido seleccionado para participar en el Programa SI! de promoción de la salud cardiovascular en adolescentes, llevado a cabo por la Fundación SHE, la Fundación Centro Nacional de Investigaciones Cardiovasculares Carlos III y la Universidad de Barcelona.

Este programa se va a realizar en 24 centros de educación secundaria de la Comunidad de Madrid y en Cataluña. Como tu instituto ha sido elegido, tienes la oportunidad de participar como voluntario en este programa.

¿Qué vamos a hacer?

Tu estilo de vida (alimentación, actividad física, sedentarismo, etc) durante la adolescencia influirá en tu salud cardiovascular cuando seas adulto. Por ello, queremos **conocer la salud cardiovascular y los estilos de vida** de los adolescentes al inicio de la educación secundaria (inicio del primer curso), a la mitad (fin del segundo curso) y al final de la misma (fin del cuarto curso) mediante una serie de pruebas:

- Cuestionarios: cuestionario de alimentación, cuestionario de conocimientos, actitudes y hábitos acerca de los estilos de vida, y un cuestionario para tus padres.

- Medición de: peso, altura, circunferencia de cintura, porcentaje de grasa, presión arterial, actividad física mediante acelerómetros (pequeño dispositivo que se lleva en la cintura), parámetros bioquímicos (colesterol, glucosa, triglicéridos) mediante una gota de sangre obtenida de un dedo y medición de compuestos de la dieta a través de una muestra de orina y saliva.

Una vez acabadas las pruebas, recibirás un informe con los resultados de los análisis de sangre y de las mediciones corporales, para conocer tu estado general de salud. Las muestras que obtengamos no se usarán para ningún otro fin, y serán destruidas una vez realizados los análisis. Tampoco usaremos tus muestras para realizar análisis genéticos de ningún tipo.

Tu participación en el estudio es voluntaria y gratuita. Si deseas participar, necesitamos tu firma y la de tus padres. Si en algún momento deseas retirarte del estudio, puedes hacerlo en cualquier momento y sin dar explicaciones de ningún tipo.

Toda la información recogida en este estudio será estrictamente confidencial. Eso significa que nadie podrá relacionar tu nombre con los resultados obtenidos. En todo el proceso se seguirá la Ley de Protección de Datos (Ley orgánica 15/1999 de 13 de diciembre) y otras leyes vigentes aplicables.

Con tu ayuda podremos mejorar la salud cardiovascular de la población española.

¡MUCHAS GRACIAS POR TU COLABORACIÓN!

Madrid, 10 de enero del 2017

Estimados padres y madres,

El instituto de su hijo/a ha sido seleccionado para participar en el Programa SI! de promoción de la salud cardiovascular en adolescentes, llevado a cabo por la Fundación SHE, la Fundación Centro Nacional de Investigaciones Cardiovasculares Carlos III y la Universidad de Barcelona.

Este programa se llevará a cabo **desde septiembre de 2017 hasta junio 2021** en 24 centros de educación secundaria situados en la Comunidad de Madrid y en Cataluña. Ustedes tienen por tanto la oportunidad de participar como voluntarios en este programa.

Objetivos del programa

La adolescencia es un período crítico durante el cual se establecen y refuerzan estilos de vida (alimentación, actividad física, sedentarismo, etc) que condicionarán la salud cardiovascular en la etapa adulta. Por esta razón el objetivo de esta iniciativa es **valorar y mejorar la salud cardiovascular y los estilos de vida** de los adolescentes al inicio de la educación secundaria (inicio del primer curso), a la mitad (fin del segundo curso) y al final de la misma (fin del cuarto curso). Las pruebas a realizar incluyen:

- Cuestionario de alimentación (cuestionario de frecuencia de consumo de alimentos).
- Cuestionario general acerca de conocimientos, actitudes y hábitos acerca de los estilos de vida.
- Mediciones antropométricas a los adolescentes: peso, altura, circunferencia de cintura y porcentaje de grasa.
- Uso de acelerómetros para el registro objetivo de la actividad física (pequeño dispositivo que se lleva en la cintura).
- Medición de la presión arterial.
- Medición de parámetros bioquímicos (colesterol total, HDL, LDL, triglicéridos y glucosa) mediante la extracción de una gota de sangre capilar.
- Determinación de compuestos dietéticos (polifenoles y metabolitos) en una muestra de orina y saliva.
- Cuestionario general dirigido a padres y madres acerca de aspectos sociodemográficos y estilos de vida (tabaquismo, dieta, actividad física, etc).

Adicionalmente, nos gustaría realizar una última valoración de seguimiento cuando los participantes alcancen los veinte años de edad. Para ello, le pedimos que nos autorice a ser contactados en el futuro.

Las familias recibirán un correo electrónico con un enlace para cumplimentar el cuestionario dirigido a padres y madres. Una vez finalizada la recogida de datos, se hará llegar a las familias un informe con los resultados principales derivados de los análisis bioquímicos, antropométricos y de presión arterial relativos al estado de salud de sus hijos. Todas las muestras serán analizadas bajo los controles de calidad pertinentes. Las muestras servirán para valorar el estado de salud general

de los participantes. Las muestras sobrantes no se usarán en ninguna otra investigación y serán destruidas. No se realizarán análisis genéticos a partir de las muestras obtenidas.

La participación en el estudio es voluntaria y gratuita:

Nos gustaría aclarar que tanto la participación de su hijo/a como la suya son voluntarias y gratuitas. **Usted, su hijo/a y/o su familia es libre de retirarse del estudio en cualquier momento y sin dar explicaciones**.

Riesgos de participar en el proyecto:

La máxima molestia que puede percibir es un ligero pinchazo en el pulpejo del dedo al realizar una punción para extracción de una gota de sangre capilar.

Confidencialidad

Este proyecto ha sido aprobado por el Comité de Ética del Instituto de Salud Carlos III, la Fundació Unió Catalana d'Hospitals y la Comissió de Bioètica de la Universitat de Barcelona y cuenta con la colaboración de la Fundación SHE, la Fundación Centro Nacional de Investigaciones Cardiovasculares Carlos III y la Universidad de Barcelona.

Toda la información recogida en este estudio será estrictamente confidencial. Toda la información será tratada de forma anónima mediante su codificación. Los nombres y direcciones o vías de contacto estarán completamente separados de los datos obtenidos en el estudio, bajo llave. En todo el proceso se seguirá la Ley de Protección de Datos (Ley orgánica 15/1999 de 13 de diciembre) y otras leyes vigentes aplicables.

Los resultados anónimos derivados del estudio serán de gran ayuda para los responsables de elaborar las políticas de salud pública en el futuro, con el objetivo de mejorar la salud cardiovascular de la población española.

MUCHAS GRACIAS POR SU ATENCION Y COLABORACIÓN

Detalles de contacto

Para cualquier consulta puede dirigirse a nuestro equipo a través del **correo electrónico** estudio@fundacionshe.org o en el **teléfono** 93 218 54 44.

HOJA DE CONSENTIMIENTO INFORMADO-PROGRAMA SI! SECUNDARIA

TÍTULO DEL PROYECTO: Intervención educativa en escolares para hacer frente a la obesidad y promover la salud cardiovascular en adolescentes españoles: un ensayo controlado y aleatorizado por grupos.

Centros participantes: Campus de la Alimentación de Torribera. Departamento de Nutrición, Bromatología y Gastronomía de la Facultad de Farmacia (Universidad de Barcelona); Centro Nacional de Investigaciones Cardiovasculares Carlos III (CNIC); Foundation for Science Health and Education (Fundación SHE)

Investigadora principal: Dra. Rosa Mª Lamuela

Yo (nombre y apellidos) ………

autorizo a mi hija/o (nombre y apellidos) ……………………………………………….……………...........................del

instituto……….a participar en el estudio.

- He leído la hoja de información que acompaña a este consentimiento.
- He podido hacer preguntas sobre el estudio.
- He recibido suficiente información sobre el estudio.
- Se me ha informado de que los datos personales recogidos en el presente estudio se tratarán confidencialmente de acuerdo con la Ley orgánica 15/1999 de Protección de Datos Personales, y su normativa de desarrollo.
- Se me ha informado de que los datos obtenidos sólo se utilizarán para los fines específicos del estudio.

Comprendo que la participación de mi hijo/a y la de su familia es voluntaria. Comprendo que tanto mi hijo/a como su familia pueden retirarse del estudio cuando quieran y sin tener que dar explicaciones. Además, se me ha facilitado un contacto con los investigadores del estudio para solucionar cualquier duda que pudiera surgir al respecto.

Damos libremente nuestra conformidad para participar en el estudio:

Correo electrónico: ………………………………………………………. Teléfono: ………………………../……………………………

____________________	_________________________	________________________
(Fecha)	(Firma del adolescente)	(Firma de la madre, padre o tutor legal)*

_____________________	______________________________
(Fecha)	(Firma del investigador)

☐ Autorizo a ser contactado/a cuando mi hijo/a alcance los 20 años a través del correo electrónico y teléfono anteriormente facilitado.

* *Si la tutela es compartida, deberán firmar ambos progenitores, (en el caso de que solo firme uno, debe haber una delegación por escrito del que no está presente al que sí lo está).*

Copia para el padre y/o madre/ tutor.

Este proyecto ha sido aprobado por el Comité de Ética del Instituto de Salud Carlos III, la Fundació Unió Catalana d'Hospitals y la Comissió de Bioètica de la Universitat de Barcelona

HOJA DE CONSENTIMIENTO INFORMADO-PROGRAMA SI! SECUNDARIA

TÍTULO DEL PROYECTO: Intervención educativa en escolares para hacer frente a la obesidad y promover la salud cardiovascular en adolescentes españoles: un ensayo controlado y aleatorizado por grupos.

Centros participantes: Campus de la Alimentación de Torribera. Departamento de Nutrición, Bromatología y Gastronomía de la Facultad de Farmacia (Universidad de Barcelona); Centro Nacional de Investigaciones Cardiovasculares Carlos III (CNIC); Foundation for Science Health and Education (Fundación SHE)

Investigadora principal: Dra. Rosa Mª Lamuela

Yo (nombre y apellidos) ………

autorizo a mi hija/o (nombre y apellidos) ……………………………………………………..…………..........................del

instituto……….a participar en el estudio.

- He leído la hoja de información que acompaña a este consentimiento.
- He podido hacer preguntas sobre el estudio.
- He recibido suficiente información sobre el estudio.
- Se me ha informado de que los datos personales recogidos en el presente estudio se tratarán confidencialmente de acuerdo con la Ley orgánica 15/1999 de Protección de Datos Personales, y su normativa de desarrollo.
- Se me ha informado de que los datos obtenidos sólo se utilizarán para los fines específicos del estudio.

Comprendo que la participación de mi hijo/a y la de su familia es voluntaria. Comprendo que tanto mi hijo/a como su familia pueden retirarse del estudio cuando quieran y sin tener que dar explicaciones. Además, se me ha facilitado un contacto con los investigadores del estudio para solucionar cualquier duda que pudiera surgir al respecto.

Damos libremente nuestra conformidad para participar en el estudio:

Correo electrónico: ………………………………………………………… Teléfono: ………………………../……………………………

___________________	________________________	_______________________
(Fecha)	(Firma del adolescente)	(Firma de la madre, padre o tutor legal)*

_____________________	_____________________________
(Fecha)	(Firma del investigador)

☐ Autorizo a ser contactado/a cuando mi hijo/a alcance los 20 años a través del correo electrónico y teléfono anteriormente facilitado.

** Si la tutela es compartida, deberán firmar ambos progenitores, (en el caso de que solo firme uno, debe haber una delegación por escrito del que no está presente al que sí lo está).*

Copia para el investigador principal.

Este proyecto ha sido aprobado por el Comité de Ética del Instituto de Salud Carlos III, la Fundació Unió Catalana d'Hospitals y la Comissió de Bioètica de la Universitat de Barcelona

Programa de Salut Integral

Barcelona, 10 de gener de 2017

Benvolgut/uda alumne/a,

El teu institut ha estat seleccionat per participar en el Programa SI! de promoció de la salut cardiovascular en adolescents, que duen a terme la Fundació SHE, la Fundació Centre Nacional d'Investigacions Cardiovasculars Carlos III i la Universitat de Barcelona.

Aquest programa s'implementarà a 24 centres d'educació secundària de la Comunitat de Madrid i a Catalunya. Com que el teu institut ha estat seleccionat, tens l'oportunitat de participar com a voluntari d'aquest programa.

Què farem?

El teu estil de vida (alimentació, activitat física, sedentarisme...) durant l'adolescència influirà en la teva salut cardiovascular quan siguis adult. Per això, volem **conèixer la salut cardiovascular i els estils de vida** dels adolescents en començar l'educació secundària (inici del primer curs), a la meitat d'aquesta (fi del segon curs) i al final (fi del quart curs), mitjançant una sèrie de proves, com ara:

- Qüestionaris: qüestionari d'alimentació, de coneixements, d'actituds i d'hàbits sobre els estils de vida, i un darrer qüestionari per als teus pares.

- Mesurament de: pes, estatura, circumferència de cintura, percentatge de greix, tensió arterial, activitat física mitjançant acceleròmetres (petit dispositiu que es duu a la cintura), paràmetres bioquímics (colesterol, glucosa, triglicèrids) mitjançant una gota de sang extreta d'uns dit i mesurament de components de la dieta mitjançant una mostra d'orina i saliva.

- Anàlisi de la composició corporal a partir d'una densitometria òssia (DEXA). L'acceptació de participar en aquest estudi no obliga a realitzar aquesta prova, fer-la és opcional.

Un cop enllestides les proves, rebràs un informe amb els resultats de les anàlisis de sang, dels mesuraments corporals i de l'anàlisi de la composició corporal a partir del DEXA (si s'ha fet), per tal de conèixer el teu estat general de salut. Les mostres que obtinguem no es faran servir amb cap altra finalitat i seran destruïdes un cop realitzades les anàlisis. Tampoc no farem servir les teves mostres per dur a terme anàlisis genètiques de cap mena.

La teva participació en l'estudi és voluntària i gratuïta. Si desitges participar-hi, necessitarem la teva signatura i la dels teus pares. Si en algun moment desitges abandonar l'estudi, pots fer-ho quan vulguis i sense donar cap tipus d'explicacions.

Tota la informació recollida en aquest estudi serà tractada de forma estrictament confidencial. Això vol dir que ningú no podrà relacionar el teu nom amb els resultats obtinguts. Durant tot el procés, es respectarà la Llei de protecció de dades (Llei orgànica 15/1999 de 13 de desembre) i altres lleis vigents aplicables.

Amb la teva ajuda podrem millorar la salut cardiovascular de la població espanyola.

MOLTES MERCÈS PER LA TEVA COL.LABORACIÓ!

Aquest projecte ha estat aprovat pel Comitè d'Ètica de l'Institut de Salut Carlos III, la Fundació Unió Catalana d'Hospitals i la Comissió de Bioètica de la Universitat de Barcelona.

Barcelona, 10 de gener de 2017

Benvolguts pares i mares,

L'institut del seu fill/a ha estat seleccionat per participar en el Programa SI! de promoció de la salut cardiovascular en adolescents, que duen a terme la Fundació SHE, la Fundació Centre Nacional d'Investigacions Cardiovasculars Carlos III i la Universitat de Barcelona.

Aquest programa s'implantarà **des del setembre de 2017 fins al juny de 2021** a 24 centres d'educació secundària situats a la Comunitat de Madrid i a Catalunya. Vostès tenen l'oportunitat de participar com a voluntaris d'aquest programa.

Objectius del programa

L'adolescència és un període crític durant el qual s'estableixen i reforcen estils de vida (alimentació, activitat física, sedentarisme, etc.) que condicionaran la salut cardiovascular durant l'etapa adulta. Per aquest motiu, l'objectiu d'aquesta iniciativa és **valorar i millorar la salut cardiovascular i els estils de vida** dels adolescents a l'inici de l'educació secundària (inici del primer curs), a la meitat d'aquesta (fi del segons curs) i al final (fi del quart curs). Les proves que farem inclouen:

- Qüestionari d'alimentació (qüestionari de freqüència de consum d'aliments).
- Qüestionari general sobre coneixements, actituds i hàbits sobre els estils de vida.
- Mesuraments antropomètrics als adolescents: pes, estatura, circumferència de cintura i percentatge de greix.
- Ús d'acceleròmetres pel registre objectiu de l'activitat física (petit dispositiu que es porta a la cintura).
- Mesurament de la pressió arterial.
- Mesurament de paràmetres bioquímics (colesterol total, HDL, LDL, triglicèrids i glucosa) mitjançant l'extracció d'una gota de sang capil·lar.
- Determinació de compostos dietètics (polifenols i metabòlits) en una mostra d'orina i saliva.
- Anàlisi de la composició corporal mitjançant una densitometria òssia (DEXA). Aquesta prova serà de caràcter opcional.
- Qüestionari general dirigit a pares i mares sobre aspectes sociodemogràfics i estils de vida (tabaquisme, dieta, activitat física, etc.).

Addicionalment, ens agradaria fer una darrera valoració de seguiment quan els participants compleixin vint anys. Per aquest motiu, li demanem que ens autoritzi a contactar-los de nou en el futur.

Les famílies rebran un correu electrònic amb un enllaç per poder emplenar el qüestionari dirigit a pares i mares. Un cop finalitzada la recollida de dades, es farà arribar a les famílies un informe amb els principals resultats derivats de les anàlisis bioquímiques, antropomètriques, de pressió arterial i de composició corporal obtinguts del DEXA (si s'ha fet) relatius a l'estat de salut del seus fills. Totes les mostres seran analitzades sota els controls de qualitat pertinents. Les mostres serviran per

valorar l'estat de salut general dels participants. Les mostres sobrants no es faran servir a cap altre treball de recerca i seran destruïdes. No es farà cap anàlisi genètica a partir de les mostres obtingudes.

La participació en l'estudi és voluntària i gratuïta:

Ens agradaria deixar clar que tant la participació del/de la seu/va fill/a com la seva són voluntàries i gratuïtes. **Vostè, el/la seu/va fill/a i/o la seva família són lliures d'abandonar l'estudi en qualsevol moment i sense donar explicacions.**

Riscos de participar en el projecte:

La realització de la prova de DEXA suposa una dosi de radiació mínima (gairebé negligible), similar a la rebuda en un viatge d'avió d'una hora de durada o un dia de radiació ambiental natural. La màxima molèstia que pot percebre és una petita punxada al palpís del dit en punxar-li per extraure'n una gota de sang capil·lar.

Confidencialitat

Aquest projecte ha estat aprovat pel Comitè d'Ètica de l'Institut de Salut Carlos III, la Fundació Unió Catalana d'Hospitals i la Comissió de Bioètica de la Universitat de Barcelona i compta amb la col·laboració de la Fundació SHE, la Fundació Centre Nacional d'Investigacions Cardiovasculars Carlos III i la Universitat de Barcelona.

Tota la informació recollida en aquest estudi serà estrictament confidencial. Tota la informació serà tractada de forma anònima mitjançant la seva codificació. Els noms i adreces o altres dades de contacte estaran completament separades de les dades obtingudes a l'estudi, sota clau. Durant tot el procés se seguirà la Llei de protecció de dades (Llei orgànica 15/1999 de 13 de desembre) i altres lleis vigents aplicables.

Els resultats anònims derivats de l'estudi seran de gran ajuda per als responsables d'elaborar les polítiques de salut pública en el futur, amb l'objectiu de millorar la salut cardiovascular de la població espanyola.

MOLTES MERCÈS PER LA SEVA ATENCIÓ I COL.LABORACIÓ

Detalls de contacte

Per a qualsevol consulta pot comunicar-se amb el nostre equip mitjançant el **correu electrònic** estudio@fundacionshe.org o bé trucant al **telèfon** 93 218 54 44.

FULL DE CONSENTIMENT INFORMAT-PROGRAMA SI! SECUNDÀRIA

TÍTOL DEL PROJECTE: Intervenció educativa en escolars per fer front a l'obesitat i promoure la salut cardiovascular en adolescents espanyols: un assaig controlat i aleatoritzat per grups.

Centres participants: Campus de l'Alimentació de Torribera. Departament de Nutrició, Bromatologia i Gastronomia de la Facultat de Farmàcia (Universitat de Barcelona); Centre Nacional d'Investigacions Cardiovasculars Carlos III (CNIC); Fundació SHE (Foundation for Science Health and Education).

Investigadora principal: Dra. Rosa M. Lamuela

Jo (nom i cognoms) ………

autoritzo el/la meu/va fill/a (nom i cognoms) ……………………………………………...………….......................de

l'institut……………………………………………………………………………………………………..........….a participar en l'estudi.

- He llegit el full informatiu que acompanya aquest consentiment.
- He pogut fer preguntes sobre l'estudi.
- He rebut suficient informació sobre l'estudi.
- He estat informat/ada que les dades personals recollides en aquest estudi es tractaran de forma estrictament confidencial, segons la Llei orgànica 15/1999 de Protecció de dades personals i la seva normativa de desenvolupament.
- He estat informat/ada que les dades obtingudes només es faran servir per a la finalitat específica d'aquest estudi.

Comprenc que la participació del/de la meu/va fill/a i de la seva família és voluntària. Comprenc que tant el/la meu/va fill/a com la seva família poden abandonar l'estudi quan vulguin sense haver de donar explicacions. A més a més, m'han facilitat les dades de contacte dels investigadors de l'estudi per tal de poder solucionar qualsevol dubte que pogués sorgir referent a aquest estudi.

Donem lliurement la nostra conformitat per participar en l'estudi:

☐ En la seva totalitat ☐ Sense densitometria òssia (DEXA)

Correu electrònic: ……………………………………………………… Telèfon: ………………………./…………………………

____________________	________________________	________________________
(Data)	(Signatura de l'adolescent)	(Signatura de la mare, pare o tutor legal)*

____________________	____________________________
(Data)	(Signatura de l'investigador)

☐ Autoritzo a ser contactat/ada quan el/la meu/va fill/a tingui 20 anys mitjançant el correu electrònic i el telèfon anteriorment indicats.

** Si la tutela és compartida, hauran de signar ambdós progenitors (si només signés un, cal adjuntar una delegació escrita del progenitor absent al present).*

Còpia per al pare, mare o tutor.

Aquest projecte ha estat aprovat pel Comitè d'Ètica de l'Institut de Salut Carlos III, la Fundació Unió Catalana d'Hospitals i la Comissió de Bioètica de la Universitat de Barcelona.

Programa de Salut Integral

FULL DE CONSENTIMENT INFORMAT-PROGRAMA SI! SECUNDÀRIA

TÍTOL DEL PROJECTE: Intervenció educativa en escolars per fer front a l'obesitat i promoure la salut cardiovascular en adolescents espanyols: un assaig controlat i aleatoritzat per grups.

Centres participants: Campus de l'Alimentació de Torribera. Departament de Nutrició, Bromatologia i Gastronomia de la Facultat de Farmàcia (Universitat de Barcelona); Centre Nacional d'Investigacions Cardiovasculars Carlos III (CNIC); Fundació SHE (Foundation for Science Health and Education).

Investigadora principal: Dra. Rosa M. Lamuela

Jo (nom i cognoms) ………

autoritzo el/la meu/va fill/a (nom i cognoms) ………………………………………………………………………………de

l'institut……………………………………………………………………………………………………a participar en l'estudi.

- He llegit el full informatiu que acompanya aquest consentiment.
- He pogut fer preguntes sobre l'estudi.
- He rebut suficient informació sobre l'estudi.
- He estat informat/ada que les dades personals recollides en aquest estudi es tractaran de forma estrictament confidencial, segons la Llei orgànica 15/1999 de Protecció de dades personals i la seva normativa de desenvolupament.
- He estat informat/ada que les dades obtingudes només es faran servir per a la finalitat específica d'aquest estudi.

Comprenc que la participació del/de la meu/va fill/a i de la seva família és voluntària. Comprenc que tant el/la meu/va fill/a com la seva família poden abandonar l'estudi quan vulguin sense haver de donar explicacions. A més a més, m'han facilitat les dades de contacte dels investigadors de l'estudi per tal de poder solucionar qualsevol dubte que pogués sorgir referent a aquest estudi.

Donem lliurement la nostra conformitat per participar en l'estudi:

☐ En la seva totalitat ☐ Sense densitometria òssia (DEXA)

Correu electrònic: ……………………………………………………… Telèfon: ………………………/…………………………

___________________	________________________	_______________________
(Data)	(Signatura de l'adolescent)	(Signatura de la mare, pare o tutor legal)*

_____________________	_____________________________
(Data)	(Signatura de l'investigador)

☐ Autoritzo a ser contactat/ada quan el/la meu/va fill/a tingui 20 anys mitjançant el correu electrònic i el telèfon anteriorment indicats.

** Si la tutela és compartida, hauran de signar ambdós progenitors (si només signés un, cal adjuntar una delegació escrita del progenitor absent al present).*

Còpia per a l'investigador principal.

Aquest projecte ha estat aprovat pel Comitè d'Ètica de l'Institut de Salut Carlos III, la Fundació Unió Catalana d'Hospitals i la Comissió de Bioètica de la Universitat de Barcelona.

Foundation for Science, Health and Education
Fundación Centro Nacional de Investigaciones Cardiovasculares Carlos III
UNIVERSITAT DE BARCELONA

COMPROMÍS DE CONFIDENCIALITAT

Dra. Rosa Maria Lamuela Raventós, amb DNI/CIF nº 46121028-W, com investigadora principal de l'estudi "School-based educational intervention to face obesity and promote Cardiovascular health among Spanish adolescents: a cluster-randomized controlled trial." dut a terme a la Universitat de Barcelona

MANIFESTA

1.- que s'observarà la confidencialitat de les dades que s'obtinguin en el projecte.

2.- que les dades personals dels subjectes participants no seran conegudes pels investigadors del projecte.

I com a prova de la conformitat, signa el present Compromís

Barcelona, 18 de març de 2016

Signat Dra Rosa Lamuela

COMPROMÍS DE NO TRASPÀS DE MOSTRES A ALTRES PROJECTES

Dra. Rosa Maria Lamuela Raventós, amb DNI/CIF nº 46121028-W, com investigadora principal de l'estudi "School-based educational intervention to face obesity and promote Cardiovascular health among Spanish adolescents: a cluster-randomized controlled trial." dut a terme a la Universitat de Barcelona

MANIFESTA

1.- Que no utilitzarà les mostres per altres estudis diferents als d'aquests projectes i a no traspassar les mostres a altres possibles projectes o equips d'investigació. En aquest supòsit, es compromet a sol·licitar abans el corresponent informe de la Comissió.

I com a prova de la conformitat, signa el present Compromís de no traspàs de mostres a altres projectes.

Barcelona, 18 de març de 2016

Signat Dra Rosa Lamuela

COMPROMÍS DE COMPENSACIÓ ECONÒMICA

Dra. Rosa Maria Lamuela Raventós, amb DNI/CIF nº 46121028-W, com investigadora principal de l'estudi "School-based educational intervention to face obesity and promote Cardiovascular health among Spanish adolescents: a cluster-randomized controlled trial." dut a terme a la Universitat de Barcelona

MANIFESTA

1.- Que no es preveuen compensacions econòmiques per a l'investigador principal ni per als subjectes que hi participen.

I com a prova de la conformitat, signa el present

Barcelona, 18 de març de 2016

Signat Dra Rosa Lamuela

COMPROMÍS

Dra. Rosa Maria Lamuela Raventós, amb DNI/CIF nº 46121028-W, com investigadora principal de l'estudi "School-based educational intervention to face obesity and promote Cardiovascular health among Spanish adolescents: a cluster-randomized controlled trial." dut a terme a la Universitat de Barcelona

MANIFESTA

1.- Que els estudiants participants com a voluntaris de l'estudi no mantenen cap relació acadèmica amb l'investigador principal durant el període de realització del projecte.

I com a prova de la conformitat, signa el present Compromís

Barcelona, 18 de març de 2016

Signat Dra Rosa Lamuela
