# Supplementary material for: School-Based Cardiovascular Health Promotion in Adolescents: A Cluster Randomized Clinical Trial
Source: JAMA Cardiol. 2023 Aug 2;8(9):816–24. doi: 10.1001/jamacardio.2023.2231 (PMC10398546; doi:10.1001/jamacardio.2023.2231)
Supplement: Supplement 2. — eMethods. Detailed Methods eTable 1. Template for Intervention Description and Replication (TIDieR) Checklist eTable 2. Objectives and Timing: Long-term and Short-term Intervention eTable 3. Ideal Cardiovascular Health Metrics for Children and Adolescents as Defined by the American Heart Association and as Used in this Study eTable 4. Baseline Characteristics of Adolescents Included and Excluded From the Primary Analysis (Complete-Case Intention-to-Treat Analysis) at 2-Year Follow-up in the SI! Program for Secondary School Trial eTable 5. Baseline Characteristics of Adolescents Included and Excluded From the Primary Analysis (Complete-Case Intention-to-Treat Analysis) at 4-Year Follow-up in the SI! Program for Secondary School Trial eTable 6. Overall CVH Change Within and Between Intervention Groups for All Randomized Enrolled Participants eTable 7. Changes in the Continuous Metrics Comprising the CVH Score at 2-Year Follow-up, Within and Between Randomization Groups eTable 8. Changes in the Continuous Metrics Comprising the CVH Score at 4-Year Follow-up, Within and Between Randomization Groups eTable 9. Adjusted Changes in the Overall CVH Score and Individual CVH Metrics at 2-Year Follow-up, Within and Between Randomization Groups eTable 10. Adjusted Changes in the Overall CVH Score and Individual CVH Metrics at 4-Year Follow-up, Within and Between Randomization Groups eTable 11. Main Qualitative Results From Focus Groups on Teachers and Students eTable 12. Overall CVH Change Within and Between Intervention Groups Excluding the Blood Glucose Metric eFigure 1. Examples of Intervention Material eFigure 2. Extended Study Flowchart eFigure 3. Change in the Overall CVH Score at 2-Year Follow-up According to Sociodemographic Characteristics eFigure 4. Change in the Overall CVH Score at 4-Year Follow-up According to Sociodemographic Characteristics eReferences [file jamacardiol-e232231-s002.pdf]

Supplementary Online Content

Santos-Beneit G, Fernández-Alvira JM, Tresserra-Rimbau A, et al. School-based cardiovascular health promotion in adolescents: a cluster randomized clinical trial. *JAMA Cardiol*. Published online August 2, 2023. doi:10.1001/jamacardio.2023.2231

eMethods. Detailed Methods

eTable 1. Template for Intervention Description and Replication (TIDieR) Checklist

eTable 2. Objectives and Timing: Long-term and Short-term Intervention

eTable 3. Ideal Cardiovascular Health Metrics for Children and Adolescents as Defined by the American Heart Association and as Used in this Study

eTable 4. Baseline Characteristics of Adolescents Included and Excluded From the Primary Analysis (Complete-Case Intention-to-Treat Analysis) at 2-Year Follow-up in the SI! Program for Secondary School Trial

eTable 5. Baseline Characteristics of Adolescents Included and Excluded From the Primary Analysis (Complete-Case Intention-to-Treat Analysis) at 4-Year Follow-up in the SI! Program for Secondary School Trial

eTable 6. Overall CVH Change Within and Between Intervention Groups for All Randomized Enrolled Participants

eTable 7. Changes in the Continuous Metrics Comprising the CVH Score at 2-Year Follow-up, Within and Between Randomization Groups

eTable 8. Changes in the Continuous Metrics Comprising the CVH Score at 4-Year Follow-up, Within and Between Randomization Groups

eTable 9. Adjusted Changes in the Overall CVH Score and Individual CVH Metrics at 2-Year Follow-up, Within and Between Randomization Groups

eTable 10. Adjusted Changes in the Overall CVH Score and Individual CVH Metrics at 4-Year Follow-up, Within and Between Randomization Groups

eTable 11. Main Qualitative Results From Focus Groups on Teachers and Students

eTable 12. Overall CVH Change Within and Between Intervention Groups Excluding the Blood Glucose Metric

eFigure 1. Examples of Intervention Material

eFigure 2. Extended Study Flowchart

eFigure 3. Change in the Overall CVH Score at 2-Year Follow-up According to Sociodemographic Characteristics

eFigure 4. Change in the Overall CVH Score at 4-Year Follow-up According to Sociodemographic Characteristics

eReferences

This supplementary material has been provided by the authors to give readers additional information about their work.

© 2023 Santos-Beneit G et al. *JAMA Cardiology*.

eMethods. Detailed Methods

Description of the intervention

Rationale

The SI! Program (*Salud Integral*) for Secondary Schools was developed to promote cardiovascular health (CVH) and long-lasting healthy habits among adolescents and thereby reduce the risk of future cardiovascular disease. The intervention seeks to promote CVH by focusing on the development of appropriate attitudes and knowledge towards healthy lifestyle habits according to the transtheoretical model of behavioral change.[1] Adolescents are particularly sensitive to environmental influences, highlighting the importance of adopting a social determinant approach to understanding adolescent health and well-being, as well as a positive youth-development approach which focuses on the adolescents' assets and developmental strengths.[2] The following health objectives were defined according to risk factors by a multidisciplinary team of experts:[3] i) acquire healthy eating habits, ii) acquire healthy physical activity (PA) habits, and iii) acquire protective skills to avoid smoking and substance abuse. Subsequently, adolescents' risk-taking behavior and beliefs were identified,[4-12] and educational goals were set to promote alternative options to this risk-taking behavior in adolescents[5,6,13-20] (**eTable 3**). Based on the Attitude, Social influence and-self-Efficacy (ASE) model of behavioral change,[21] previously used for similar school-based interventions,[13-20] the first goal was to promote a positive attitude towards healthy behavior and to improve self-efficacy to carry out the healthy behavior.[22] To promote a positive attitude, the beliefs associated with the most relevant risk behavior are included as content in classroom activities. According to the socio-ecological model,[23] healthy behavior is determined not only by personal attitude but also by the individual's social, physical, and organizational environment, and evidence suggests that the most effective interventions are those that mobilize the whole school as an organization.[24] Thus, specific educational objectives were designed to be imparted through classroom activities and reinforced by the student's immediate environment: teachers, family, and the school environment.

Materials and procedures

In each school year all the Program health objectives were organized into the following teaching units:
1. *Healthy eating:* Students were taught how to design balanced meals, taking into consideration ingredients and portion size, alternatives to sugar-sweetened drinks, salt and fat content, nutritional labeling, and the detection of emotional eating, among other factors.
2. *PA:* Students were encouraged to become familiar with different forms of PA, to put these into practice, to plan daily training sessions to improve CVH, and recognize the importance of rest. Students were invited to discover their talents and abilities during sports activities and play, and to participate in aerobic activities to improve CVH and explore active and healthy leisure options.
3. *Protective factors against smoking and substance abuse:* Adolescents worked mainly on self-esteem, self-knowledge, and skills in rejecting smoking and substance abuse. In particular, they were encouraged to identify their talents and hobbies; learn strategies for saying "no", especially to non-cardio-leisure activities; apply techniques to feel better in situations that provoke stress, anxiety, or insecurity; and define ideal and personal healthy leisure activities.

Body acceptance was integrated into all three teaching units. The activities encouraged critical analysis of advertisements and beauty stereotypes, as well as acceptance of individual physical attributes and personal idiosyncrasies.

Each teaching unit consisted of classroom sessions including sub-activities (initial, development, and conclusion). At the beginning and end of each teaching unit, participants were shown an animated motivational video aimed at interconnecting the units and generating a positive attitude toward the corresponding health objective. Some examples of intervention material can be found in **eFigure 1.** At the end of each main activity, students were asked to complete a diary, answering questions that promoted both self-reflection on personal goals and a description of the key ideas of the unit.

In addition, throughout the intervention, students were encouraged to undertake PA after school through gamification activities involving motivational messages and teacher-determined rewards for daily PA performed after school and during classroom activities.[25] Examples of rewards introduced by the schools included pieces of fruit, sports equipment (such as table tennis bats), diplomas, tickets to sporting events, field trips, and the choice of special activities in a particular session.

© 2023 Santos-Beneit G et al. *JAMA Cardiology*.

At the end of the last school year of the intervention, students completed a synthesis activity for each health goal. Students used a decision tree with a guided decision-making process to consolidate healthy behaviors and plan new healthy habits for their daily life. Students also wrote a letter of intention for their own future reference.

Families received three newsletters related to the contents of each teaching unit in each school year (except the 4th grade) (**eFigure 1**). Schools distributed the newsletters by email or as printed documents at the end of the relevant teaching unit. Each newsletter consisted of i) an introduction to the promoted healthy habits; ii) an explanation of the importance of these habits for the corresponding age group; and iii) evidence-based key messages containing information and strategies for use at home. The newsletters also included links to resources for further information.

Lastly, the SHE (Science, Health and Education) Foundation prepared a document with 10 recommendations written as key messages for teachers and students, with the aim of encouraging their participation in a school-wide healthy environment. These recommendations included improvements to the physical environment (e.g., installing bike racks for bike parking), as well as the establishment of practices or resources that impact health habits (e.g., access to healthy foods for students and staff; conflict resolution strategies; and the provision of opportunities for PA before, during, and after school). An annual Health Day was encouraged as a fun way to enhance health awareness among students, staff, and families.

Study setting and modes of delivery

The educational intervention was based primarily on classroom activities, which could be printed or projected on a screen. Some classroom activities included individual and group activities, and others included interactive computer mini-games. Students could access these activities and other complementary resources through an interactive website. For each grade, a specific age-related motivational theme was devised, while incorporating shared methodological features. The content was presented to students as a virtual journey on which they met different characters who guided them through questions, reflections, information, and health tips. Access to the Program website was restricted to students whose parents/caregivers had signed an informed consent form. Registered students accessed the virtual journey through a personalized avatar and recorded their daily PA at home in order to advance on the journey and arrive together with their classmates at the destination. Motivational messages were sent to students by the website according to the amount of recorded at-home PA or by the teacher (to all registered students in the class or to individual students) according to their participation in classroom activities.

Intervention provider

School principals signed an agreement to register on the online application so that teachers could access the intervention materials and gamification activities, including instructions and specific guides for each activity. All teaching activities were delivered in the school classroom by the regular teachers. The teaching units were designed to be interdisciplinary, and some of them fitted within the school curriculum (mainly Physical Education, Sciences, and Tutoring), making it easier for teachers to include them in their program. The SHE Foundation provided teachers with training on how to implement the program, including content on the promotion of CVH, as well as technical and logistical aspects. The training lasted 30 hours, including 10 hours of face-to-face sessions and 20 hours of individual work in the school. This training was accredited by the corresponding regional public educational authority. Within each school, at least one staff member acted as a Health Coordinator. The training was mandatory for the Health Coordinator and highly recommended for all teachers who implemented the program. In addition, Health Coordinators and school principals received follow-up (FU) e-mails to guide them in developing the intervention and to motivate them. The Health Coordinator and school leadership team were also in charge of planning and promoting the Health Day and monitoring the achievement of the school environment goals.

Duration and intensity

eTable 7 shows the objectives for each school year and the timing for each type of intervention. From the 1st through the 4th grades, at least 4 hours per school year were dedicated to each long-term intervention (LTI) objective and 6 hours to each short-term intervention (STI) objective. The planned number of hours dedicated to classroom activities were 12 hours per school year in the LTI and 18 hours in the STI. In addition, in the 2nd year of the STI, an additional 2 hours were dedicated to the synthesis activity. In the LTI, the final activity included a "Letter to my future self", in which students wrote about habits they had consolidated and those they would like to have in the future, how they were going to achieve their goals, and the positive influences they would like to bring to their environment (family, peers, school, clubs, etc.). During the final school year of the LTI, at least 9 hours were dedicated to the synthesis activity (3 hours for each health objective) plus at least 3 hours of personal work to writing the letter. In addition, during the

© 2023 Santos-Beneit G et al. *JAMA Cardiology*.

last school year of the LTI, students could voluntarily opt to carry out a health research project, either individually or as a group. The project consisted of putting into practice the knowledge acquired throughout the intervention to improve the CVH habits of fellow students, a friend or family member, or any other specific group of people. Projects were supervised by teachers and could be submitted for a prize. Students and teachers involved in the three best projects were awarded diplomas, the school received a cash prize, and students received a commemorative gift card.

Tailoring

The materials (online application, sessions, family newsletter, school environment recommendations) were available in two languages (Spanish/Catalan).

The use of the gamification activities was encouraged; however, to take account of technological issues[25] teachers were allowed to implement content exclusively through classroom activities and resources provided by email or USB. In addition, although schools received recommendations and guidelines on setting rewards for participation, teachers had the final decision on how to motivate and assess student participation.

Teacher training was also adapted to allow it to be carried out in person or online, or even as one-to-one training if a Health Coordinator could not attend the group training.

At any time, teachers were able to discuss specific issues with the SHE Foundation Coordinators by email, phone, or through face-to-face or online meetings.

Intervention adherence and modifications

The following actions were taken to ensure faithful implementation and to assess intervention adherence:

(i) Each teacher completed a FU record of the activities (length of each session, student enjoyment of each session, and identified difficulties or barriers).

(ii) The Health Coordinator at each center completed an evaluation survey at the end of the school year, specifying and rating the content and the teaching resources, as well as any barriers, and suggestions for improvement.

(iii) The school leadership team completed a survey on the school environment.

(iv) The corresponding coordinator of the SHE Foundation completed annual reports on the level of program implementation at each school, including the number of teachers trained, communication with the Health Coordinator, and the use of interactive platform resources.

In the 1st and 2nd grades, LTI schools completed ~90% of the activities, whereas STI schools completed ~75%. During the COVID-19 pandemic in 2020, 10 sessions were available via remote learning (12 hours), and an average of 6 sessions were completed (~7 hours). During lockdown, the SHE Foundation provided extra online resources to promote healthy habits, mostly related to psychological management, but also including tips on healthy eating and indoor exercise. The imposed hybrid learning schedule during pandemic also affected the 4th grade due to associated work overload, self-quarantine periods, and teacher and student burnout. COVID-19 restrictions also affected the school environment, especially the Health Day.

The main limitation of the intervention was technological issues related to gamification, so this motivating resource was not integrated into daily classroom and after-school activities. Moreover, not all teachers involved in the program had a positive attitude towards digital technologies. Both of these factors had a crucial influence on the success of gamification activities. When gamification activities were not implemented, teachers were asked to print or project the classroom activities to achieve the required minimum content.

Some activities, such as those related to protective factors or the PA component, were not implemented throughout consecutive school years as initially intended, diminishing the potential for message reinforcement by multiple teachers.

© 2023 Santos-Beneit G et al. *JAMA Cardiology*.

Data collection

Cardiovascular health (CVH) metrics

Smoking status

Smoking status was assessed with a standard questionnaire.[26] Adolescents who reported never having smoked tobacco products (cigarettes, e-cigarettes, or hookah) were categorized as having an ideal smoking status; all other individuals were classified as having a poor smoking status. For adolescents who did not report a smoking habit at FU, smoking status was categorized as poor only if it had been categorized as such in previous measurements (n= 5, 0.4% at 4-year FU).

Body mass index

Body weight was measured with an OMRON BF511 electronic scale and height with a Seca 213 portable stadiometer, with the participant wearing light clothes and no shoes. Body mass index (BMI) was calculated as body weight divided by height squared (kg/m^2). Age- and sex-adjusted BMI percentiles were calculated according to Centers for Disease Control standards,[27] with ideal, intermediate, and poor BMI defined as <85th percentile, 85-95th percentile, and >95th percentile, respectively.

Physical activity

Type, intensity, and amount of PA was calculated with an Actigraph wGT3X-BT accelerometer that the participant wore for 7 consecutive days. Accelerometer data were considered valid for those individuals with at least 4 days with 600 valid minutes per day during daytime.[28] Chandler (2016)[29] cut-off points were applied for the calculation of time spent in different physical activity intensities. For those with no accelerometer data available (n=85, 6.4% at baseline; n=187, 14.1% at 2-year FU; n=227, 17.1% at 4-year FU), the validated QAPACE survey (*Quantification de L'Activité Physique en Altitude chez les Enfants*) was used to quantify individual PA.[30] Ideal status for this individual health metric was defined as ≥60 min/day moderate-to-vigorous activity, intermediate status as 1 to <60 min/day moderate-to-vigorous activity, and poor status as reported no moderate-to-vigorous PA (0 min/day).

Diet

Diet was assessed with the validated Children's Eating Habits Questionnaire (CEHQ).[31-33] If the CEHQ was not available, diet was assessed with an updated version of the validated 157-item semi-quantitative food frequency questionnaire (FFQ)[34] (n=4, 0.3% at baseline; n=7, 0.5% at 2-year FU; n=12, 0.9% at 4-year FU). The healthy diet score used for dietary profiling included thresholds for the intake of fruit and vegetables (≥4.5 servings/day), fish (≥2 servings/week), fiber-rich whole grains (≥1 servings/day), and sugar-sweetened beverages (≤1 L/week). Adolescents who met all four healthy diet criteria were classified as having an ideal diet, while those meeting 2-3 or only 0-1 of the criteria were classified as having an intermediate or poor diet, respectively.

Blood pressure

Blood pressure (BP) was measured twice with an OMRON M6 monitor at 2-3 minute intervals.[35] When there was a difference between measurements of >10 mmHg for systolic BP or >5 mmHg for diastolic BP, additional measurements were taken. For analysis, we used the pair with the minimum systolic BP, or minimum diastolic BP in case of a tie. BP percentiles and stages were calculated according to BP reference data from the American Academy of Pediatrics.[36] Ideal, intermediate, and poor BP were defined as <90th percentile, 90-95th percentile, and >95th percentile, respectively.

Blood glucose and total cholesterol levels

Fasting blood glucose and total cholesterol (TC) were measured using a CardioCheck Plus device and PTS-Panels test strips[37] in capillary blood sampled with a lancet. Ideal TC was defined as TC <170 mg/dL, intermediate TC 170-199 mg/dL, and poor TC ≥200 mg/dL. Ideal blood glucose was <100 mg/dL, intermediate glucose 100–125 mg/dL, and poor glucose ≥126 mg/dL.

Covariates

Highest self-reported parental educational level was categorized according to the International Standard Classification of Education (ISCED): low (no studies, primary studies, or secondary studies; 0 to 3 ISCED score), medium (post-secondary non-tertiary education or short-cycle tertiary education; 4 to 5 ISCED score), and high (university studies; 6 to 8 ISCED score).[38] Self-reported household income was defined according to the most recently published Spanish average annual household income at the time of each data collection: at baseline the 2016 value was used (26,730€); at 2-year FU the value from 2017 (27,558€); and at 4-year FU the value from 2019 (29,132€).[39] Household income information was collected and classified into three categories: low, average and high. A migrant background was assumed if at least one

© 2023 Santos-Beneit G et al. *JAMA Cardiology*.

parent/caregiver was born outside Spain. The analysis considered the covariate data collected at baseline. If this information was unavailable at baseline but was collected at any succeeding FU, the analysis considered the earliest reported information (<5% cases).

Data analysis

Multiple imputation procedure

A sensitivity analysis (**eFigure 2**) was performed after multiple imputation using multivariate normal distribution (Markov Chain Monte Carlo procedures), implemented by the command *mi*. The change from baseline in a participant's overall CVH score was the set as the imputed dependent variable. Complete cases represented 91% and 86% (1212 and 1095 out of 1326 randomized enrolled participants) at 2-year and 4-year FU, respectively. Missing data were assumed to be missing at random. The following variables were included as auxiliary variables: age (continuous variable), gender (binary variable), and overall CVH score at baseline (continuous variable). Complete covariate information was available for most randomized enrolled participants (1324, 99.8%), and auxiliary variables were imputed as needed. The number of imputations was set at 50. A ridge prior distribution with 10 degrees of freedom was used to stabilize inferences.[40] A random-number seed was set to ensure reproducibility of the imputed values. Estimations on the imputed data were run with the "mi estimate" command, which adjusts coefficients and standard errors for the variability between imputations according to the combination rules by Rubin [41] Multilevel linear mixed-effects models that account for the hierarchical cluster randomized design were used to test for the adjusted intervention effect. Fixed effects were the corresponding baseline score and treatment group. Region and schools were handled as random effects. Diagnostic checks of the imputation model were obtained using the *vartable* and *dftable* options of the "mi estimate" command.
All analyses were performed using STATA version 15 (StataCorp, College Station, Texas).

© 2023 Santos-Beneit G et al. *JAMA Cardiology*.

eTable 1. Template for Intervention Description and Replication (TIDieR) checklist

Item number	Item	Where located<br>Primary paper<br>(page or appendix number)
	**BRIEF NAME**	1, 2, S2
**1.**	Provide the name or a phrase that describes the intervention.	
	**WHY**	
**2.**	Describe any rationale, theory, or goal of the elements essential to the intervention.	3, S2-S4
	**WHAT**	
**3.**	Materials: Describe any physical or informational materials used in the intervention, including those provided to participants or used in intervention delivery or in training of intervention providers. Provide information on where the materials can be accessed (e.g. online appendix, URL).	3, S4, eFigure 1
**4.**	Procedures: Describe each of the procedures, activities, and/or processes used in the intervention, including any enabling or support activities.	S2-S4, eTable 3, eFigure 1
	**WHO PROVIDED**	
**5.**	For each category of intervention provider (e.g. psychologist, nursing assistant), describe their expertise, background and any specific training given.	3, S3
	**HOW**	
**6.**	Describe the modes of delivery (e.g. face-to-face or by some other mechanism, such as internet or telephone) of the intervention and whether it was provided individually or in a group.	3, S3-S4
	**WHERE**	
**7.**	Describe the type(s) of location(s) where the intervention occurred, including any necessary infrastructure or relevant features.	1, 2, S3
	**WHEN and HOW MUCH**	
**8.**	Describe the number of times the intervention was delivered and over what period including the number of sessions, their schedule, and their duration, intensity or dose.	3, S3-S4, eTable 2
	**TAILORING**	
**9.**	If the intervention was planned to be personalized or adapted, then describe what, why, when, and how.	8, S4
	**MODIFICATIONS**	
**10.**[‡]	If the intervention was modified during the course of the study, describe the changes (what, why, when, and how).	S4
	**HOW WELL**	
**11.**	Planned: If intervention adherence or fidelity was assessed, describe how and by whom, and if any strategies were used to maintain or improve fidelity, describe them.	S4, eTable 2
**12.**	Actual: If intervention adherence or fidelity was assessed, describe the extent to which the intervention was delivered as planned.	S4

Checklist for interventions reporting[43]

© 2023 Santos-Beneit G et al. *JAMA Cardiology*.

eTable 2. Objectives and timing: long-term and short-term intervention

**Grade 1 (12-13 years-old) - MOTIVATIONAL AXIS: Health as an individual responsibility**	
**<u>Physical activity</u>**<br><br>**Teaching Unit 1.**	**4 sessions (4 hours)**<br>- Develop a positive attitude towards healthy recommendations for physical activity and sport.<br>- Practice appropriate activity and rest patterns for the heart on a regular basis.<br>- Develop responsible behavior and actions to maintain a healthy body.<br>*Extra content in short-term intervention:*<br>- Develop the behavior and routines necessary for the proper functioning of the body and heart.
**<u>Healthy eating</u>**<br><br>**Teaching Unit 2.**	**3 sessions (4 hours)**<br>- Acquire knowledge about the Mediterranean diet and its benefits.<br>- Acquire self-efficacy to have five meals a day and drink water regularly.<br>*Extra content in short-term intervention:*<br>- Acquire responsibility for health, after learning about the effects of overweight/obesity.<br>- Acquire self-efficacy to have balanced, varied and moderate intake patterns.
**<u>Protective factors</u>**<br><br>**Teaching Unit 3.**	**4 sessions (4 hours)**<br>- Develop positive attitudes towards healthy leisure activities.<br>- Develop a negative attitude towards tobacco consumption (know the relationship between body and tobacco).<br>- Establish satisfactory and assertive social relationships.<br>- Strengthen decision-making and problem-solving skills.<br>- Strengthen self-esteem.<br>*Extra content in short-term intervention:*<br>- Strengthen self-knowledge of talents and achievements.
**Grade 2 (13-14 years-old) - MOTIVATIONAL AXIS: Acceptance and care of one's own body**	
**<u>Healthy eating</u>**<br><br>**Teaching Unit 1.**	**3 sessions (4 hours)**<br>- Acquire self-efficacy to have balanced, varied, and moderate eating patterns.<br>- Develop media literacy strategies for the critical analysis of the ideal body stereotype (knowledge of food myths).<br>- Knowledge about the body mass index is and its calculation, body composition, and blood pressure.

© 2023 Santos-Beneit G et al. *JAMA Cardiology*.

	- Strengthen skills of acceptance of one's own body.<br>*Extra content in short-term intervention:*<br>- Acquire knowledge about what trans fatty acids and cholesterol are and their effects on cardiovascular health.<br>- Develop skills for managing stress and maintaining a positive mood by avoiding emotional eating.
**Physical activity**<br><br>**Teaching Unit 2.**	**3 sessions (4 hours)**<br>- Practice healthy leisure activities of sport and physical activity regularly, limiting screen time.<br>- Develop responsible behavior and actions to maintain a healthy body.<br>*Extra content in short-term intervention:*<br>- Identify and plan the basic characteristics of a training activity.
**Protective factors**<br><br>**Teaching Unit 3.**	**4 sessions (4 hours)**<br>- Develop a positive attitude towards healthy leisure (vs. consumption of toxics).<br>- Develop a negative attitude towards smoking (knowledge of the relationship between smoking and blood pressure).<br>- Strengthen self-concept and personal skills for the detection and overcoming of peer pressure in relation to toxic consumption.<br>- Develop strategies for coping with negative emotions and encourage self-control.<br>*Extra content in short-term intervention:*<br>- Develop a negative attitude towards the consumption of alcohol (identifying risk consumption of alcohol) and other addictive substances.<br>- Value the social, emotional, and mental benefits of practicing healthy leisure activities.<br>- Strengthen personal skills for the detection and overcoming of peer pressure in relation to the consumption smoking and substance abuse.
**Final session (2 hours) only for short-term intervention:**<br>Practical application of the knowledge acquired in the two school years.	
**GRADE 3 (14-15 years-old) - MOTIVATIONAL AXIS: The heart and life**	
**Protective factors**<br><br>**Teaching Unit 1.**	**4 sessions (4 hours)**<br>- Strengthen self-knowledge of talents and achievements.<br>- Develop a negative attitude towards the consumption of alcohol (identifying risk consumption of alcohol) and other addictive substances.<br>- Strengthen personal skills to detect and overcome peer pressure in relation to the consumption of smoking and substance abuse.<br>- Value the social, emotional, and mental benefits of practicing healthy leisure activities.

© 2023 Santos-Beneit G et al. *JAMA Cardiology*.

**<u>Physical activity</u>**<br><br>**Teaching Unit 2.**	**3 sessions (4 hours)**<br>- Know the behavior and routines necessary for the proper functioning of the body and heart.<br>- Identify the most satisfactory physical or sports activities.<br>- Identify and plan the basic characteristics of a workout.
**<u>Healthy eating</u>**<br><br>**Teaching Unit 3.**	**3 sessions (4 hours)**<br>- Acquire self-efficacy to have balanced, varied, and moderate eating patterns.<br>- Acquire responsibility for health, after learning about the effects of overweight/obesity.<br>- Develop skills for stress management and maintenance of a positive mood by avoiding emotional eating.<br>- Acquire knowledge about the Mediterranean diet and its benefits.<br>- Acquire knowledge about what trans fatty acids and cholesterol are and their effects on cardiovascular health.
**GRADE 4 (15-16 years-old) - MOTIVATIONAL AXIS: Healthy leisure**	
**(12 hours)**<br>**Final session:**<br>- Apply and become aware of the healthy habits acquired in the three previous courses.<br>- Facilitate decision making towards healthy behaviors.<br>**Contest:**<br>- Develop a healthy lifestyle project.	

© 2023 Santos-Beneit G et al. *JAMA Cardiology*.

eTable 3. Ideal cardiovascular health metrics for children and adolescents as defined by the American Heart Association and as used in this study

	**Poor**		**Intermediate**		**Ideal**	
**Metric**	**AHA criteria**	**Study criteria**	**AHA criteria**	**Study criteria**	**AHA criteria**	**Study criteria**
Smoking status	Tried >30 days ago	Tried ever before	---	---	Never tried; never smoked whole cigarette	Never tried; never smoked whole cigarette
Body mass index	>95th percentile	>95th percentile	85th-95th percentile	85th-95th percentile	<85th percentile	<85th percentile
Physical activity level	None	None	>0 and <60 min/day moderate or vigorous activity every day	>0 and <60 min/day moderate or vigorous activity every day	≥60 min/day moderate or vigorous activity every day	≥60 min/day moderate or vigorous activity every day
Healthy Diet Score[a]	0-1 components	0-1 components	2-3 components	2-3 components	4-5 components	4 components
Total cholesterol	≥200 mg/dL	≥200 mg/dL	170-199 mg/dL	170-199 mg/dL	<170 mg/dL	<170 mg/dL
Blood pressure	>95th percentile	>95th percentile	90-95th percentile	90-95th percentile	<90th percentile	<90th percentile
Blood glucose	≥126 mg/dL	≥126 mg/dL	100-125 mg/dL	100-125 mg/dL	<100 mg/dL	<100 mg/dL

AHA criteria, cardiovascular health components defined according to the American Heart Association guidelines [44]

[a] The Healthy Diet Score is based on adherence to the following dietary recommendations: fruits and vegetables, ≥4.5 cups per day; fish, 2 or more 3.5-oz servings per week; sodium, ≤1500 mg/d; sugar-sweetened beverages, ≤450 kcal (36 oz) per week; and whole grains, ≥3 servings a day scaled to a 2000-kcal/d diet. Sodium intake was not measured in the present study.

© 2023 Santos-Beneit G et al. *JAMA Cardiology*.

eTable 4. Baseline characteristics of adolescents included and excluded from the primary analysis (complete-case intention-to-treat analysis) at 2-year follow-up in the SI! Program for Secondary School trial.

	Included in the main analysis			Excluded from the main analysis		
	**Long-term intervention (n=362)**	**Short-term intervention (n=441)**	**Control (n=411)**	**Long-term intervention (n=41)**	**Short-term intervention (n=49)**	**Control (n=22)**
**Families**						
**Region**, n (%)						
Barcelona	266 (73.5)	253 (57.4)	318 (77.4)	28 (68.3)	20 (40.8)	17 (77.3)
Madrid	96 (26.5)	188 (42.6)	93 (22.6)	13 (31.7)	29 (59.2)	5 (22.7)
**Household income**, n (%)						
Low	134 (37.5)	135 (31.0)	123 (30.0)	22 (57.9)	15 (34.9)	7 (33.3)
Average	133 (37.3)	134 (30.7)	119 (29.0)	8 (21.1)	9 (20.9)	7 (33.3)
High	90 (25.2)	167 (38.3)	168 (41.0)	8 (21.1)	19 (44.2)	7 (33.3)
**Parental education**, n (%)						
Low	80 (22.2)	75 (17.2)	77 (18.7)	8 (21.6)	3 (7.0)	3 (13.6)
Medium	158 (43.9)	164 (37.6)	173 (42.1)	13 (35.1)	19 (44.2)	10 (45.5)
High	122 (33.9)	197 (45.2)	161 (39.2)	16 (43.2)	21 (48.8)	9 (40.9)
**Migrant background**, n (%)						
No	197 (55.0)	308 (70.6)	307 (74.7)	26 (68.4)	25 (58.1)	15 (71.4)
Yes	161 (45.0)	128 (29.4)	104 (25.3)	12 (31.6)	18 (41.9)	6 (28.6)
**Adolescents**						
**Age in years**, mean (SD)	12.6 (0.5)	12.5 (0.4)	12.5 (0.4)	12.7 (0.6)	12.5 (0.5)	12.5 (0.3)
**Girls**, n (%)	167 (46.1)	218 (49.4)	197 (47.9)	26 (63.4)	22 (44.9)	12 (54.5)
**Overall CVH score,** mean (SD)	10.3 (1.7)	10.6 (1.5)	10.5 (1.7)	10.5 (1.8)	10.5 (1.7)	10.1 (2.1)

Values are mean (SD) for continuous variables and frequencies (percentages) for categorical variables. CVH, cardiovascular health.

© 2023 Santos-Beneit G et al. *JAMA Cardiology*.

eTable 5. Baseline characteristics of adolescents included and excluded from the primary analysis (complete-case intention-to-treat analysis) at 4-year follow-up in the SI! Program for Secondary School trial.

	Included in the main analysis			Excluded from the main analysis		
	**Long-term intervention (n=328)**	**Short-term intervention (n=401)**	**Control (n=368)**	**Long-term intervention (n=75)**	**Short-term intervention (n=89)**	**Control (n=65)**
**Families**						
**Region**, n (%)						
Barcelona	247 (75.3)	240 (59.9)	288 (78.3)	47 (62.7)	33 (37.1)	47 (72.3)
Madrid	81 (24.7)	161 (40.1)	80 (21.7)	28 (37.3)	56 (62.9)	18 (27.7)
**Household income**, n (%)						
Low	119 (36.6)	123 (30.8)	103 (28.0)	37 (52.9)	27 (33.8)	27 (42.9)
Average	120 (36.9)	123 (30.8)	110 (29.9)	21 (30.0)	20 (25.0)	16 (25.4)
High	86 (26.5)	153 (38.4)	155 (42.1)	12 (17.1)	33 (41.2)	20 (31.7)
**Parental education**, n (%)						
Low	67 (20.5)	69 (17.3)	67 (18.2)	21 (30.0)	9 (11.2)	13 (20.0)
Medium	143 (43.7)	152 (38.1)	152 (41.3)	28 (40.0)	31 (38.8)	31 (47.7)
High	117 (35.8)	178 (44.6)	149 (40.5)	21 (30.0)	40 (50.0)	21 (32.3)
**Migrant background**, n (%)						
No	187 (57.2)	281 (70.4)	276 (75.0)	36 (52.2)	52 (65.0)	46 (71.9)
Yes	140 (42.8)	118 (29.6)	92 (25.0)	33 (47.8)	28 (35.0)	18 (28.1)
**Adolescents**						
**Age in years**, mean (SD)	12.6 (0.4)	12.5 (0.4)	12.5 (0.4)	12.9 (0.6)	12.6 (0.6)	12.7 (0.5)
**Girls**, n (%)	152 (46.3)	197 (49.1)	180 (48.9)	41 (54.7)	43 (48.3)	29 (44.6)
**Overall CVH score,** mean (SD)	10.4 (1.7)	10.7 (1.5)	10.6 (1.7)	10.3 (2.0)	10.5 (1.7)	10.1 (2.0)

Values are mean (SD) for continuous variables and frequencies (percentages) for categorical variables. CVH, cardiovascular health.

© 2023 Santos-Beneit G et al. *JAMA Cardiology*.

eTable 6. Overall CVH change within and between intervention groups for all randomized enrolled participants

	Within group differences[a]			Between group differences[b]			
	**Long-term intervention**	**Short-term intervention**	**Control**	**Control vs Long-term intervention**	**p-value**	**Control vs Short-term intervention**	**p-value**
2-year follow-up	0.13 (-0.24; 0.51)	-0.10 (-0.46; 0.27)	-0.27 (-0.65; 0.10)	0.40 (0.04; 0.77)	0.03	0.18 (-0.19; 0.54)	0.35
4-year follow-up	-0.30 (-0.76; 0.15)	-0.28 (-0.73; 0.17)	-0.39 (-0.85; 0.06)	0.09 (-0.18; 0.36)	0.52	0.11 (-0.16; 0.38)	0.42

[a] Mean marginal within-group differences (change from baseline to follow-up in each group) and 95% CI derived from linear mixed-effects models. Fixed effects were baseline CVH score and randomization group, while region (Madrid or Barcelona) and schools within each region were handled as random effects.
[b] Mean between-group differences (difference between groups in the change from baseline to follow-up) and 95% CI derived from linear mixed-effects models. Fixed effects were baseline CVH score and randomization group, while region (Madrid or Barcelona) and schools within each region were handled as random effects.
Missing data were imputed for both follow-ups using multiple imputation. CVH, cardiovascular health.

© 2023 Santos-Beneit G et al. *JAMA Cardiology*.

eTable 7. Changes in the continuous metrics comprising the CVH score at 2-year follow-up, within and between randomization groups

	Within group differences[a]			Between group differences[b]			
	Long-term intervention	Short-term intervention	Control	Control vs Long-term intervention	p-value	Control vs Short-term intervention	p-value
2-year Follow-up							
**z-BMI**	-0.01 (-0.06; 0.04)	-0.00 (-0.05; 0.04)	0.02 (-0.03; 0.07)	-0.03 (-0.09; 0.03)	0.36	-0.03 (-0.09; 0.04)	0.39
**z-systolic BP**	-0.09 (-0.35; 0.17)	0.19 (-0.07; 0.45)	0.12 (-0.14; 0.37)	-0.21 (-0.60; 0.18)	0.28	0.08 (-0.32; 0.48)	0.69
**z-diastolic BP**	-0.10 (-0.34; 0.14)	-0.18 (-0.42; 0.05)	-0.09 (-0.33; 0.15)	-0.01 (-0.26; 0.24)	0.91	-0.09 (-0.35; 0.16)	0.44
**MVPA**	2.62 (-3.22; 8.45)	-2.20 (-7.72; 3.31)	1.40 (-4.30; 7.09)	1.22 (-6.03; 8.47)	0.73	-3.60 (-10.94; 3.74)	0.32
**Total cholesterol**	-4.92 (-13.01; 3.17)	1.81 (-6.14; 9.77)	-6.46 (-14.50; 1.58)	1.54 (-8.84; 11.92)	0.76	8.27 (-2.29; 18.84)	0.12
**Blood glucose***	-8.71 (-18.65; 1.23)	-6.98 (-16.88; 2.91)	-2.63 (-12.56; 7.30)	-6.08 (-10.61; -1.56)	0.01	-4.35 (-8.90; 0.20)	0.06
**Diet**							
Fruits and vegetables	-0.10 (-0.37; 0.16)	-0.17 (-0.42; 0.08)	-0.33 (-0.59; -0.07)	0.23 (-0.10; 0.55)	0.16	0.16 (-0.17; 0.50)	0.31
Fish	-0.50 (-0.82; -0.17)	-0.60 (-0.90; -0.30)	-0.62 (-0.93; -0.31)	0.13 (-0.35; 0.61)	0.58	0.02 (-0.47; 0.51)	0.94
Sweet beverages	-0.24 (-0.51; -0.07)	-0.34 (-0.49; -0.18)	-0.39 (-0.54; -0.23)	0.15 (-0.10; 0.39)	0.23	0.05 (-0.20; 0.30)	0.69
Cereals	0.00 (-0.14; 0.14)	-0.02 (-0.16; 0.12)	-0.08 (-0.22; 0.06)	0.08 (-0.07; 0.23)	0.28	0.06 (-0.10; 0.21)	0.45

[a] Mean marginal within-group differences (change from baseline to follow-up in each group) and 95% CI derived from linear mixed-effects models. Fixed effects were baseline score and randomization group, whereas region (Madrid or Barcelona) and schools within each region were handled as random effects.
[b] Mean between-group differences (difference between groups in the change from baseline to follow-up) and 95% CI derived from linear mixed-effects models. Fixed effects were baseline score and randomization group, while region (Madrid or Barcelona) and schools within each region were handled as random effects.
The Kenward-Roger method for small sample correction was used.
z-BMI, body mass index z-score; BP, blood pressure; MVPA, moderate-to-vigorous physical activity.
*Although participants were instructed to fast overnight before the assessments, some of them may have had a non-fasting status at the time of measurements.

© 2023 Santos-Beneit G et al. *JAMA Cardiology*.

eTable 8. Changes in the continuous metrics comprising the CVH score at 4-year follow-up, within and between randomization groups

	Within group differences[a]			Between group differences[b]			
	Long-term intervention	Short-term intervention	Control	Control vs Long-term intervention	p-value	Control vs Short-term intervention	p-value
4-year Follow-up							
**zBMI**	-0.01 (-0.08; 0.07)	-0.06 (-0.13; 0.01)	-0.02 (-0.09; 0.05)	0.01 (-0.07; 0.10)	0.74	-0.04 (-0.13; 0.05)	0.34
**zSYS**	0.09 (-0.48; 0.66)	0.23 (-0.34; 0.80)	0.28 (-0.29; 0.85)	-0.19 (-0.48; 0.09)	0.17	-0.05 (-0.33; 0.23)	0.72
**zDIA**	0.02 (-0.19; 0.23)	-0.09 (-0.29; 0.12)	-0.11 (-0.31; 0.10)	-0.09 (-0.29; 0.12)	0.37	-0.11 (-0.31; 0.10)	0.28
**MVPA**	-12.74 (-17.53; -7.94)	-9.99 (-14.54; -5.44)	-11.18 (-15.81; -6.54)	-1.56 (-8.67; 5.56)	0.65	1.19 (-6.10; 8.48)	0.74
**Total cholesterol**	-3.37 (-8.81; 2.06)	-3.99 (-9.28; 1.29)	-9.50 (-14.86; -4.13)	6.12 (-0.66; 12.91)	0.07	5.50 (-1.37; 12.38)	0.11
**Blood glucose***	-9.81 (-16.70; -2.93)	-9.09 (-15.93; -2.24)	-7.37 (-14.24; -0.49)	-2.45 (-5.42; 0.53)	0.10	-1.72 (-4.69; 1.25)	0.24
**Diet**							
Fruits and vegetables	-0.33 (-0.66; 0.01)	-0.10 (-0.42; 0.23)	-0.29 (-0.62; 0.04)	-0.04 (-0.54; 0.46)	0.87	0.19 (-0.32; 0.70)	0.45
Fish	-0.62 (-0.94; -0.31)	-0.51 (-0.81; -0.22)	-0.74 (-1.04; -0.44)	0.11 (-0.35; 0.57)	0.62	0.22 (-0.25; 0.70)	0.33
Sweet beverages	-0.50 (-0.69; -0.32)	-0.39 (-0.57; -0.21)	-0.52 (-0.70; -0.34)	0.01 (-0.26; 0.29)	0.92	0.13 (-0.16; 0.41)	0.36
Cereals	-0.03 (-0.11; 0.06)	-0.05 (-0.13; 0.03)	-0.12 (-0.20; -0.03)	0.09 (-0.03; 0.21)	0.14	0.07 (-0.06; 0.19)	0.26

[a] Mean marginal within-group differences (change from baseline to follow-up in each group) and 95% CI derived from linear mixed-effects models. Fixed effects were baseline score and randomization group, whereas region (Madrid or Barcelona) and schools within each region were handled as random effects.
[b] Mean between-group differences (difference between groups in the change from baseline to follow-up) and 95% CI derived from linear mixed-effects models. Fixed effects were baseline score and randomization group, while region (Madrid or Barcelona) and schools within each region were handled as random effects.
The Kenward-Roger method for small sample correction was used.
z-BMI, body mass index z-score; BP, blood pressure; MVPA, moderate-to-vigorous physical activity.
*Although participants were instructed to fast overnight before the assessments, some of them may have had a non-fasting status at the time of measurements.

eTable 9. Adjusted changes in the overall CVH score and individual CVH metrics at 2-year follow-up, within and between randomization groups

	Within group differences[a]			Between group differences[b]			
	Long-term intervention	Short-term intervention	Control	Control vs Long-term intervention	p-value	Control vs Short-term intervention	p-value
2-year Follow-up							
**Overall CVH score**	0.21 (-0.30; 0.72)	-0.13 (-0.63; 0.37)	-0.35 (-0.86; 0.16)	0.57 (0.14; 0.97)	0.01	0.22 (-0.19; 0.64)	0.28
**Individual metrics**							
Smoking status	-0.34 (-0.59; -0.08)	-0.42 (-0.68; -0.17)	-0.49 (-0.74; -0.23)	0.15 (-0.02; 0.33)	0.09	0.06 (-0.11; 0.24)	0.45
Body mass index	0.00 (-0.07; 0.07)	0.08 (0.01; 0.14)	0.01 (-0.06; 0.08)	-0.01 (-0.06; 0.05)	0.78	0.06 (0.01; 0.12)	0.03
Physical activity	-0.07 (-0.13; -0.01)	-0.09 (-0.15; -0.04)	-0.04 (-0.10; 0.02)	-0.03 (-0.12; 0.06)	0.45	-0.05 (-0.14; 0.04)	0.23
Diet	0.10 (0.05; 0.15)	0.01 (-0.04; 0.06)	-0.03 (-0.08; 0.02)	0.12 (0.05; 0.20)	0.00	0.04 (-0.04; 0.11)	0.33
Blood pressure	0.10 (0.04; 0.16)	0.04 (-0.01; 0.09)	0.01 (-0.04; 0.06)	0.09 (0.01; 0.17)	0.03	0.03 (-0.05; 0.11)	0.47
Total cholesterol	0.06 (-0.12; 0.24)	0.01 (-0.17; 0.18)	0.08 (-0.10; 0.26)	-0.02 (-0.20; 0.16)	0.81	-0.08 (-0.26; 0.10)	0.37
Blood glucose*	0.31 (-0.03; 0.65)	0.27 (-0.07; 0.61)	0.12 (-0.22; 0.46)	0.19 (0.02; 0.36)	0.03	0.15 (-0.02; 0.32)	0.08

[a] Mean marginal within-group differences (change from baseline to follow-up in each group) and 95% CI derived from linear mixed-effects models. Fixed effects were baseline CVH score, gender, age, household income, migrant status and randomization group, whereas region (Madrid or Barcelona) and schools within each region were handled as random effects.
[b] Mean between-group differences (difference between groups in the change from baseline to follow-up)and 95% CI derived from linear mixed-effects models. Fixed effects were baseline CVH score, gender, age, household income, migrant status and randomization group, while region (Madrid or Barcelona) and schools within each region were handled as random effects.
The Kenward-Roger method for small sample correction was used.
CVH, cardiovascular health.
*Although participants were instructed to fast overnight before the assessments, some of them may have had a non-fasting status at the time of measurements.

© 2023 Santos-Beneit G et al. *JAMA Cardiology*.

eTable 10. Adjusted changes in the overall CVH score and individual CVH metrics at 4-year follow-up, within and between randomization groups

	Within group differences[a]			Between group differences[b]			
	Long-term intervention	Short-term intervention	Control	Control vs Long-term intervention	p-value	Control vs Short-term intervention	p-value
4-year Follow-up							
**Overall CVH score**	-0.29 (-1.00; 0.43)	-0.36 (-1.07; 0.35)	-0.51 (-1.23; 0.20)	0.23 (-0.02; 0.47)	0.07	0.16 (-0.08; 0.40)	0.18
**Individual metrics**							
Smoking status	-0.67 (-0.82; -0.51)	-0.74 (-0.89; -0.60)	-0.80 (-0.95; -0.65)	0.13 (-0.05; 0.32)	0.16	0.05 (-0.13; 0.24)	0.55
Body mass index	0.04 (-0.02; 0.11)	0.09 (0.03; 0.15)	0.06 (-0.01; 0.12)	-0.01 (-0.08; 0.06)	0.71	0.04 (-0.03; 0.10)	0.28
Physical activity	-0.29 (-0.39; -0.20)	-0.29 (-0.38; -0.20)	-0.27 (-0.36; -0.17)	-0.03 (-0.13; 0.08)	0.62	-0.02 (-0.12; 0.09)	0.72
Diet	0.06 (0.00; 0.12)	0.04 (-0.01; 0.10)	0.06 (0.00; 0.12)	0.00 (-0.08; 0.09)	0.95	-0.02 (-0.11; 0.07)	0.65
Blood pressure	0.03 (-0.21; 0.27)	0.01 (-0.22; 0.25)	-0.03 (-0.26; 0.21)	0.06 (-0.04; 0.16)	0.25	0.04 (-0.06; 0.14)	0.42
Total cholesterol	0.07 (0.02; 0.13)	0.11 (0.07; 0.16)	0.16 (0.11; 0.21)	-0.09 (-0.16; -0.01)	0.03	-0.05 (-0.12; 0.03)	0.21
Blood glucose*	0.45 (0.26; 0.64)	0.44 (0.25; 0.63)	0.37 (0.18; 0.56)	0.08 (-0.02; 0.18)	0.10	0.07 (-0.03; 0.18)	0.14

[a] Mean marginal within-group differences (change from baseline to follow-up in each group) and 95% CI derived from linear mixed-effects models. Fixed effects were baseline CVH score, gender, age, household income, migrant status and randomization group, whereas region (Madrid or Barcelona) and schools within each region were handled as random effects.
[b] Mean between-group differences (difference between groups in the change from baseline to follow-up) and 95% CI derived from linear mixed-effects models. Fixed effects were baseline CVH score, gender, age, household income, migrant status and randomization group, while region (Madrid or Barcelona) and schools within each region were handled as random effects.
The Kenward-Roger method for small sample correction was used.
CVH, cardiovascular health.
*Although participants were instructed to fast overnight before the assessments, some of them may have had a non-fasting status at the time of measurements.

© 2023 Santos-Beneit G et al. *JAMA Cardiology*.

eTable 11. Main qualitative results from focus groups on teachers and students

Key topic	Discursive analysis	Participant Quotations
Overall view of the project	The participants were asked about their personal experience with the SI! Program. In all cases, without exception, the feedback was positive or very positive.	"The summary would be that it is a positive experience. We could see the maturity of the children, today we had the questionnaires and measurements (...) and you can see, a little bit, their evolution (...) The duration I think it is also *(positive)*, because it allows to see the development in such an important stage. They were children and now they are more or less little men and women. The experience is positive" (Teacher).<br><br>"I think it was very good (...) we have been followed up from the first grade of Secondary school for research on heart health (...) for the young people and I think it is a good initiative, honestly" (Student).<br><br>"I found it interesting because I think that sometimes we are not aware (...) of our health in general, we do not know if these foods are healthy for us or not, and with this program we have been able to learn about this whole world of food and health in general" (Student).
COVID-19	The lockdown in Spring 2020 and hybrid learning in the school year 2020-2021 affected the implementation of the intervention. However, for the students, the pandemic might have been a moment of application of the knowledge and awareness acquired from their participation in this trial.	"I personally, for example, when we were in quarantine, locked up at home, my health habits improved, and I think the program had some influence (...) I didn't eat fruit before, nothing, absolutely nothing, and when they locked us up I started eating more fruit, bananas, apples, and now I eat a lot more, so I think it has helped me, for example, in that aspect" (Student).<br><br>"(...) moments that made you think about your health and you think about how to improve it and it is in those moments when you remember that you have done a program that has led you to control both your mental and physical health, such as diet and you think what they *(teachers)* have explained to you and you know how to use what they have told you" (Student).<br><br>"Their letter from the future *(one of the activities of the Program)*, the 4th-grade teachers have told us that they have loved it (...)the pandemic has affected or helped us in this case (...) on the one hand they have had time to reflect and to be with them alone, but we believe that they have been given the opportunity to reflect on all that, they have had the chance to transmit it, to express it *(in the letter)*, and the truth is that the teachers told us about it in an emotional way" (Teacher).
Intervention duration/intensity	Most of the teachers in the short, more intense intervention group would have liked to implement a	"I think it could be done (better) in 4 years, what could be good is to reduce a little the load per year (...) assigned to specific departments and that the annual load would be lightened (...) so that it would be more distributed" (Teacher).

© 2023 Santos-Beneit G et al. *JAMA Cardiology*.

	longer version of the intervention.	“I think it should be 4 years (...) for me it is important to work (the content) during the 4 years, but adapting and responding a little bit to the needs of the students” (Teacher).<br><br>“I think the continuity of two more years would be positive” (Teacher).
Assessments impact on health awareness	For students, their participation in the trial assessments played a fundamental role in raising health awareness, mostly regarding eating habits. Moreover, families were also influenced.	“When I completed the questionnaires, I thought -wow! - because I haven’t realized that I ate so much sugar in a week, it made me reconsider some things I ate” (Student).<br><br>“I kind of worry a little more about my health because in the aspect of food, when I completed the questionnaires I began to see that there were columns in the survey where I didn’t eat anything, I didn’t eat any of this, any of the other, and at the end, I realized that my eating habits were not very healthy and I have been improving over the years” (Student).<br><br>“When completing these questionnaires there were also some that have to be completed by our family, maybe you could also come to the conclusion with your family that what you were doing or the approach you were following was not correct and you had to change some stuff. And the fact of taking care of oneself, in the end, can end up taking care of the 4 or 5 or 3 that are in a family, or more” (Student).<br><br>“It's something curious and cool to see the change and things that you didn't know you have in your body and also the accelerometer” (Student)<br><br>“I loved that families got information on the results of the measurements and the changes. I must also say that I know one of the parents (...) and he is very happy with the type of tests that have been done on his son, which otherwise would never have been done” (Teacher).<br><br>“The program is good because you imagine (...) how your health is and so on, but you see the results, and you see that it is not quite like that, and you see exactly how you are, and I think it is interesting” (Student).

© 2023 Santos-Beneit G et al. *JAMA Cardiology*.

eTable 12. Overall CVH change within and between intervention groups excluding the blood glucose metric

	Within group differences[a]			Between group differences[b]			
	Long-term intervention	Short-term intervention	Control	Control vs Long-term intervention	p-value	Control vs Short-term intervention	p-value
2-year follow-up	-0.19 (-0.43; 0.05)	-0.40 (-0.64; -0.17)	-0.36 (-0.60; -0.13)	0.18 (-0.18; 0.53)	0.31	-0.04 (-0.41; 0.33)	0.83
4-year follow-up	-0.79 (-1.29; -0.29)	-0.78 (-1.27; -0.29)	-0.78 (-1.27; -0.28)	-0.01 (-0.30; 0.27)	0.95	-0.00 (-0.29; 0.28)	0.97

[a] Mean marginal within-group differences (change from baseline to follow-up in each group) and 95% CI were derived from linear mixed-effects models. Fixed effects were baseline CVH score and randomization group, while region (Madrid or Barcelona) and schools within each region were handled as random effects.
[b] Mean between-group differences (difference between groups in the change from baseline to follow-up) and 95% CI were derived from linear mixed-effects models. Fixed effects were baseline CVH score and randomization group, while region (Madrid or Barcelona) and schools within each region were handled as random effects.
The Kenward-Roger method for small sample correction was used.
CVH, cardiovascular health.

© 2023 Santos-Beneit G et al. *JAMA Cardiology*.

eFigure 1. Examples of intervention material

<table>
<tr><th>Health component</th><th>Classroom activities for students</th><th>Newsletters for families</th></tr>
<tr><td><u>Healthy eating</u></td><td>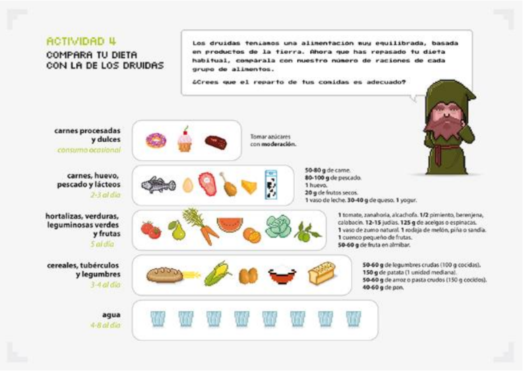
</td><td>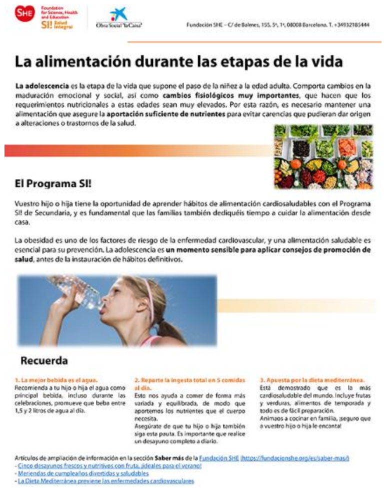
</td></tr>
<tr><td><u>Physical activity</u></td><td>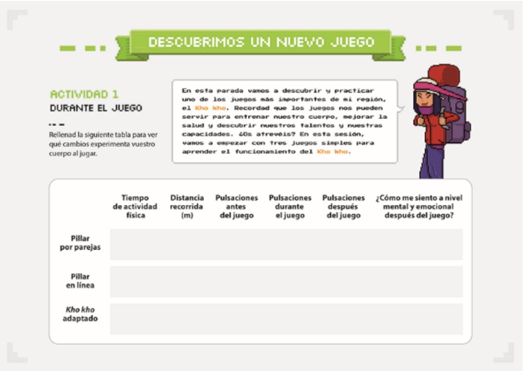
</td><td>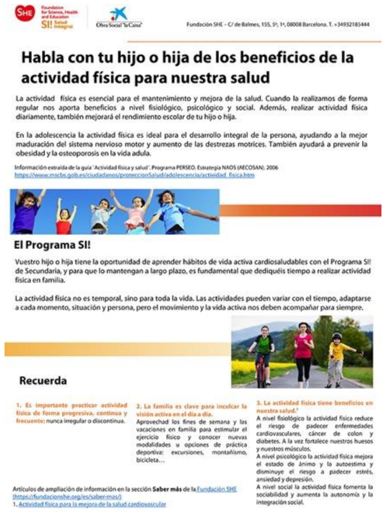
</td></tr>
</table>

© 2023 Santos-Beneit G et al. *JAMA Cardiology*.

<table>
<tr>
<td><u>Substance abuse avoidance</u></td>
<td>
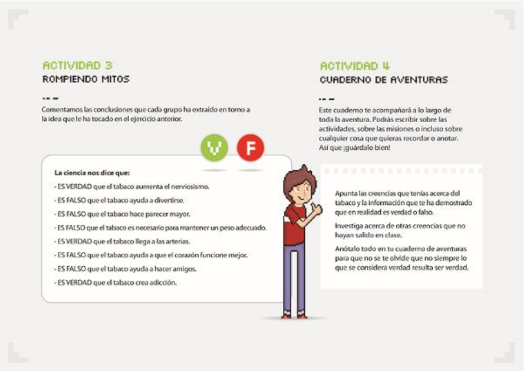

</td>
<td>
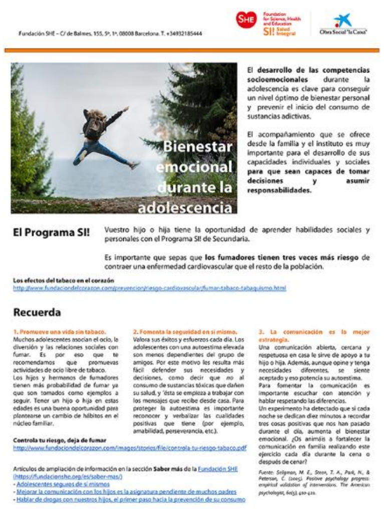

</td>
</tr>
</table>

© 2023 Santos-Beneit G et al. *JAMA Cardiology*.

eFigure 2. Extended Study Flowchart

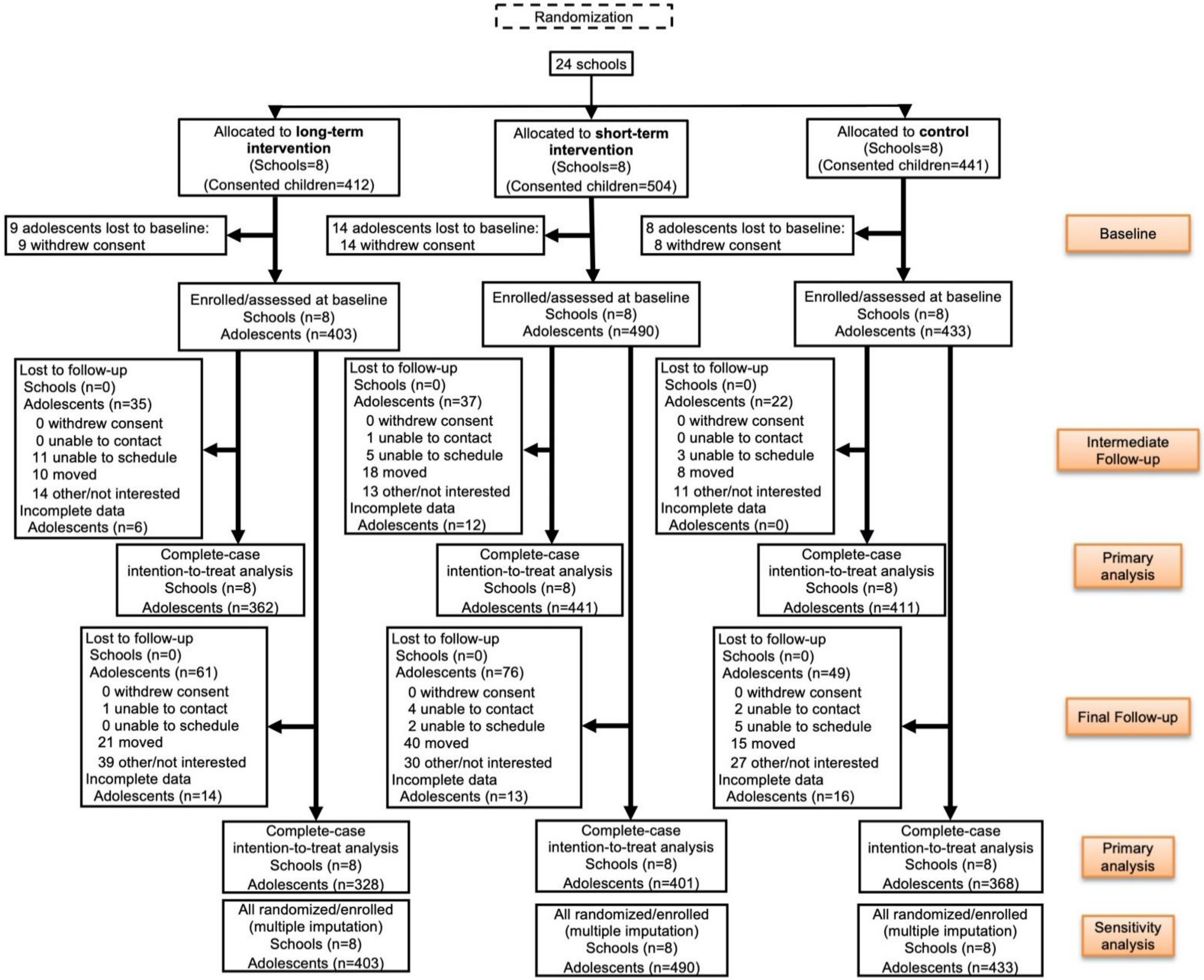

© 2023 Santos-Beneit G et al. *JAMA Cardiology*.

eFigure 3. Change in the overall CVH score at 2-year follow-up according to sociodemographic characteristics

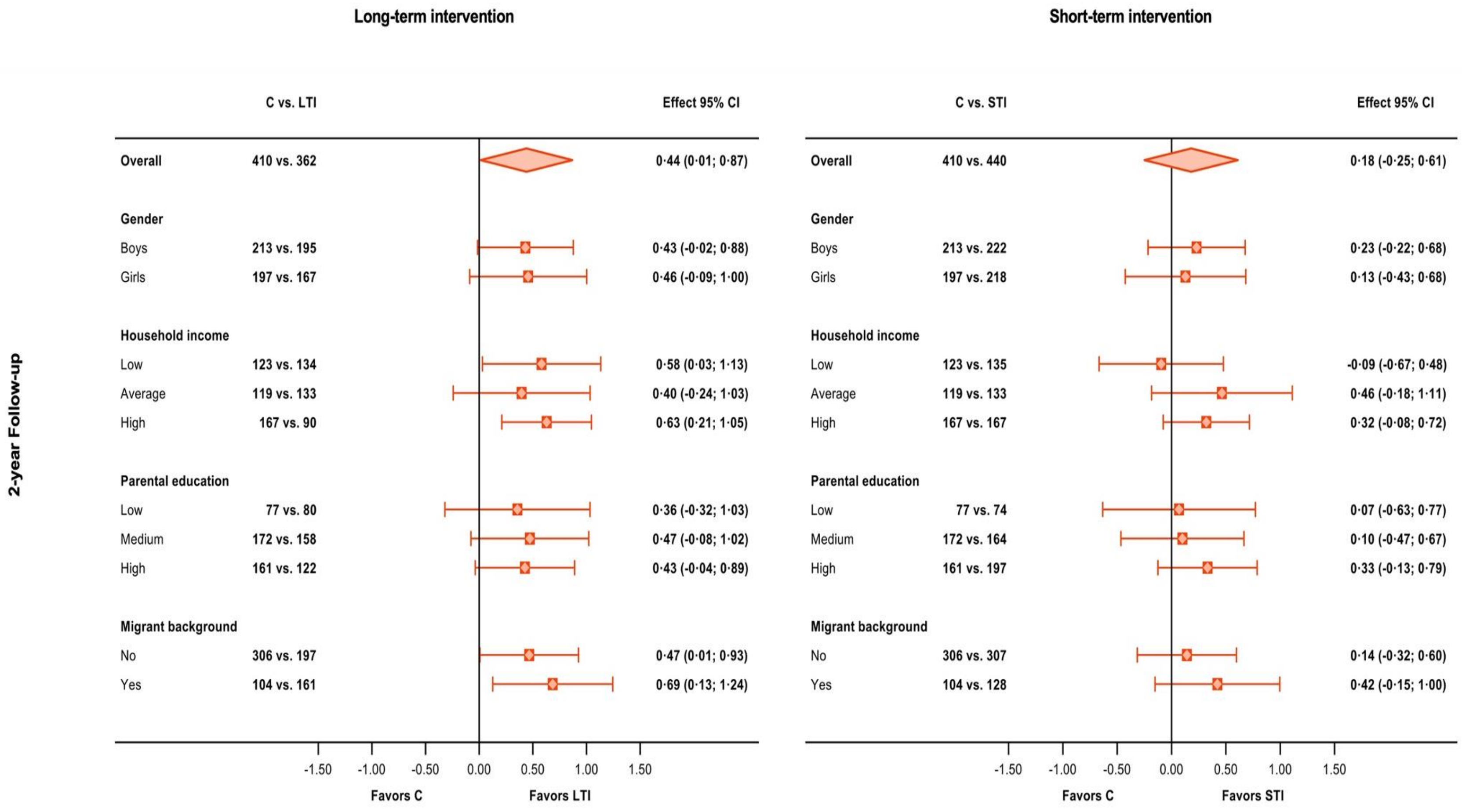

Forest plot representing mean differences (95% confidence interval [CI]) in the overall CVH score changes at 2 years between adolescents in the intervention and control groups, after stratification for selected variables in linear mixed-effects models. Fixed effects were the corresponding baseline CVH score and randomization group, whereas region (Madrid or Barcelona) and schools within each region were handled as random effects. The Kenward-Roger method for small sample correction was used.
LTI, Long-term intervention; STI, Short-term intervention; C, Control.

© 2023 Santos-Beneit G et al. *JAMA Cardiology*.

eFigure 4. Change in the overall CVH score at 4-year follow-up according to sociodemographic characteristics

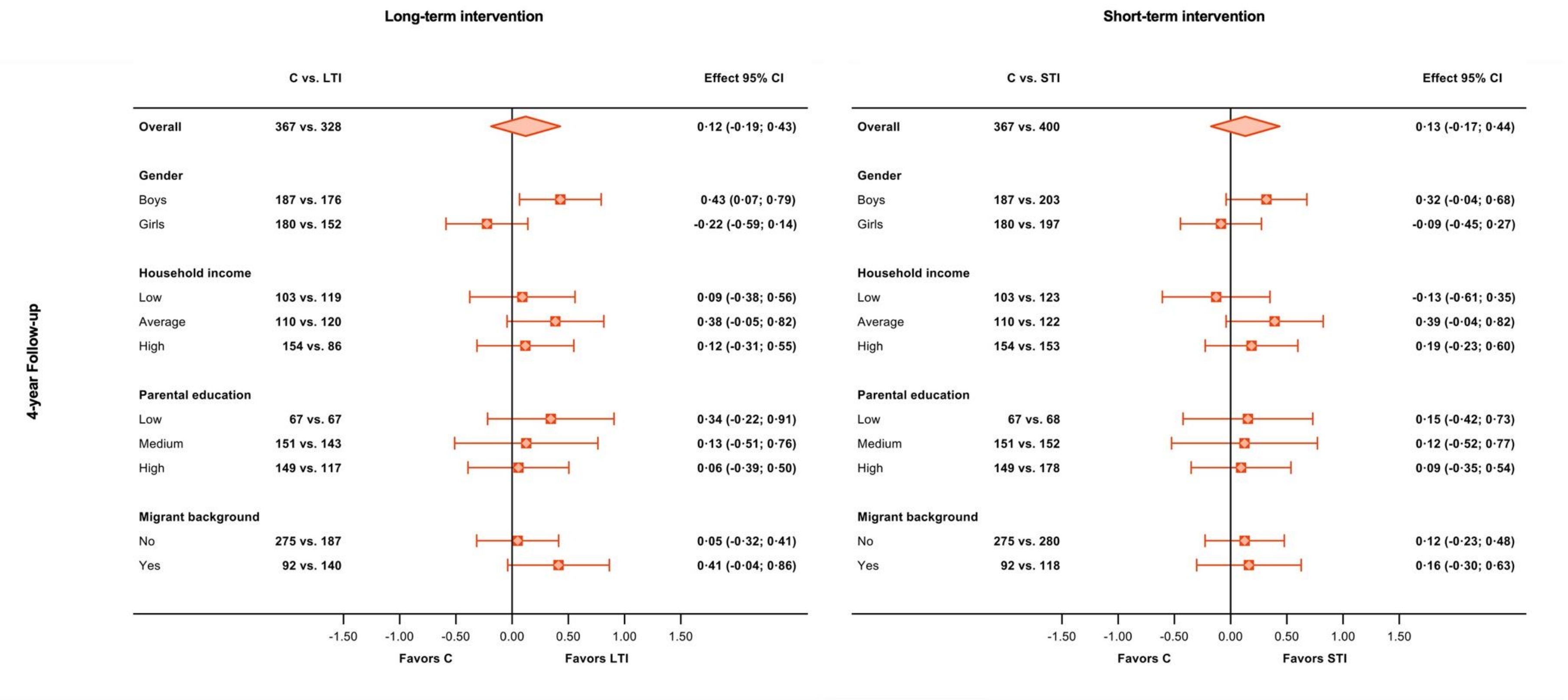

Forest plot representing mean differences (95% confidence interval [CI]) in the overall CVH score changes at 4 years between adolescents in the intervention and control groups, after stratification for selected variables in linear mixed-effects models. Fixed effects were the corresponding baseline CVH score and randomization group, whereas region (Madrid or Barcelona) and schools within each region were handled as random effects. The Kenward-Roger method for small sample correction was used.
LTI, Long-term intervention; STI, Short-term intervention; C, Control.

© 2023 Santos-Beneit G et al. *JAMA Cardiology*.

eReferences

1. Penalvo JL, Santos-Beneit G, Sotos-Prieto M, et al. A cluster randomized trial to evaluate the efficacy of a school-based behavioral intervention for health promotion among children aged 3 to 5. *BMC Public Health*. Jul 15 2013;13:656. doi:10.1186/1471-2458-13-656
2. Inchley J, Currie D, Young T, et al. *Growing up unequal: gender and socioeconomic differences in young people's health and well-being. Health Behaviour in School-aged Children (HBSC) Study: International Report from the 2013/14 Survey*. 2016.
3. Fuster V, Kelly BB. *Promoting Cardiovascular Health in the Developing World: A Critical Challenge to Achieve Global Health.* Institute of Medicine (US) Committee on Preventing the Global Epidemic of Cardiovascular Disease: Meeting the Challenges in Developing Countries; 2010.
4. Krug I, Villarejo C, Jimenez-Murcia S, et al. Eating-related environmental factors in underweight eating disorders and obesity: are there common vulnerabilities during childhood and early adolescence? *Eur Eat Disord Rev*. May 2013;21(3):202-8. doi:10.1002/erv.2204
5. Nguyen-Rodriguez ST, Unger JB, Spruijt-Metz D. Psychological determinants of emotional eating in adolescence. *Eat Disord*. May-Jun 2009;17(3):211-24. doi:10.1080/10640260902848543
6. Bennett J, Greene G, Schwartz-Barcott D. Perceptions of emotional eating behavior. A qualitative study of college students. *Appetite*. Jan 2013;60(1):187-92. doi:10.1016/j.appet.2012.09.023
7. Peláez Gómez de Salazar MJ, Alonso Ojembarrena A, González Martínez F, et al. Obesidad en el adolescente: posibles factores predisponentes a los 6 años de edad. *Anales de Pediatría*. 2007/02/01 2007;66(2):217-218. doi:http://dx.doi.org/10.1016/S1695-4033(07)70370-3
8. Genevieve N. Healya NO. Conducta sedentaria y biomarcadores del riesgo cardiometabólico en adolescentes: un problema científico y de salud pública emergente. . *Rev Esp Cardiol* 2010;
9. Actividad física y salud en la infancia y la adolescencia. Guía para todas las personas que participan en su educación. (Ministerio de Educación y Ciencia) (2006).
10. Luengo Martín MA, Romero Tamames E, Gómez Fragüela JA, Guerra López A, Lence Pereiro M. *La prevención del consumo de drogas y la conducta antisocial en la escuela: análisis y evaluación de un programa*. 2010.
11. Currie C, Zanotti C, Morgan A, et al. *Social determinants of health and well-being among young people. Health Behaviour in School-aged Children (HBSC) study: international report from the 2009/2010 survey*. Vol. (Health Policy for Children and Adolescents, No. 6). 2012.
12. Owens J, Adolescent Sleep Working G, Committee on A. Insufficient sleep in adolescents and young adults: an update on causes and consequences. *Pediatrics*. Sep 2014;134(3):e921-32. doi:10.1542/peds.2014-1696
13. Camila. Modelo ASE para el consumo de fruta en población española. *International Journal of Behavioral Nutrition and Physical Activity*. 2007;
14. Waters E, de Silva-Sanigorski A, Hall BJ, et al. Interventions for preventing obesity in children. *The Cochrane database of systematic reviews*. Dec 07 2011;(12):CD001871. doi:10.1002/14651858.CD001871.pub3
15. Laureati M, Bergamaschi V, Pagliarini E. School-based intervention with children. Peer-modeling, reward and repeated exposure reduce food neophobia and increase liking of fruits and vegetables. *Appetite*. Dec 2014;83:26-32. doi:10.1016/j.appet.2014.07.031
16. Dobbins M, Husson H, DeCorby K, LaRocca RL. School-based physical activity programs for promoting physical activity and fitness in children and adolescents aged 6 to 18. *The Cochrane database of systematic reviews*. 2013;2:CD007651. doi:10.1002/14651858.CD007651.pub2
17. Thomas RE, McLellan J, Perera R. School-based programmes for preventing smoking. *The Cochrane database of systematic reviews*. Apr 30 2013;(4):CD001293. doi:10.1002/14651858.CD001293.pub3
18. Vitoria PD, Salgueiro MF, Silva SA, De Vries H. The impact of social influence on adolescent intention to smoke: combining types and referents of influence. *British journal of health psychology*. Nov 2009;14(Pt 4):681-99. doi:10.1348/135910709X421341
19. Markham WA, Lopez ML, Aveyard P, et al. Mediated, moderated and direct effects of country of residence, age, and gender on the cognitive and social determinants of adolescent smoking in Spain and the UK: a cross-sectional study. *BMC Public Health*. Jun 04 2009;9:173. doi:10.1186/1471-2458-9-173
20. Lana AT. *Evaluación de una intervencion sobre tabaquismo en enseñanza secundaria. Programa ITES*. 2010. http://aunets.isciii.es/ficherosproductos/266/SESCS%20N.2007_07_TABACO_sin.pdf
21. de Vries H, Dijkstra M, Kuhlman P. Self-efficacy: the third factor besides attitude and subjective norm as a predictor of behavioural intentions. *Health Educ Res*. 1988; 3(3):273-282. doi:10.1093/her/3.3.273
22. Fishbein MaA, I. . *Belief, Attitude, Intention and Behavior: An Introduction to Theory and Research.* . 1975.
23. McLaren L, Hawe P. Ecological perspectives in health research. *J Epidemiol Community Health*. Jan 2005;59(1):6-14. doi:10.1136/jech.2003.018044

© 2023 Santos-Beneit G et al. *JAMA Cardiology*.

24. Catalano RF, Berglund ML, Ryan JAM, Lonczak HS, Hawkins JD. Positive youth development in the United States: Research findings on evaluations of positive youth development programs. *Prevention & Treatment*. 2002;5(1):No Pagination Specified-No Pagination Specified. doi:10.1037/1522-3736.5.1.515a
25. Orrit X, Carral V, Rodriguez C, et al. The SI! Program in Secondary Education to promote hearthealthy habits in adolescents. Preliminary results of a gamified intervention. Sport, Physical Education and Performing Arts as tools of social transformation (abstract book 30th FIEP World Congress). Torrents, C. y Sebastiani, E.M. (eds). Barcelona:INDE; 2019:133-135.
26. Health Behaviour in School Aged Children (HBSC-2010) (2012).
27. Kuczmarski RJ, Ogden CL, Guo SS, et al. 2000 CDC Growth Charts for the United States: methods and development. *Vital Health Stat 11*. May 2002;(246):1-190.
28. Rich C, Geraci M, Griffiths L, Sera F, Dezateux C, Cortina-Borja M. Quality control methods in accelerometer data processing: defining minimum wear time. *PLoS One*. 2013;8(6):e67206. doi:10.1371/journal.pone.0067206
29. Chandler JL, Brazendale K, Beets MW, Mealing BA. Classification of physical activity intensities using a wrist-worn accelerometer in 8–12-year-old children. *Pediatric Obesity*. 2016;11(2):120-127. doi:https://doi.org/10.1111/ijpo.12033
30. Barbosa N, Sanchez CE, Vera JA, Perez W, Thalabard JC, Rieu M. A physical activity questionnaire: reproducibility and validity. *J Sports Sci Med*. 2007;6(4):505-18.
31. Bel-Serrat S, Mouratidou T, Pala V, et al. Relative validity of the Children's Eating Habits Questionnaire-food frequency section among young European children: the IDEFICS Study. *Public Health Nutr*. Feb 2014;17(2):266-76. doi:10.1017/s1368980012005368
32. Lanfer A, Hebestreit A, Ahrens W, et al. Reproducibility of food consumption frequencies derived from the Children's Eating Habits Questionnaire used in the IDEFICS study. *Int J Obes (Lond)*. Apr 2011;35 Suppl 1:S61-8. doi:10.1038/ijo.2011.36
33. Pala V, Reisch LA, Lissner L. Dietary Behaviour in Children, Adolescents and Families: The Eating Habits Questionnaire (EHQ). In: Bammann K, Lissner L, Pigeot I, Ahrens W, eds. *Instruments for Health Surveys in Children and Adolescents*. Springer International Publishing; 2019:103-133.
34. Fernández-Ballart JD, Piñol JL, Zazpe I, et al. Relative validity of a semi-quantitative food-frequency questionnaire in an elderly Mediterranean population of Spain. *British Journal of Nutrition*. 2010;103(12):1808-1816. doi:10.1017/S0007114509993837
35. Santos-Beneit G, Sotos-Prieto M, Pocock S, Redondo J, Fuster V, Peñalvo JL. Association Between Anthropometry and High Blood Pressure in a Representative Sample of Preschoolers in Madrid. 10.1016/j.rec.2014.09.002. *Revista Española de Cardiología (English Edition)*. 2015;68(6):477-484. doi:10.1016/j.rec.2014.09.002
36. Flynn JT, Kaelber DC, Baker-Smith CM, et al. Clinical Practice Guideline for Screening and Management of High Blood Pressure in Children and Adolescents. *Pediatrics*. Sep 2017;140(3)doi:10.1542/peds.2017-1904
37. Whitehead SJ, Ford C, Gama R. A combined laboratory and field evaluation of the Cholestech LDX and CardioChek PA point-of-care testing lipid and glucose analysers. *Ann Clin Biochem*. Jan 2014;51(Pt 1):54-67. doi:10.1177/0004563213482890
38. UNESCO. *International Standard Classification of Education, ISCED 2011*. UNE SaCOIfS, editor; 2012.
39. Ministerio de Sanidad SSeI. Encuesta Nacional de Salud de España 2011/12. 2013;
40. Schafer JL. *Analysis of Incomplete Multivariate Data*. Monographs on Statistics and Applied Probability. Chapman & Hall/CRC; 1997.
41. Rubin DB. *Multiple Imputation for Nonresponse in Surveys*. Wiley; 1987.
42. Campbell MK, Piaggio G, Elbourne DR, Altman DG. Consort 2010 statement: extension to cluster randomised trials. *Bmj*. Sep 4 2012;345:e5661. doi:10.1136/bmj.e5661
43. Hoffmann TC, Glasziou PP, Boutron I, et al. Better reporting of interventions: template for intervention description and replication (TIDieR) checklist and guide. *BMJ : British Medical Journal*. 2014;348:g1687. doi:10.1136/bmj.g1687
44. Steinberger J, Daniels SR, Hagberg N, et al. Cardiovascular Health Promotion in Children: Challenges and Opportunities for 2020 and Beyond: A Scientific Statement From the American Heart Association. *Circulation*. Sep 20 2016;134(12):e236-55. doi:10.1161/CIR.0000000000000441

© 2023 Santos-Beneit G et al. *JAMA Cardiology*.
