# Supplementary material for: School-Based Cardiovascular Health Promotion in Adolescents: A Cluster Randomized Clinical Trial
Source: JAMA Cardiol. 2023 Aug 2;8(9):816–24. doi: 10.1001/jamacardio.2023.2231 (PMC10398546; doi:10.1001/jamacardio.2023.2231)
Supplement: Supplement 3. — Data Sharing Statement [file jamacardiol-e232231-s003.pdf]

Data Sharing Statement

Santos-Beneit. School-Based Cardiovascular Health Promotion in Adolescents. *JAMA Cardiol*. Published August 02, 2023. doi:10.1001/jamacardio.2023.2231

Data
Data available: Yes
Data types: Deidentified participant data
How to access data: Data availability to external researchers is restricted to related project proposals upon request to the corresponding author. Based on these premises, de-identified participant data will be available with publication after approval of the proposal by the Steering committee and a signed data sharing agreement.
When available: With publication

Supporting Documents
Document types: Statistical/analytic code, Informed consent form
How to access documents: Upon request to the corresponding author
When available: With publication

Additional Information
Who can access the data: Data availability to external researchers is restricted to related project proposals upon request to the corresponding author. Based on these premises, de-identified participant data will be available with publication after approval of the proposal by the Steering committee and a signed data sharing agreement.
Types of analyses: For research purposes
Mechanisms of data availability: Data availability to external researchers is restricted to related project proposals upon request to the corresponding author. Based on these premises, de-identified participant data will be available with publication after approval of the proposal by the Steering committee and a signed data sharing agreement.
